# Incidence, case fatality, and functional outcome of intracerebral haemorrhage, according to age, sex, and country income level: a systematic review and meta-analysis

**DOI:** 10.1016/j.lanepe.2024.101180

**Published:** 2024-12-13

**Authors:** Axel Wolsink, Maaike P. Cliteur, Charlotte J. van Asch, Hieronymus D. Boogaarts, Ruben Dammers, Gerjon Hannink, Floris H.B.M. Schreuder, Catharina J.M. Klijn

**Affiliations:** a Department of Neurology, Donders Institute for Brain, Cognition and Behaviour, Radboud University Medical Centre, Geert Grooteplein Zuid 10, 6525 GA, Nijmegen, the Netherlands; b Stichting Epilepsie Instellingen Nederland (SEIN), Dr. Denekampweg 20, 8025 BV, Zwolle, the Netherlands; c Department of Neurosurgery, Radboud University Medical Centre, Geert Grooteplein Zuid 10, 6525 GA, Nijmegen, the Netherlands; d Department of Neurosurgery, Erasmus Medical Centre, Erasmus MC Stroke Centre, Dr. Molewaterplein 40, 3015 GD, Rotterdam, the Netherlands; e Department of Medical Imaging, Radboud University Medical Center, Geert Grooteplein Zuid 10, 6525 GA, Nijmegen, the Netherlands

**Keywords:** Intracerebral haemorrhage, Stroke, Incidence, Case fatality, Functional outcome

## Abstract

**Background:**

Intracerebral haemorrhage (ICH) accounts for approximately 28% of all strokes worldwide. ICH has a high case fatality, and only few survivors recover to independent living. Over the past decades, demographic changes, and changes in prevalence and management of risk factors may have influenced incidence. Widespread implementation of stroke units and improved care in general may have affected case fatality and outcome. We aimed to update the evidence on incidence, case fatality, and functional outcome of ICH, according to age, sex, and country income level.

**Methods:**

We systematically searched PubMed and Embase from 2008 to April 2023 for prospective population-based studies on incidence, case fatality, or functional outcome of first-ever ICH. We excluded studies in which less than 80% of cases was confirmed with imaging or autopsy. Quality of the studies was assessed based on the used case finding methods. We used inverse variance-based random-effects meta-analyses to pool the crude incidence, case fatality at 1 month, and the percentage of patients with good functional outcome after 3, 6, or 12 months, as defined by the authors of the individual studies. Time trends were assessed using weighted linear meta-regression. Funnel plots were constructed to study publication bias. The review was registered on PROSPERO (CRD42023413314).

**Findings:**

We identified 70 eligible studies, describing 19,470 ICH patients from 26 different countries. Of these, 62 studies reported on crude incidence, 41 on case fatality, and 10 on functional outcome. Overall crude incidence was 29.2 per 100,000 person-years (95% CI 23.3–36.4; I^2^ = 100%). Incidence was lower in women than in men and increased with age. Incidence was highest in lower-middle income countries, followed by high and upper-middle income countries. Case fatality at 1 month was 35.5% (95% CI 32.3–38.9; I^2^ = 90%). The percentage of patients with good functional outcome (mRS 0–2 in nine studies, mRS 0–3 in one) after 3–12 months was 31.2% (95% CI 24.7–38.6; I^2^ = 76%). We found no time trends in incidence, case fatality, or functional outcome.

**Interpretation:**

Our results demonstrate the persistently high burden and devastating consequences of ICH, stressing the need for better preventive strategies and acute treatments.

**Funding:**

None.

**Research in context**

**Evidence before this study**

Intracerebral haemorrhage (ICH) is the subtype of stroke with the most devastating consequences. We have previously performed a systematic review and meta-analysis of studies published between 1980 and 2008, which showed an overall incidence of 24.6 per 100,000 person-years and a case fatality of 40.4%. There was insufficient data to perform a meta-analysis on functional outcome. Since then, demographic changes and differences in prevalence and management of vascular risk factors may have influenced the incidence of ICH. With increasing awareness that ICH is a medical emergency that requires early and intensive bundled care, case fatality and functional outcome may have changed. A more recent systematic review and meta-analysis reported an overall incidence of 26.5 per 100,000 person-years, with highest incidences in lower-middle income countries. It did not focus on incidence in relation to age or sex. Another systematic review showed a 1-month case fatality of 36.3%, but did not describe case fatality by age, sex, or country income level. A meta-analysis of functional outcome has not yet been performed. We therefore performed a new systematic review of population-based and prospective studies on incidence, case fatality, or functional outcome of first-ever ICH, according to age, sex, and country income level. We searched PubMed and Embase for studies published from January 1 2008 to April 18 2023, using a search strategy combining key words for population-based studies, terms for intracerebral haemorrhage, and words for incidence, case fatality, or functional outcome. There were no language restrictions. We excluded paediatric populations or studies in which other types of stroke or intracranial haemorrhage were included, when less than 80% of cases were confirmed by imaging or autopsy, or when more than 5% of the cohort was excluded due to missing data.

**Added value of this study**

We have analysed a total of 19,470 patients with ICH in a total of 19,025,211 people from 70 different studies and 26 different countries. We found an overall crude incidence of 29.2 per 100,000 person-years, a persistently high 1-month case fatality of 35.5%, and 31.2% of patients had a good functional outcome. We conclude that incidence of ICH remains high, and outcome remains poor. In contrast to previous reviews, we have analysed the incidence and case fatality by age, sex, and country income level. Incidence rises with increasing age and is higher in men than women. We found the highest incidence in lower-middle income countries, followed by high and upper-middle income countries. Case fatality did not differ between men and women, and seems more favourable in higher income countries. Information from low income countries was scarce.

**Implications of all the available evidence**

The burden of ICH remains high and the current estimates of incidence, case-fatality and functional outcome underline the urgent need for better preventive strategies and new acute treatments in ICH. As the incidence in lower income countries is relatively high and information on case fatality and functional outcome is scarce, there is a large need for further studies in these regions in order to monitor implemented strategies and diminish the burden of ICH worldwide.

## Introduction

Intracerebral haemorrhage (ICH) is the second most common cause of stroke, accounting for approximately 28% of all strokes worldwide.^1^ ICH is the most devastating subtype of stroke, with a high case fatality and the highest number of disability-adjusted life-years (DALYs).^1^^,^^2^

The Global Burden of Disease (GBD) Study showed a worldwide increase in absolute number of ICH from 1990 to 2019, but a reduction in age-standardized ICH incidence.^1^ Whereas the GBD Study estimates are based on diverse sources, including surveys, hospital databases, and vital statistics, the most reliable and comparable estimates of incidence are provided by prospective and population-based cohort studies.^3^ Prospective population-based cohort studies may also provide the most accurate information on case fatality and functional outcome, which are not part of the GBD Study. However, results of these studies vary. For example, a decrease in incidence over the past decades has been reported in Brazil and Portugal,^4^^,^^5^ no change in incidence was noted in Sweden between 2001 and 2015,^6^ and incidence increased in a rural area in China between 1992 and 2012.^7^ On top of this regional heterogeneity, varying trends have also been reported for different age groups and sexes.^8^, ^9^, ^10^ Diverging trends in these subgroups may be concealed by an overall stable incidence. As for case fatality, an overall decreasing trend has been suggested, although reported trends appear to vary between different age groups.^4^^,^^10^^,^^11^ Data on functional outcome after ICH is scarce^12^

In a previous systematic review and meta-analysis of 36 studies published between 1980 and 2008, we have reported an overall incidence of ICH of 24.6 per 100,000 person-years and a 1-month case fatality of 40.4%.^2^ Demographic changes, changes not only in prevalence but also management of cardiovascular risk factors, including air pollution,^13^ and changes in antithrombotic medication, may have influenced the overall incidence since then. Although proven treatment options for ICH are still limited, widespread implementation of stroke units and acute care bundles, and improved care in general may have affected case fatality and functional outcome. Other recent systematic reviews have reported on global incidence,^14^ 1-month case fatality,^15^ or long-term survival of ICH,^14^^,^^15^ but not in relation to age, sex, or country income level. Functional outcome after ICH has only been described to a limited extent and a meta-analysis has not been performed.^12^ We therefore aimed to provide a comprehensive and up-to-date systematic review and meta-analysis on the incidence, case fatality and functional outcome of ICH over the last 15 years, in relation to age, sex, and country income level.

## Methods

This systematic review and meta-analysis was conducted and reported according to the current Preferred Reporting Items for Systematic Reviews and Meta-Analyses (PRISMA) guidelines,^16^ and follows a pre-specified protocol, published on PROSPERO on April 13th 2023 (CRD42023413314).

### Search strategy and selection criteria

We searched PubMed and Embase for population-based studies on the epidemiology of ICH published between January 2008 and April 18th 2023. The first part of the search strategy consisted of keywords for population-based studies, followed by terms for incidence, case fatality and functional outcome, and concluded with terms for ICH (Appendix). Additional studies were identified from reference lists of eligible studies and available reviews. There were no restrictions on language.

We included prospective and population-based studies reporting on the incidence, case fatality, or functional outcome of ICH. Only studies regarding first-ever ICH, or from which first-ever ICH could be extracted separately, and with designs that allowed extraction or calculation of the outcomes of interest, were included.

We excluded studies in which ICH was not described as a separate entity (e.g. mixed stroke studies) or when other types of intracranial haemorrhage (e.g. subarachnoid haemorrhage) were included. When in doubt, we contacted the authors to clarify whether other types of intracranial haemorrhage had been included. Studies with case finding based solely on ICD-codes, studies in which less than 80% of the stroke cases were confirmed with imaging or autopsy,^3^ studies in which more than 5% of the cohort was excluded due to missing data or loss to follow-up, and studies selecting a paediatric population were excluded as well. We did not set specific criteria for the starting date or total study duration.

All unique records of potentially eligible studies were imported into Covidence (www.covidence.org). Titles and abstracts were independently screened by two authors (AW and MC). Conflicts were resolved by a third author (FS). The remaining articles were independently read in full by the same two authors (AW and MC) and conflicts were resolved by discussion with a third author (FS). In case of overlapping cohorts, we selected the article with the most complete information on the outcomes of interest, followed by the article with the largest number of person-years. Articles reporting complementary information on the same cohort were selected as well. In case an article reported on multiple time periods of a cohort, we extracted the most recent period to prevent overrepresentation. When one study mentioned multiple regions within the same country, we combined the incidences of these regions.

### Data extraction

Two authors (AW and MC) independently extracted the data using a standardised digital and piloted data extraction form. Disagreements were resolved by discussion and, if necessary, in a consensus meeting with a third author (FS).

For each eligible study, we recorded the definition used for ICH, and whether haemorrhage due to secondary causes (e.g. macrovascular cause, malignancy or trauma) was included. Furthermore, we extracted data on study period, midyear of the study, study region, country income level according to the World Bank's country classification at the midyear of the study,^17^ demographic data of the study population, case finding methods, proportion of cases confirmed with imaging or autopsy, number of incident cases, total number of person-years, number of patients who were deceased at one month, and data on functional outcome. If available, data on age- and sex-specific incidence and outcome were also extracted.

If needed, we approached authors to provide us with additional information on the overall crude incidence or total number of person-years. Additionally, we contacted authors when the incidence was only depicted in a figure without the exact numbers. We have received unpublished data on overall incidence and age- and sex-specific cases from nine cohorts.^18^, ^19^, ^20^, ^21^, ^22^, ^23^, ^24^, ^25^, ^26^

We did not use a standardised quality assessment tool to assess the quality of the included studies, since there is no existing tool satisfactory suitable for studies investigating incidence.^27^ Instead, we assessed the quality of each study based on the case finding methods used. Studies were qualified as high quality when they were in concordance with the Stroke-Steps manual proposed by the WHO^28^ or complied to the (updated) criteria for ‘ideal’ stroke incidence studies as proposed by Sudlow and Warlow^3^ or Feigin.^29^

### Data analysis

The primary outcome was the crude incidence of ICH per 100,000 person-years. Secondary outcomes were 1-month case fatality and 12-month functional outcome. We defined 1-month case fatality as the percentage of patients who were deceased at one month after the event. Regarding functional outcome, we have made adjustments from the prespecified study protocol. Instead of using the definitions for good functional outcome that we prespecified in our PROSPERO protocol, we adopted the definition for good functional outcome used by the authors of the individual studies. Additionally, we used data at six or three months when 12-month outcome was unavailable.

We computed the crude incidence per 100,000 person-years and 95% confidence interval (CI) for each study, and pooled these using an inverse variance-based random-effects model. A similar approach was used to pool the 1-month case fatality and 12-month functional outcome. Pooled estimates were calculated using log (incidence) or logit transformation (case fatality and functional outcome), and were subsequently back-transformed.

To assess the effect of age, we performed inverse variance-based random-effects meta-analyses for different age groups (mid-decade bands) and calculated incidence rate ratios (IRR) and risk ratios (RR) and their 95% CIs, using the 45–54 years age group as the reference. Data on patients younger than 45 years of age were combined. Likewise, we determined IRRs and RRs for sex, using men as the reference, and country income levels (high, upper-middle, lower-middle, and low), using high income countries as the reference.

We evaluated potential time trends for incidence, case fatality and functional outcome at the midyear of each study using a weighted linear meta-regression, with the inverse of the standard error as weight. We only performed meta-regression analyses when there were at least ten studies available reporting on a specific outcome. As a post-hoc analysis, we calculated the IRR and annual percent change when multiple time periods within one region were reported.

We assessed heterogeneity across the studies using I^2^, τ^2^, and prediction intervals. Heterogeneity was categorised as follows according to the Cochrane methodology: 0%–40%, heterogeneity that might not be important; 30%–60%, moderate heterogeneity; 50%–90%, substantial heterogeneity; and 75%–100% considerable heterogeneity. To study sources of heterogeneity, we performed a subgroup analysis based on the cohort's geographical region, besides the earlier mentioned subgroup analysis on country income level. Furthermore, we performed meta-regression on mean age and proportion of men in the study population. We performed sensitivity analyses including only studies with high quality. In post-hoc sensitivity analyses, we included only studies with a midyear of 2003 or later, and studies without age limitations. Funnel plots were constructed to study publication bias.

All statistical analyses were executed using R (version 4.1.3, R Foundation for Statistical Computing, Vienna, Austria) using packages ‘meta’ and ‘metafor’.

### Role of the funding source

There was no funding source for this study.

## Results

**Fig. 1:**
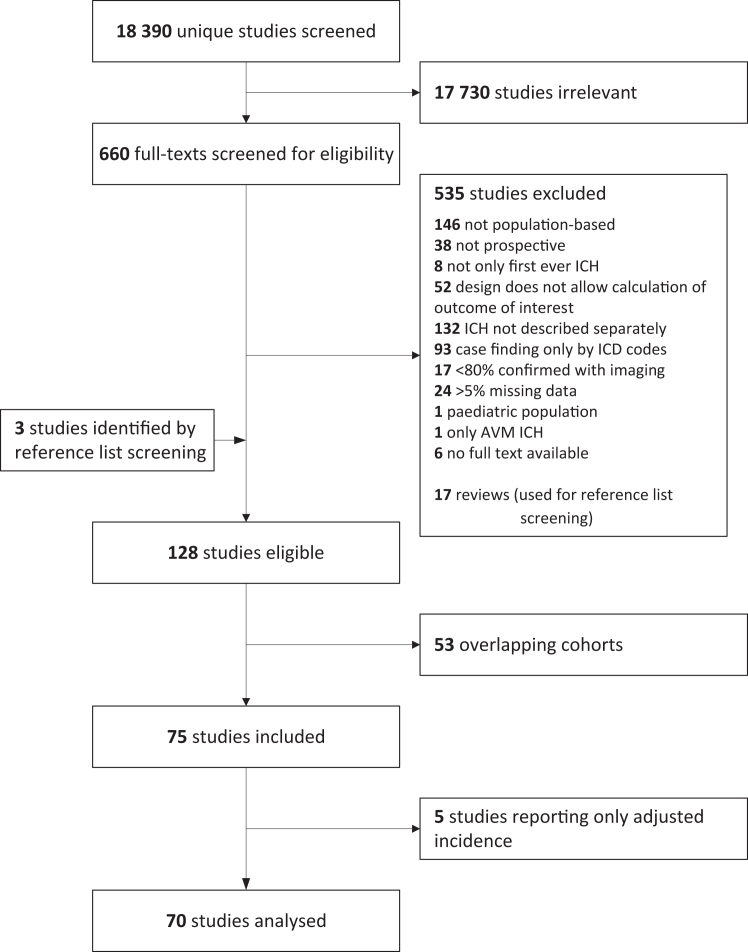
Flow chart of study selection.

We identified 18,390 unique studies, of which 17,730 were deemed irrelevant after screening of title and abstract (Fig. 1). After full text screening of the remaining studies and exclusion of studies with overlapping cohorts, we included 75 studies.^4^, ^5^, ^6^^,^^11^^,^^18^, ^19^, ^20^, ^21^, ^22^, ^23^, ^24^, ^25^, ^26^^,^^30^, ^31^, ^32^, ^33^, ^34^, ^35^, ^36^, ^37^, ^38^, ^39^, ^40^, ^41^, ^42^, ^43^, ^44^, ^45^, ^46^, ^47^, ^48^, ^49^, ^50^, ^51^, ^52^, ^53^, ^54^, ^55^, ^56^, ^57^, ^58^, ^59^, ^60^, ^61^, ^62^, ^63^, ^64^, ^65^, ^66^, ^67^, ^68^, ^69^, ^70^, ^71^, ^72^, ^73^, ^74^, ^75^, ^76^, ^77^, ^78^, ^79^, ^80^, ^81^, ^82^, ^83^, ^84^, ^85^, ^86^, ^87^, ^88^, ^89^, ^90^, ^91^ Five studies only reported adjusted incidences, but could not provide details on crude incidence, and were excluded from the analyses.^35^^,^^48^^,^^50^^,^^61^^,^^91^ The remaining 70 studies described a total of 19,470 ICH patients in a population of 19,025,211 people from 26 different countries. Table 1 shows the characteristics of these studies. There were 54 studies from high income countries,^5^^,^^6^^,^^11^^,^^18^, ^19^, ^20^, ^21^, ^22^, ^23^^,^^25^^,^^26^^,^^31^^,^^32^^,^^34^^,^^37^, ^38^, ^39^^,^^43^^,^^44^^,^^46^^,^^47^^,^^49^^,^^51^, ^52^, ^53^, ^54^, ^55^, ^56^, ^57^^,^^59^^,^^60^^,^^62^, ^63^, ^64^, ^65^, ^66^, ^67^^,^^69^, ^70^, ^71^^,^^73^^,^^74^^,^^76^, ^77^, ^78^, ^79^, ^80^^,^^82^, ^83^, ^84^, ^85^, ^86^^,^^88^^,^^89^ ten from upper-middle income countries,^4^^,^^24^^,^^30^^,^^36^^,^^41^^,^^42^^,^^58^^,^^68^^,^^81^^,^^87^ five from lower-middle income countries,^33^^,^^45^^,^^72^^,^^75^^,^^90^ and one from a low income country.^40^ The midyears of the studies ranged from 1982 to 2019. The definitions of ICH used are shown in Supplementary Table S1. The median percentage of cases confirmed with imaging or autopsy was 97.3% (IQR 93.7–99.6) (Supplementary Table S1). There were 36 studies of high quality (Supplementary Table S2).^4^, ^5^, ^6^^,^^11^^,^^20^^,^^25^^,^^31^^,^^33^^,^^34^^,^^36^^,^^38^^,^^39^^,^^41^^,^^43^, ^44^, ^45^^,^^51^, ^52^, ^53^^,^^56^, ^57^, ^58^, ^59^^,^^63^^,^^70^^,^^71^^,^^75^^,^^76^^,^^78^, ^79^, ^80^, ^81^^,^^85^^,^^86^^,^^89^^,^^90^ There were no age limitations in 47 studies.^4^, ^5^, ^6^^,^^11^^,^^20^^,^^25^^,^^30^^,^^31^^,^^33^^,^^34^^,^^36^^,^^38^^,^^39^^,^^41^^,^^43^, ^44^, ^45^^,^^51^, ^52^, ^53^, ^54^^,^^56^, ^57^, ^58^, ^59^^,^^62^, ^63^, ^64^, ^65^^,^^68^^,^^70^^,^^71^^,^^73^, ^74^, ^75^, ^76^^,^^78^, ^79^, ^80^, ^81^, ^82^^,^^84^, ^85^, ^86^^,^^88^, ^89^, ^90^ Funnel plots suggested no relevant publication bias (Supplementary Figures S1–S3).

**Table 1:** Characteristics of included studies.

Region, country	Midyear of study	Patients with intracerebral haemorrhage	Number of person-years	Age limits	Case finding methods	Incidence rate ratio women vs men (95% CI)
Framingham, United States^62^	1982	129	301,282	na	L	0.96 (0.68–1.36)
Northern Finland, Finland^77^	1990	26	542,140	<50	CDL	–
Hisayama, Japan^b^^,^^18^	1994	53	32,854	≥40	AGJL	–
Takashima, Japan^54^	1995	315	649,611	na	ADM	1.09 (0.87–1.36)
Malmö, Sweden^60^	1996	205	621,143	40–65	AGIJ	–
L'Aquila, Italy^a^^,^^78^	1996	549	1,488,225	na	ABDGHIJ	1.15 (0.97–1.36)
Malmö, Sweden^88^	1997	474	–	na	AGIJ	–
Dijon, France^a^^,^^11^	1998	531	–	na	ABCDG	–
Melbourne, Australia^a^^,^^85^	1998	151	604,000	na	ACDGHIJK	1.01 (0.73–1.39)
Porto, Portugal^a^^,^^38^	1999	111	246,667	na	ABDEGIJLM	–
Dijon, France^a^^,^^43^	1999	530	3,896,484	na	ABDGIJ	0.93 (0.79–1.11)
12 regions, Japan^66^	1999	102	133,640	30–69	AL	–
Perth, Australia^a^^,^^51^	2001	19	143,417	na	ACDGI	–
Iquique, Chile^a^^,^^58^	2001	58	396,712	na	ABDGIJK	0.67 (0.40–1.14)
Erlangen, Germany^a^^,^^b^^,^^20^	2002	374	1,632,197	na	ADGIM	1.08 (0.88–1.33)
Puglia, Italy^64^	2002	24	77,470	na	ACDGI	–
Puglia, Italy^65^	2002	24	77,470	na	ACDG	0.69 (0.31–1.55)
Tromsø, Norway^37^	2003	226	453,152	≥30	ACD	0.78 (0.60–1.01)
Ohasama, Japan^69^	2004	54	20,839	≥60	ACDL	–
Ferrara, Italy^47^	2005	10	323,250	15–44	ACDG	0.43 (0.11–1.67)
Hisayama, Japan^b^^,^^19^	2005	22	20,561	≥40	AGJL	–
Ohio/Kentucky, United States^b^^,^^21^	2005	321	1,003,125	≥20	ACDGIJ	–
Oxford, England^a^^,^^63^	2005	54	–	na	ABCDEGJM	–
Texas, United States^b^^,^^22^	2005	734	11,632,330	≥45	ACDGJ	–
Joinville, Brazil^a^^,^^36^	2006	94	989,474	na	ACDGJ	0.76 (0.51–1.14)
Aosta, Italy^a^^,^^39^	2006	146	626,609	na	ABCDG	0.72 (0.52–0.99)
Mumbai, India^40^	2006	68	313,722	≥25	ADGIJKL	–
5 regions, Spain^a^^,^^44^	2006	350	1,440,979	na	ADGI	–
Northern Manhattan, United States^b^^,^^23^	2006	43	47,080	≥40	L	–
Dublin, Ireland^a^^,^^53^	2006	56	294,529	na	ABDEGIJ	1.12 (0.66–1.89)
Iwate, Japan^73^	2006	999	1,176,678	na	AIJ	0.86 (0.76–0.98)
Mashhad, Iran^a^^,^^33^	2007	79	450,229	na	ADGIJKL	0.77 (0.49–1.20)
Mashhad, Iran^a^^,^^45^	2007	80	450,229	na	ADFGIJKL	–
8 regions, United States^49^	2008	62	156,876	≥45	L	–
Udine, Italy^a^^,^^52^	2008	95	306,624	na	ABDGHJ	1.36 (0.90–2.05)
Inner Mongolia, China^72^	2008	44	23,292	≥20	L	–
Ludwigshafen, Germany^74^	2008	152	812,834	na	AGIJ	–
Varaždin, Croatia^a^^,^^76^	2008	123	368,230	na	ACDGIJ	0.65 (0.45–0.93)
Tianjin, China^a^^,^^90^	2009	106	100,157	na	ADGL	0.81 (0.55–1.19)
10 regions, China^b^^,^^24^	2010	7440	4,723,255	35–74	ACDL	–
Porto, Portugal^a^^,^^5^	2010	43	204,424	na	ABCDEGIJM	–
Joinville, Brazil^a^^,^^41^	2010	37	–	na	ACDGJ	–
Adelaide, Australia^a^^,^^59^	2010	26	148,027	na	ABCDGIJ	1.54 (0.70–3.39)
South Australia, Australia^a^^,^^70^	2010	27	192,072	na	ABCDGIJ	0.91 (0.43–1.94)
Bagheria, Italy^32^	2011	13	19,800	≥65	ACDG	0.38 (0.12–1.21)
Lothian, Scotland^a^^,^^80^	2011	128	711,111	na	ABCDEJ	–
Lesvos, Greece^82^	2011	25	86,505	na	ABDGJM	–
Shiga, Japan^84^	2011	551	1,400,745	na	ADE	0.94 (0.79–1.11)
Evros, Greece^a^^,^^86^	2011	83	119,805	na	ABDEGIJM	0.77 (0.50–1.18)
Lille, France^46^	2012	479	–	≥35	ABDI	–
Auckland, New Zealand^a^^,^^56^	2012	211	1,110,526	na	ADEGIJ	1.06 (0.81–1.39)
Lille, France^67^	2012	378	879,070	≥35	ABCDI	–
Martinique^a^^,^^89^	2012	84	370,854	na	ACDGJM	0.38 (0.24–0.61)
L'Aquila, Italy^a^^,^^79^	2012	115	596,430	na	ABDGJM	1.15 (0.80–1.66)
Tangshan, China^87^	2012	880	1,035,294	18–98	ACDL	–
Joinville, Brazil^a^^,^^4^	2013	84	1,073,318	na	ACDGJ	–
Ludhiana, India^a^^,^^75^	2013	290	1,074,074	na	ABDFGJ	–
Nagahama, Japan^83^	2013	39	78,008	30–74	AD	–
Tandil, Argentina^a^^,^^34^	2014	54	261,182	na	ABFGJK	1.09 (0.59–1.72)
Atahualpa, Ecuador^42^	2014	3	2499	≥40	ADL	–
Dijon, France^a^^,^^b^^,^^25^	2014	120	460,998	na	ABCDGIJ	0.76 (0.53–1.08)
Tartu, Estonia^55^	2015	14	291,667	15–54	ACDGJM	0.51 (0.17–1.53)
Ohio/Kentucky, United States^b^^,^^26^	2015	332	–	≥20	ACEGJ	–
Algarve, Portugal^a^^,^^71^	2015	82	280,081	na	ABCEIJ	0.50 (0.32–0.78)
Lund, Sweden^a^^,^^6^	2016	60	276,400	na	ACEGJ	–
Ñuble, Chile^a^^,^^57^	2016	111	493,464	na	ABCDGJKM	0.93 (0.64–1.36)
Matao, Brazil^68^	2016	10	78,890	na	ABDEGIJ	0.42 (0.11–1.62)
4 regions, Brazil^a^^,^^81^	2016	105	1,246,129	na	ACDGJ	–
Örebro, Sweden^a^^,^^31^	2017	36	150,291	na	ACDEJ	–
Buenos Aires, Argentina^30^	2019	17	92,592	na	ADFGHIJK	1.86 (0.69–5.04)

A, all hospitals in the region; B, review radiology requests/reports; C, ICD-codes; D, death certificates; E, autopsy reports; F, verbal autopsy reports; G, general practitioners; H, rehabilitation centres; I, nursing homes; J, outpatients clinics/health centres; K, media attention; L, survey/home visit; M, emergency/ambulance/on-call medical services;

na, not applicable.

a Study categorised as high-quality.

b Additional data provided by the authors.

**Fig. 2:**
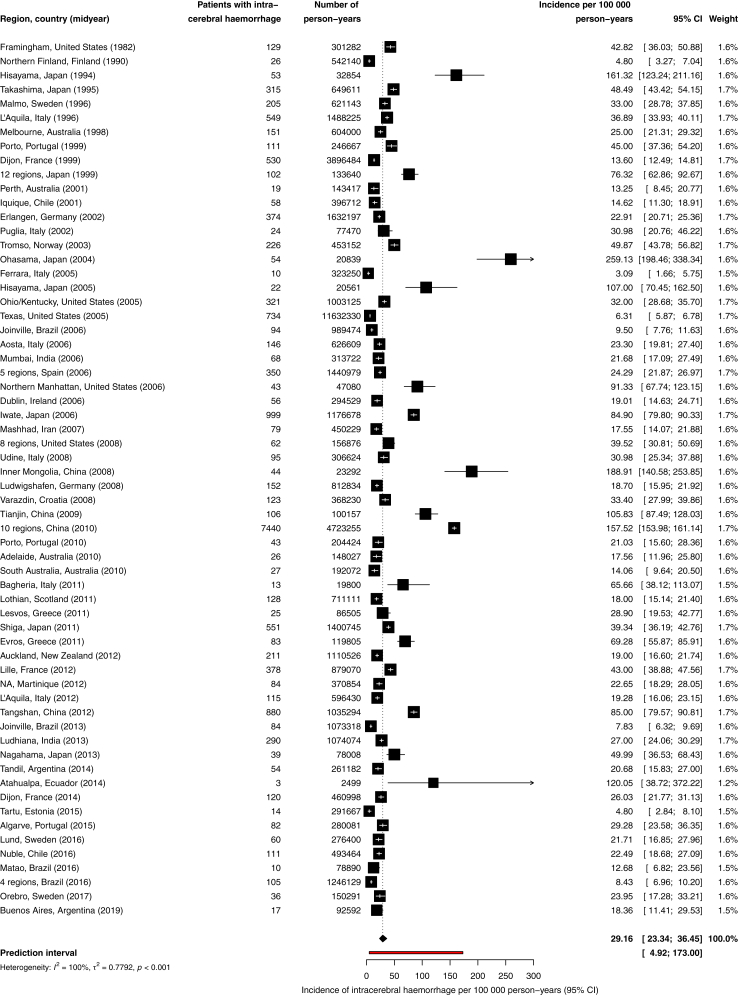
**Incidence of intracerebral haemorrhage per 100,000 person-years**. Size of the point estimates is proportional to the weight of the studies. The weight of the studies is determined by the inverse of the variance of the study plus the between-study variance.

Overall, 62 studies with a total of 17,459 patients with ICH and 48,783,923 person-years of observation reported on crude incidence.^4^, ^5^, ^6^^,^^18^, ^19^, ^20^, ^21^, ^22^, ^23^, ^24^, ^25^^,^^30^, ^31^, ^32^, ^33^, ^34^^,^^36^, ^37^, ^38^, ^39^, ^40^^,^^42^, ^43^, ^44^^,^^47^^,^^49^^,^^51^, ^52^, ^53^, ^54^, ^55^, ^56^, ^57^, ^58^, ^59^, ^60^^,^^62^^,^^65^, ^66^, ^67^, ^68^, ^69^, ^70^, ^71^, ^72^, ^73^, ^74^, ^75^, ^76^, ^77^, ^78^, ^79^, ^80^, ^81^, ^82^, ^83^, ^84^, ^85^, ^86^, ^87^^,^^89^^,^^90^ The overall crude incidence of ICH in the study period was 29.2 per 100,000 person-years (95% CI 23.3–36.4; Fig. 2). Heterogeneity among the studies was considerable (I^2^ = 100%; relatively high τ^2^ and wide prediction interval). We found higher incidence rates in Asian countries (Supplementary Figure S4), but proportion of men did not contribute to this heterogeneity (−3.51% decrease in incidence for every percent increase in proportion of men, 95% CI −8.22 to 1.44; 30 studies). The effect of mean age of the study population on heterogeneity could not be assessed, since this was available in only 8 studies. Sensitivity analysis including only the 32 studies of high quality resulted in a crude incidence of 22.1 per 100,000 person-years (95% CI 18.3–26.6).^4^, ^5^, ^6^^,^^20^^,^^25^^,^^31^^,^^33^^,^^34^^,^^36^^,^^38^^,^^39^^,^^43^^,^^44^^,^^51^, ^52^, ^53^^,^^56^, ^57^, ^58^, ^59^^,^^70^^,^^71^^,^^75^^,^^76^^,^^78^, ^79^, ^80^, ^81^^,^^85^^,^^86^^,^^89^^,^^90^ When including only the 41 studies without age limitations, the crude incidence was 23.9 per 100,000 person-years (95% CI 20.1–28.4).^4^, ^5^, ^6^^,^^20^^,^^25^^,^^30^^,^^31^^,^^33^^,^^34^^,^^36^^,^^38^^,^^39^^,^^43^^,^^44^^,^^51^, ^52^, ^53^, ^54^^,^^56^, ^57^, ^58^, ^59^^,^^62^^,^^65^^,^^68^^,^^70^^,^^71^^,^^73^, ^74^, ^75^, ^76^^,^^78^, ^79^, ^80^, ^81^, ^82^^,^^84^, ^85^, ^86^^,^^89^^,^^90^ When including only studies with a midyear of 2003 or later, the crude incidence was 29.1 per 100,000 person-years (95% CI 22.5–37.8).^4^, ^5^, ^6^^,^^19^^,^^21^, ^22^, ^23^, ^24^, ^25^^,^^30^, ^31^, ^32^, ^33^, ^34^^,^^36^^,^^37^^,^^39^^,^^40^^,^^42^^,^^44^^,^^47^^,^^49^^,^^52^^,^^53^^,^^55^, ^56^, ^57^^,^^59^^,^^67^, ^68^, ^69^, ^70^, ^71^, ^72^, ^73^, ^74^, ^75^, ^76^^,^^79^, ^80^, ^81^, ^82^, ^83^, ^84^^,^^86^^,^^87^^,^^89^^,^^90^

Thirty-three studies provided data on incidence for men and women separately (Table 1).^20^^,^^25^^,^^30^^,^^32^, ^33^, ^34^^,^^36^^,^^37^^,^^39^^,^^43^^,^^47^^,^^52^, ^53^, ^54^, ^55^, ^56^, ^57^, ^58^, ^59^^,^^62^^,^^65^^,^^68^^,^^70^^,^^71^^,^^73^^,^^76^^,^^78^^,^^79^^,^^84^, ^85^, ^86^^,^^89^^,^^90^ The incidence was lower in women than in men (22.8, 95% CI 17.9–29.0 in women vs 26.9, 95% CI 21.1–34.1 in men; IRR 0.88, 95% CI 0.81–0.97).

Twenty-eight studies described the incidence in one or more mid-decade age bands.^23^^,^^25^^,^^32^, ^33^, ^34^^,^^36^^,^^38^, ^39^, ^40^^,^^47^^,^^54^, ^55^, ^56^, ^57^, ^58^, ^59^^,^^62^^,^^65^^,^^68^^,^^71^^,^^73^^,^^75^^,^^76^^,^^78^^,^^79^^,^^82^^,^^84^^,^^85^ The incidence increased with age, with the lowest incidence in the <45 years age group and the highest incidence in the ≥85 age group (Table 2).

**Table 2:** Incidence of intracerebral haemorrhage according to age.

Age group	Number of studies	Patients with intracerebral haemorrhage	Number of person-years	Incidence per 100,000 person-years (95% CI)	Incidence rate ratio (95% CI)
<45 years	22^25^^,^^33^^,^^34^^,^^36^^,^^38^, ^39^, ^40^^,^^47^^,^^54^^,^^55^^,^^57^, ^58^, ^59^^,^^65^^,^^68^^,^^73^^,^^75^^,^^76^^,^^78^^,^^79^^,^^84^^,^^85^	224	7,153,160	3.0 (2.5–3.7)	0.12 (0.09–0.18)
45–54 years	21^25^^,^^33^^,^^34^^,^^36^^,^^38^, ^39^, ^40^^,^^54^^,^^55^^,^^57^, ^58^, ^59^^,^^65^^,^^68^^,^^73^^,^^75^^,^^76^^,^^78^^,^^79^^,^^84^^,^^85^	429	1,596,721	23.4 (18.2–30.2)	Reference
55–64 years	21^23^^,^^25^^,^^33^^,^^34^^,^^36^^,^^38^, ^39^, ^40^^,^^54^^,^^57^, ^58^, ^59^^,^^65^^,^^68^^,^^73^^,^^75^^,^^76^^,^^78^^,^^79^^,^^84^^,^^85^	787	1,322,698	51.1 (39.9–65.4)	2.18 (1.53–3.11)
65–74 years	22^23^^,^^25^^,^^33^^,^^34^^,^^36^^,^^38^, ^39^, ^40^^,^^54^^,^^56^, ^57^, ^58^, ^59^^,^^65^^,^^68^^,^^73^^,^^75^^,^^76^^,^^78^^,^^79^^,^^84^^,^^85^	1034	1,170,940	76.4 (62.8–93.0)	3.26 (2.37–4.50)
75–84 years	20^23^^,^^25^^,^^33^^,^^34^^,^^38^, ^39^, ^40^^,^^54^^,^^57^, ^58^, ^59^^,^^62^^,^^65^^,^^71^^,^^73^^,^^76^^,^^78^^,^^79^^,^^84^^,^^85^	1173	729,524	144.3 (123.9–168.1)	6.17 (4.59–8.29)
≥85 years	20^23^^,^^25^^,^^32^, ^33^, ^34^^,^^38^, ^39^, ^40^^,^^54^^,^^57^, ^58^, ^59^^,^^65^^,^^71^^,^^73^^,^^76^^,^^78^^,^^79^^,^^84^^,^^85^	519	230,197	220.3 (192.8–251.8)	9.41 (7.07–12.53)

Results are obtained from subgroup analyses, with the different age groups as subgroups.

The overall crude incidence was highest in lower-middle income countries,^33^^,^^72^^,^^75^^,^^90^ being 55.3 per 100,000 person-years (95% CI 18.5–165.2; 4 studies). The crude incidence was 28.9 per 100,000 person-years (95% CI 22.9–36.4; 48 studies) in high income countries,^5^^,^^6^^,^^18^, ^19^, ^20^, ^21^, ^22^, ^23^^,^^25^^,^^31^^,^^32^^,^^34^^,^^37^, ^38^, ^39^^,^^43^^,^^44^^,^^47^^,^^49^^,^^51^, ^52^, ^53^, ^54^, ^55^, ^56^, ^57^^,^^59^^,^^60^^,^^62^^,^^65^, ^66^, ^67^^,^^69^, ^70^, ^71^^,^^73^^,^^74^^,^^76^, ^77^, ^78^, ^79^, ^80^^,^^82^, ^83^, ^84^, ^85^, ^86^^,^^89^ 24.1 per 100,000 person-years (95% CI 11.0–52.7; 9 studies) in upper-middle income countries,^4^^,^^24^^,^^30^^,^^36^^,^^42^^,^^58^^,^^68^^,^^81^^,^^87^ and 21.7 per 100,000 person-years (95% CI 17.1–27.5) in the single low income country.^40^ Table 3 shows the incidences and IRRs per country income level.

**Table 3:** Incidence of intracerebral haemorrhage according to country income level.

Country income level	Number of studies	Patients with intracerebral haemorrhage	Number of person-years	Incidence per 100,000 person-years (95% CI)	Incidence rate ratio (95% CI)
High	48^5^^,^^6^^,^^18^, ^19^, ^20^, ^21^, ^22^, ^23^^,^^25^^,^^31^^,^^32^^,^^34^^,^^37^, ^38^, ^39^^,^^43^^,^^44^^,^^47^^,^^49^^,^^51^, ^52^, ^53^, ^54^, ^55^, ^56^, ^57^^,^^59^^,^^60^^,^^62^^,^^65^, ^66^, ^67^^,^^69^, ^70^, ^71^^,^^73^^,^^74^^,^^76^, ^77^, ^78^, ^79^, ^80^^,^^82^, ^83^, ^84^, ^85^, ^86^^,^^89^	8181	37,184,286	28.9 (22.9–36.4)	Reference
Upper-middle	9^4^^,^^24^^,^^30^^,^^36^^,^^42^^,^^58^^,^^68^^,^^81^^,^^87^	8691	9,638,163	24.1 (11.0–52.7)	0.83 (0.37–1.89)
Lower-middle	4^33^^,^^72^^,^^75^^,^^90^	519	1,647,752	55.3 (18.5–165.2)	1.91 (0.62–5.86)
Low	1^40^	68	313,722	21.7 (17.1–27.5)	0.75 (0.54–1.05)

Results are obtained from subgroup analyses, with the different country income levels as subgroups.

We found no trend over time in overall crude incidence (annual decrease of 1.38%, 95% CI −4.44 to 1.78). We did find a decrease over time in incidence in age group 85 years or older (annual decrease of 2.05%, 95% CI −3.57 to −0.51), but not in other age groups. Furthermore, we found no time trends in incidence according to sex or country income level (Supplementary Table S3). In 15 regions, data on incidence in multiple time periods were available.^4^, ^5^, ^6^^,^^18^^,^^19^^,^^21^^,^^22^^,^^25^^,^^31^^,^^36^^,^^43^^,^^51^^,^^54^^,^^56^^,^^68^^,^^78^^,^^79^^,^^89^^,^^90^ The IRRs and annual percent changes in the 15 regions are shown in Supplementary Table S4. A significant decrease was found in three regions, whereas a significant increase was found in two regions. The pooled IRR (0.89, 95% CI 0.72–1.12) and the pooled annual percent change (−0.86, 95% CI −2.80 to 1.07) did not show a significant change in incidence over time.

**Fig. 3:**
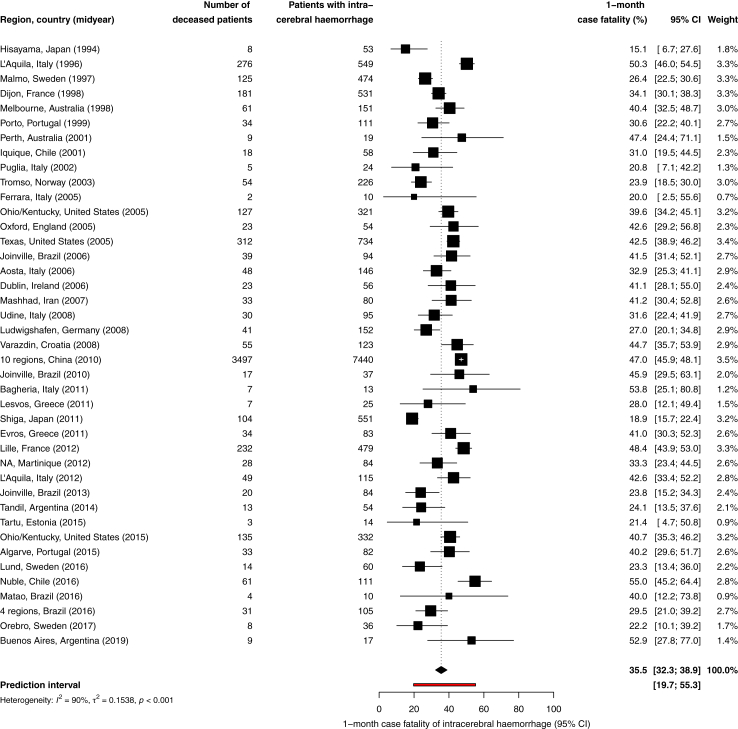
**One-month case fatality of intracerebral haemorrhage**. Size of the point estimates is proportional to the weight of the studies. The weight of the studies is determined by the inverse of the variance of the study plus the between-study variance.

Forty-one studies including 13,793 ICH patients reported on 1-month case fatality.^4^^,^^6^^,^^11^^,^^18^^,^^21^^,^^22^^,^^24^^,^^26^^,^^30^, ^31^, ^32^^,^^34^^,^^36^, ^37^, ^38^, ^39^^,^^41^^,^^45^, ^46^, ^47^^,^^51^, ^52^, ^53^^,^^55^^,^^57^^,^^58^^,^^63^^,^^64^^,^^68^^,^^71^^,^^74^^,^^76^^,^^78^^,^^79^^,^^81^^,^^82^^,^^84^, ^85^, ^86^^,^^88^^,^^89^ The overall 1-month case fatality was 35.5% (95% CI 32.3–38.9; Fig. 3). Heterogeneity among the studies was considerable (I^2^ = 90%; relatively high τ^2^ and wide prediction interval).

Eight studies reported on 1-month case fatality according to sex.^26^^,^^46^^,^^57^^,^^76^^,^^85^^,^^86^^,^^88^^,^^89^ Case fatality in women (40.1%, 95% CI 33.6–48.6) was similar to that in men (38.1%, 95% CI 29.6–47.4), with a RR of 1.07 (95% CI 0.88–1.23; Supplementary Table S5). There were six studies reporting on age-specific case fatality,^37^^,^^46^^,^^57^^,^^71^^,^^86^^,^^88^ but the reported age groups varied, which precluded further analysis.

Case fatality was lowest in high income countries at 34.8% (95% CI 31.1–38.7; 32 studies),^6^^,^^11^^,^^18^^,^^21^^,^^22^^,^^26^^,^^31^^,^^32^^,^^34^^,^^37^, ^38^, ^39^^,^^46^^,^^47^^,^^51^, ^52^, ^53^^,^^55^^,^^57^^,^^63^^,^^64^^,^^71^^,^^74^^,^^76^^,^^78^^,^^79^^,^^82^^,^^84^, ^85^, ^86^^,^^88^^,^^89^ followed by upper-middle income countries with 37.9% (95% CI 30.7–45.6; RR 1.09, 95% CI 0.87–1.37; eight studies).^4^^,^^24^^,^^30^^,^^36^^,^^41^^,^^58^^,^^68^^,^^81^ The single study from a lower-middle income country reported a case fatality of 41.3% (95% CI 31.0–52.3; RR 1.19, 95% CI 0.89–1.58).^45^ There were no studies from low income countries reporting on case fatality. Table 4 shows the case fatality per country income level.

**Table 4:** One-month case fatality and good functional outcome of intracerebral haemorrhage at 3–12 months according to country income level.

Country income level	Case fatality in percentage (95% CI)	Risk ratio (95% CI)	Good functional outcome in percentage (95% CI)	Risk ratio (95% CI)
High	34.8 (31.1–38.7)^6^^,^^11^^,^^18^^,^^21^^,^^22^^,^^26^^,^^31^^,^^32^^,^^34^^,^^37^, ^38^, ^39^^,^^46^^,^^47^^,^^51^, ^52^, ^53^^,^^55^^,^^57^^,^^63^^,^^64^^,^^71^^,^^74^^,^^76^^,^^78^^,^^79^^,^^82^^,^^84^, ^85^, ^86^^,^^88^^,^^89^	Reference	25.6 (17.4–36.0)^47^^,^^57^^,^^63^^,^^74^^,^^80^	Reference
Upper-middle	37.9 (30.7–45.6)^4^^,^^24^^,^^30^^,^^36^^,^^41^^,^^58^^,^^68^^,^^81^	1.09 (0.87–1.37)	37.0 (29.9–44.8)^4^^,^^36^^,^^41^^,^^58^^,^^81^	1.45 (0.95–2.19)
Lower-middle	41.3 (31.0–52.3)^45^	1.19 (0.89–1.58)	–	–
Low	–	–	–	–

Results are obtained from subgroup analyses, with the different country income levels as subgroups.

We found no time trend in overall case fatality (annual increase of 0.72%, 95% CI −1.55 to 3.05), nor in case fatality per country income level (Supplementary Table S3). We had insufficient data to assess time trends for case fatality per sex or age group.

**Fig. 4:**
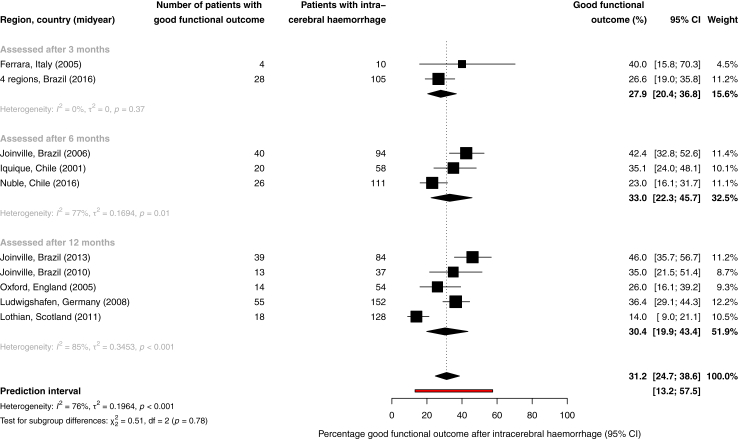
**Percentage of patients with good functional outcome after intracerebral haemorrhage**. Size of the point estimates is proportional to the weight of the studies. The weight of the studies is determined by the inverse of the variance of the study plus the between-study variance.

Ten studies reported on functional outcome.^4^^,^^36^^,^^41^^,^^47^^,^^57^^,^^58^^,^^63^^,^^74^^,^^80^^,^^81^ Functional outcome was assessed after 12 months in five studies,^4^^,^^41^^,^^63^^,^^74^^,^^80^ after six months in three,^36^^,^^57^^,^^58^ and after three months in two studies.^47^^,^^81^ All studies used the modified Rankin Scale (mRS) as outcome measure. One study defined good functional outcome as mRS 0–3,^74^ the others as mRS 0–2. Of the 833 ICH patients followed, 31.2% (95% CI 24.7–38.6) had a good functional outcome (Fig. 4). Heterogeneity among the studies was considerable (I^2^ = 76%; relatively high τ^2^ and wide prediction interval). Only one study reported on functional outcome for different sexes, reporting a mRS 0–3 in 40.6% of men and 31.7% of women.^74^ No study reported on functional outcome for different age groups. The percentage of patients with good functional outcome was 25.6% (95% CI 17.4–36.0; 5 studies) in high income countries,^47^^,^^57^^,^^63^^,^^74^^,^^80^ and 37.0% (95% CI 29.9–44.8; RR 1.45, 95% CI 0.95–2.19; 5 studies) in upper-middle income countries (Table 4).^4^^,^^36^^,^^41^^,^^58^^,^^81^ There was no information on functional outcome in lower and lower-middle income countries. We found no time trend in the percentage of patients with good functional outcome over time (annual decrease of 3.14%, 95% CI -9.56–3.74).

## Discussion

Based on this systematic review and meta-analysis of 70 population-based prospective studies, overall crude incidence of ICH remains high at 29.2 per 100,000 person-years. One-month case fatality was 35.5%, and less than one-third of patients with ICH recovered to functional independence. We noted a lower incidence in women than in men, whereas case-fatality was similar for both sexes. Information on sex differences in functional outcome is sparse. Incidence increased with increasing age. There was insufficient information to analyse the effect of age on case fatality or functional outcome. Estimates for lower and lower-middle income countries were less precise or not available, but appear unfavourable in comparison with higher income countries. We could not demonstrate significant trends over time in incidence, case fatality or functional outcome over the past 15 years.

The overall crude incidence in this systematic review was higher than in our previous review reporting on cohorts with a midyear between 1983 and 2006 (29.2 vs 24.6 per 100,000 person-years),^2^ although confidence intervals were overlapping. There are four possible explanations for these differences. First, we included more studies from lower-middle income countries, which have higher incidences. Second, the availability of neuro-imaging in stroke patients has become more widespread, as is shown by a median percentage of cases confirmed of 97.3% in this review, compared to 93.7% (IQR 89.8–97.4) in our previous one. This may have led to a higher detection rate of ICH. Third, prevalence of hypertension has risen over the past decades, especially in low and middle-income countries. Also, the proportion of hypertensive patients receiving treatment and reaching adequate blood pressure control is low.^92^ On top of that, there has been a substantial increase in the global prevalence of other risk factors for ICH, such as particulate matter pollution, obesity and diabetes mellitus.^93^, ^94^, ^95^, ^96^ Finally, with progressive ageing of the global population, there is a larger proportion of people aged 65 years or older, who are at increased risk of having an ICH.^97^

The overall crude incidence was also higher than found in a more recently published systematic review and meta-analysis of cohorts with a midyear between 1996 and 2017 (29.2 vs 26.5 per 100,000 person-years),^14^ but with overlapping confidence intervals. This may be explained by different inclusion periods and differences in methodology. To start, different search strategies were performed, resulting in almost three times as many possibly eligible studies in our review. Furthermore, we did not have language limitations or restrictions based on midyear of the study. Additionally, we only included studies in which more than 80% of cases were confirmed with imaging or autopsy. Finally, we excluded studies reporting only adjusted incidence.

In contrast to the findings in our previous systematic review, we now clearly demonstrate a higher incidence of ICH in men compared to women, probably because information on sex differences was available in more studies. The higher incidence may be explained by a higher prevalence and lower control of hypertension and other modifiable risk factors in men.^98^^,^^99^ The increase in incidence by age is similar to what we found in our previous review and is probably due to a higher prevalence of risk factors and cerebral amyloid angiopathy in older patients.^8^ Moreover, the risk of oral anticoagulation-associated ICH increases with age.^8^^,^^100^

Our findings of a relatively high incidence of ICH in lower-middle income countries, are in line with a previous systematic review.^14^ The main explanation for this finding would be the high prevalence and poor treatment of risk factors in these countries.^1^^,^^92^^,^^101^ The relatively low incidence in the single low income country (India) may be explained by competing risk bias because of the relatively high burden of non-communicable diseases in low-income countries.^102^ On top of that, the detection of ICH is lower in low income countries, illustrated by the fact that we found merely one study from a low income country in which over 80% of the stroke cases were confirmed with imaging.

We did not find a trend in overall crude incidence over time in our study period, although there was a statistically significant increasing or decreasing time trend in a minority of the individual regions. This overall stable incidence may be the result of an equilibrium between better treatment of risk factors on the one hand, and a higher prevalence of these risk factors, alongside ageing of the population, on the other hand. Also, our study period may not have been long enough to detect any trend. Previous cohort studies showed varying trends over time for different sexes or age groups.^7^^,^^8^^,^^10^ When analysing these subgroups, we found only small effect sizes, of which only the time trend in the age group 85 years or older was statistically significant. Whereas we found a stable crude incidence, the GBD Study found a decrease in age-adjusted incidence over the past decades.^1^ Using GBD data, a recent modelling framework projected that the absolute number of new ICH cases and related deaths in Europe is expected to increase over the next three decades, while age-standardized rates of ICH were projected to continue to decrease.^103^

The overall case fatality of 35.5% was lower than in our previous review (40.4%),^2^ yet consistent with a more recent review (36.3%).^15^ This apparent reduction in case fatality may be explained by improved general care for patients with ICH and the implementation of care bundles in recent years,^104^ including organised stroke care and more aggressive treatment of acute hypertension.^105^, ^106^, ^107^

The finding that only 31.2% of patients regain independence after ICH, reflects the limited treatment options for patients with ICH, and stresses the need for new acute treatments. On the other hand, our results also showed that of the patients surviving the first month, approximately half regain functional independence. This is supported by a post-hoc analysis of MISTIE-III and CLEAR-III, where 43% of patients with mRS 4–5 at 1 month recovered to mRS 0–3 after 12 months.^108^ This should encourage physicians to offer early and aggressive treatment to patients with ICH, as has recently been suggested in consensus statements.^109^^,^^110^

The main strength of our systematic review and meta-analysis is that it offers a comprehensive yet concise overview of important epidemiological measures in ICH. Second, we performed an extensive literature search without restrictions in language, enabling us to identify a large number of studies. Additionally, we set strict inclusion criteria to include only studies that allow a reliable estimate of the incidence, thereby excluding potentially unreliable sources such as ICD-codes or administrative data.^111^ This is reflected by the fact that 51% of our included studies were of high quality. Finally, by including over 19,000 ICH patients from 70 studies from 26 different countries, our main estimates are robust.

Our review also has limitations. First, the majority of cohorts were from high or upper-middle income countries from Europe, North-America or Asia. Only one cohort from a low-income country (India) was included, and there were no studies from Africa that met our inclusion criteria. Second, there was considerable heterogeneity between the studies, especially in the meta-analysis on incidence. This heterogeneity could partially be explained by the cohort's geographical region, with higher incidence rates in Asian countries. It could not be explained by midyear of the study or proportion of men in the study population, although data on the latter was available in only 30 studies. Heterogeneity could also not be explained by country income level. Residual heterogeneity may result from differences in study design, sample size, and other population characteristics. Third, we used crude incidence as our primary outcome, and subsequently studies reporting only adjusted estimates for which additional information on crude incidence could not be obtained (e.g. the South London Stroke Register) were not included in the analysis. Fourth, the overall crude incidence ranged from 3.1 to 259.1 per 100,000 person-years. Fifth, in the analysis of functional outcome after ICH, cohorts from South-America were relatively overrepresented in comparison with the analyses for incidence and case-fatality, and the timing of assessment differed. Finally, we screened more than 18,000 articles in a publication period of 15 years, but did not include literature dating from later than April 2023.

With this review, we demonstrate that the burden of ICH continues to be high. Our results illustrate the importance of effective primary prevention strategies to reduce the incidence of ICH. The high case fatality and low percentage of patients with good functional outcome stress the urgent need for effective acute treatments in ICH. Monitoring of incidence, case fatality and functional outcome of ICH should be performed to evaluate the effect of implemented strategies. Future studies should be performed worldwide, assess functional outcome, and adhere to the recommendations for standardized methods and data presentation to allow comparison.

## Contributors

CJMK conceptualised the study. AW, HDB, RD, CJMK, and FHBMS designed the study. AW and MC performed the study selection and data extraction, FHBMS resolved conflicts. AW and MC directly accessed and verified the underlying data reported in the manuscript. AW, MC, and GH performed the statistical analyses. AW designed the tables and figures. AW prepared the original draft of the paper. All authors contributed to the writing of the article. All authors had full access to all the data in the study and had final responsibility for the decision to submit for publication.

## Data sharing statement

Data from this systematic review and meta-analysis can be accessed upon reasonable request by contacting the corresponding author.

## Declaration of interests

AW is supported by the CONTRAST consortium through an unrestricted research grant. MC is supported by the Dutch Heart Foundation (2019T060, Dr. W. Stiggelbout travel allowance). HB is supported by the Netherlands Organisation for Scientific Research. RD is supported by the CONTRAST consortium for the Dutch ICH Surgery Trial (NCT05460793) through an unrestricted research grant. FS is supported by the Dutch Heart Foundation (2019T060), and the CONTRAST consortium and National Healtch Care Institute and ZonMW for the Dutch ICH Surgery Trial; FS is supported by Swedish Orphan Biovitrum AB (Sobi) for of the ACTION trial (NCT04834388). CK is principal investigator of the Dutch ICH Surgery Trial and reports grant from the Promising Care funding scheme of the National Health Care Institute and ZonMw (2021038368, for the Dutch ICH Surgery Trial), Penumbra inc. (to the CONTRAST consortium for the Dutch ICH Surgery Trial), and the Dutch Heart Foundation; CK is member of the DSMB of ENCHANTED2 (NCT04140110), MOSES (NCT03961334), OPTIMAS (NCT03759938), and ENRICH-AF (NCT03950076), and chair of the DSMB of EMITT (NCT05318612) and ELAPSE (NCT05976685); CK is participant in a consensus panel meeting organised by EMCREG-International and the ESO ICH guideline committee and ESO ICH committee. Funders had no role in the study design, data collection, data analysis, data interpretation, or writing of the report. All authors declare no competing interests.
