# Supplementary material for: Incidence, case fatality, and functional outcome of intracerebral haemorrhage, according to age, sex, and country income level: a systematic review and meta-analysis
Source: Lancet Reg Health Eur. 2024 Dec 13;49:101180. doi: 10.1016/j.lanepe.2024.101180 (PMC11699750; doi:10.1016/j.lanepe.2024.101180)
Supplement: Supplementary Figures and Tables [file mmc1.pdf]

Supplementary webappendix to:

Incidence, case fatality, and functional outcome of intracerebral haemorrhage, according to age, sex, and country income level: a systematic review and meta-analysis

Axel Wolsink, Maaike P Cliteur, Charlotte J van Asch, Hieronymus D Boogaarts, Ruben Dammers, Gerjon Hannink, Floris H B M Schreuder*, Catharina J M Klijn*

* contributed equally

Affiliations:

1. Department of Neurology, Donders Institute for Brain, Cognition and Behaviour, Radboud University Medical Centre, Geert Grooteplein Zuid 10, 6525 GA Nijmegen, the Netherlands (A Wolsink MD, M P Cliteur MD, F H B M Schreuder MD, Prof C J M Klijn MD)
2. Stichting Epilepsie Instellingen Nederland (SEIN), Dr. Denekampweg 20, 8025 BV Zwolle, The Netherlands (C J J van Asch MD)
3. Department of Neurosurgery, Radboud University Medical Centre, Geert Grooteplein Zuid 10, 6525 GA Nijmegen, the Netherlands (Prof H D Boogaarts MD)
4. Department of Neurosurgery, Erasmus Medical Centre, Erasmus MC Stroke Centre, Dr. Molewaterplein 40, 3015 GD Rotterdam, the Netherlands (R Dammers MD)
5. Department of Medical Imaging, Radboud University Medical Center, Geert Grooteplein Zuid 10, 6525 GA Nijmegen, The Netherlands (G Hannink PhD)

Table of contents

Section 1. Search strategy ... 3

Section 2. Supplementary results ... 4

Supplementary table 1: Definitions of intracerebral haemorrhage used in the studies ... 4

Supplementary table 2: Quality assessment of the studies ... 7

Supplementary table 3: Time trends in incidence and one-month case fatality according to age, sex, or country income level ... 10

Supplementary table 4: Time trends in incidence within regions ... 11

Supplementary table 5: One-month case fatality of intracerebral haemorrhage according to sex ... 12

Supplementary figure 1: Funnel plot of studies reporting on incidence of intracerebral haemorrhage ... 13

Supplementary figure 2: Funnel plot of studies reporting on case fatality of intracerebral haemorrhage ... 14

Supplementary figure 3: Funnel plot of studies reporting on functional outcome of intracerebral haemorrhage ... 15

Supplementary figure 4: Forest plot of crude incidence of intracerebral haemorrhage per 100 000 person-years, stratified by continent ... 16

Section 3. References of excluded studies after full-text screening ... 17

Section 4. Overview of studies with request for additional data ... 41

Section 1. Search strategy

PubMed syntax

("population"[MeSH Terms] OR "region"[Title/Abstract] OR "regional"[Title/Abstract] OR "population based"[Title/Abstract] OR "community based"[Title/Abstract] OR "community"[Title/Abstract] OR "stroke register*"[Title/Abstract] OR "stroke registr*"[Title/Abstract])

AND

("incidence"[MeSH Terms] OR "mortality"[MeSH Terms] OR "Glasgow Outcome Scale"[MeSH Terms] OR "incidence"[Title/Abstract] OR "fatality"[Title/Abstract] OR "mortality"[Title/Abstract] OR "trend*"[Title/Abstract] OR "outcome*"[Title/Abstract] OR "modified Rankin Scale"[Title/Abstract])

AND

("hemorrhagic stroke"[Title/Abstract] OR “haemorrhagic stroke”[Title/Abstract] OR "stroke"[Title/Abstract] OR "stroke"[MeSH Terms] OR "intracranial hemorrhage, hypertensive"[MeSH Terms] OR "cerebral hemorrhage"[MeSH Terms] OR (("intracerebral"[Title/Abstract] OR "intraparenchymal"[Title/Abstract]) AND ((("hemorrhage"[Title/Abstract] OR "haemorrhage"[Title/Abstract] OR "hemorrhage"[MeSH Terms] OR "hematoma"[MeSH Terms]) OR "haematoma"[Title/Abstract]) OR "hematoma"[Title/Abstract])))

Last searched: 18 April 2023

EMBASE syntax

(exp population/ OR region.ti,ab,kf OR regional.ti,ab,kf OR population based.ti,ab,kf OR community based.ti,ab,kf OR community.ti,ab,kf OR stroke register*.ti,ab,kf OR stroke registr*.ti,ab,kf)

AND

(exp incidence/ OR incidence.ti,ab,kf OR exp fatality/ OR exp case fatality rate/ OR fatality.ti,ab,kf OR exp mortality/ OR mortality.ti,ab,kf OR outcome.ti,ab,kf OR exp Rankin scale/ OR exp Glasgow Outcome Scale/)

AND

(hemorrhagic stroke.ti,ab,kf OR haemorrhagic stroke.ti,ab,kf OR stroke.ti,ab,kf OR exp cerebrovascular accident/ OR brain hemorrhage/ or brain ventricle hemorrhage/ or cerebellum hemorrhage/ or massive intracerebral hemorrhage/ OR brain hematoma/ OR ((intracerebral.ti,ab,kf OR intraparenchymal.ti,ab,kf) AND (hemorrhage.ti,ab,kf OR haemorrhage.ti,ab,kf OR exp hematoma/ OR haematoma.ti,ab,kf OR hematoma.ti,ab,kf)))

Last searched: 18 April 2023

Section 2. Supplementary results

Supplementary table 1: Definitions of intracerebral haemorrhage used in the studies

Author (publication year)	WHO definition for stroke ‡	ICH definition provided	Description of ICH definition applied	Percentage of cases confirmed
Aked 2018	Yes	Yes	According to Sudlow and Warlow †	99·0
Ameriso 2023	Yes	Yes	'A permanent episode of neurological dysfunction caused by a parenchymal brain haemorrhage evidenced on neuroimaging studies, regardless of duration of symptoms.'	99·1
Appelros 2019	Yes	Yes	'Patients were considered to have an IS when CT or autopsy showed no signs of haemorrhage, or when there was evidence of haemorrhagic transformation within an ischaemic territory. Other cases of intraparenchymal blood were diagnosed as ICH.'	96·7
Arnao 2021	Yes	No	No specific ICH definition provided	90·2
Azarpazhooh 2010	Yes	Yes	'A stroke for which CT or MRI demonstrated blood within the brain parenchyma, with or without extension into the ventricles or subarachnoid space. Alternatively, ICHs, SAHs, or ISs were confirmed on autopsy.'	98·7
Bahit 2016	Yes	Yes	'Neurological dysfunction caused by a parenchymal brain haemorrhage evidenced on neuroimaging studies.'	98·9
Bejot 2017	Yes	No	No specific ICH definition provided. 'ICH related to a trauma, tumor, vascular malformation, or haemorrhagic transformation of a cerebral infarct were excluded.'	100
Cabral 2009	No	Yes	CT revealed hyperdense brain areas in a topography consistent with the clinical syndrome. Excluded subdural, epidural or intracerebral haemorrhage secondary to arteriovenous malformation or tumours	93·1
Cabral 2016	Yes	No	No specific ICH definition provided	99·0
Carlsson 2016	Yes	Yes	'A parenchymal haemorrhage on CT and/or MRI and/or autopsy. ICHs caused by haemorrhagic transformation of ischemic stroke, trauma, brain surgery, hematologic disease or brain tumor were excluded.'	nr □
Chen 2020	n.r.	Yes	Neurological dysfunction caused by haemorrhage into the brain parenchyma or the ventricular system, excluding those induced by injury, with or without neuroimaging evidence of brain haemorrhage.	92·0
Correia 2013	Yes	Yes	According to Sudlow and Warlow †	96·9
Correia 2017	Yes	Yes	According to Sudlow and Warlow †	99·8
Corso 2013	Yes	Yes	According to Sudlow and Warlow †	93·4
Dalal 2008	Yes	Yes	According to WHO definition ‡	89·2
DeCampos 2017	Yes	Yes	According to the American Heart Association/American Stroke Association ±	nr □
DelBrutto 2017	Yes	No	No specific ICH definition provided	nr □
Delpont 2017	Yes	No	No specific ICH definition provided	97·4
Diaz-Guzman 2012	Yes	Yes	According to the International Classification of Diseases (9th revision) «	99·1
Farzadfard 2018	Yes	Yes	'A stroke for which CT or MRI demonstrated blood within the brain parenchyma, with or without extension into the ventricles or subarachnoid space. Alternatively, ICHs, SAHs, or ISs were confirmed on autopsy.'	97·1
Gardener 2020	Yes	No	No specific ICH definition provided	98·3
Gauthier 2021	Yes	No	No specific ICH definition provided	nr □
Gotoh 2014	Yes	No	No specific ICH definition provided	100
Graber 2020	Yes	Yes	According to WHO definition ‡	100
Groppo 2012	Yes	No	No specific ICH definition provided	100
Hata 2013	Yes	No	No specific ICH definition provided	99·0
Howard 2013	Yes	No	No specific ICH definition provided	100
Islam 2008	Yes	No	No specific ICH definition provided	89·1
Janes 2013	Yes	No	No specific ICH definition provided	97·3
Kelly 2012	Yes	Yes	'Primary intracerebral haemorrhage was verified by evidence of acute intraparenchymal haematoma on brain imaging or autopsy.'	96·1
Kita 2009	Yes	Yes	According to WHO definition ‡	93·6
Kleindorfer 2010	No	Yes	According to the Classification for Cerebrovascular Disease III º	nr □
Kolominsky-Rabas 2015	Yes	No	No specific ICH definition provided	94·9
Korv 2021	Yes	No	No specific ICH definition provided	100
Krishnamurthi 2018	Yes	Yes	According to WHO definition ‡	97·0

Lavados 2010	No	Yes	'Acute stroke in which a brain CT showed an area of high attenuation in a region compatible with the clinical signs and symptoms, or in which postmortem examination showed ICH. Excluded all cases with ICH resulting from non-vascular causes (tumour or trauma).' Excluded patients with vascular malformations from analysis.	100
Lavados 2021	No	Yes	According to the American Heart Association/American Stroke Association ±	90·0
Leyden 2013	Yes	Yes	According to WHO definition ‡<br>'Traumatic haemorrhages were also recorded but not included in reported stroke incidence to be consistent with the WHO definition.'	96·0
Li 2008	Yes	Yes	According to the International Classification of Diseases (9th revision) «	97·0
Lioutas 2020	Yes	No	No specific ICH definition provided<br>'Participants with secondary haemorrhages and those without definitive autopsy results or imaging confirmation of intraparenchymal haemorrhage were excluded'	100
Luengo-Fernandez 2013	Yes	Yes	According to WHO definition ‡	97·0
Madsen 2020	No	Yes	According to the Classification of Cerebrovascular Disease III º	nr □
Manobianca 2008	Yes	No	No specific ICH definition provided	93·7
Manobianca 2010	Yes	Yes	According to Sudlow and Warlow †	93·7
Matsumoto 2010	Yes	Yes	'Subtype classification was conducted according to the criteria of the National Institute of Neurological Disorders and Stroke.' †	98·9
Meirhaeghe 2018	Yes	No	No specific ICH definition provided	97·1
Minelli 2020	Yes	Yes	According to Sudlow and Warlow †	100
Murakami 2017	No	Yes	According to the Classification of Cerebrovascular Disease III º	97·7
Newbury 2017	Yes	No	No specific ICH definition provided	94·0
Nzwalo 2017	Yes	Yes	'ICH was diagnosed according to WHO recommendations combined with brain imaging confirmation or autopsy. ICH caused by trauma, tumors, arteriovenous malformations, cavernomas, aneurysms, hematological disorders, and haemorrhagic transformation were excluded.'	100
Olindo 2014	Yes	No	No specific ICH definition provided	99·4
Olofindayo 2015	Yes	No	No specific ICH definition provided	98·3
Omama 2013	Yes	Yes	According to WHO definition ‡	99·0
Palm 2013	Yes	No	No specific ICH definition provided. Excluded 'primary subdural/ epidural haematoma, traumatic ICH, haemorrhage due to a tumour or haemorrhagic transformation of a cerebral infarction'	100
Pandian 2016	Yes	No	No specific ICH definition provided	95·0
Pikija 2012	Yes	Yes	According to Sudlow and Warlow †	88·3
Rissanen 2019	Yes	Yes	According to the International Classification of Diseases «<br>'Traumatic SAH, traumatic ICH, epidural hematoma or subdural hematoma were not included in the series.'	86·9
Sacco 2009	Yes	Yes	'Neurological deficit documented by brain CT or MRI showing the presence of an ICH. In the absence of brain neuroimaging or necropsy examination, a diagnosis of probable ICH was made in the presence of clinical manifestations reflecting increased intracranial pressure such as headache and vomiting, decreased alertness or coma, and gradual progression to death within 24 hours of onset. Secondary causes are excluded.'	84·5
Sacco 2016	n.r.	Yes	'Neurological deficit documented by brain CT or MRI and indicating the presence of an ICH or in the absence of brain imaging or necropsy a diagnosis of probable ICH could be considered in a patient without history of occlusive peripheral vascular disease and in the presence of clinical manifestations reflecting increased intracranial pressure such as headache and vomiting, decreased alertness or coma, and gradual progression to death within 24 h of onset. Secondary ICH was excluded, thus included subjects consisted of cases of PICH attributable to hypertensive angiopathy, cerebral amyloid angiopathy or to undetermined cause.'	96·7
Samarasekera 2015	n.r.	Yes	'A symptomatic event (new headache, altered level of consciousness, or neurological symptoms), with or without new neurological signs, referable to a focal collection of blood within the brain parenchyma (seen on brain imaging or at autopsy), with signal characteristics on brain imaging or organization of the hematoma at autopsy consistent with the time of symptom onset, which was not attributable to prior trauma or haemorrhagic transformation of an ischemic stroke or an alternative explanation.'	100

Santos 2022	Yes	No	No specific ICH definition provided. Excluded 'subdural and epidural haemorrhages, whether traumatic or not; intracerebral haemorrhages secondary to rupture of arteriovenous malformation; haemorrhages secondary to bleeding by use of oral anticoagulants or by tumor bleeding.'	100
Stranjalis 2014	Yes	No	No specific ICH definition provided	92·9
Tabara 2021	Yes	Yes	According to WHO definition ‡	99·2
Takashima 2017	Yes	Yes	According to WHO definition ‡	91·6
Thrift 2009	No	Yes	'A stroke in which a CT or MRI demonstrated intraparenchymal blood with or without extension into the ventricles or subarachnoid space. Alternatively, an autopsy demonstrated the origin of the haemorrhage as the cerebral parenchyma.'	89·2
Tsivgoulis 2018	Yes	Yes	According to the American Heart Association/American Stroke Association ±	96·2
Wang 2014	Yes	No	No specific ICH definition provided	84·5
Wang 2021	Yes	No	No specific ICH definition provided	nr □
Zahuranec 2014	Yes	Yes	'Clinical symptoms and neuroimaging that demonstrated a spontaneous focal collection of blood in the parenchyma/ ventricle. ICH due to trauma, tumor, or haemorrhagic conversion of ischemic stroke or venous sinus thrombosis is excluded, whereas ICH associated with arteriovenous malformation or anticoagulation is included.'	100
Zia 2009	Yes	Yes	'CT, MRI, or autopsy shows intraparenchymal blood in the brain. Cases were excluded if the haemorrhage was considered to be secondary (eg, caused by arteriovenous malformation/ aneurysm, thrombolysis of acute myocardial infarction, haemorrhagic infarction, tumor).'	100

Abbreviations: CT = computed tomography scan , ICH = spontaneous intracerebral haemorrhage, IS = ischemic stroke, MRI = magnetic resonance imaging, nr = not reported, SAH = subarchnoid haemorrhage, SICH = spontaneous intracerebral haemorrhage, WHO = World Health Organisation.

‡ 'Rapidly developed clinical signs of focal (or global) disturbance of cerebral function, lasting more than 24 hours or leading to death, with no apparent cause other than of vascular origin.' This definition excludes cases primary cerebral tumor, cerebral metastasis, subdural haematoma, postseizure palsy, brain trauma, and TIA. Aho K, Harmsen P, Hatano S, Marquardsen J, Smirnov VE, Strasser T. Cerebrovascular disease in the community: results of a WHO collaborative study. *Bull World Health Organ* 1980; **58**(1): 113–130.

† 'WHO-defined stroke *and* CT (or MRI) scan shows primary ICH *and/or* autopsy shows primary ICH.' Sudlow CL, Warlow CP. Comparing stroke incidence worldwide: what makes studies comparable? *Stroke* 1996; **27**(3): 550-8.

± 'Rapidly developing clinical signs of neurological dysfunction attributable to a focal collection of blood within the brain parenchyma or ventricular system that is not caused by trauma.'
Sacco RL, Kasner SE, Broderick JP, et al. An updated definition of stroke for the 21st century: a statement for healthcare professionals from the American Heart Association/American Stroke Association. *Stroke* 2013; **44**(7): 2064-89.

« 'ICD-9 code 431: Haemorrhage (of): basilar, bulbar, cerebral, cerebromeningeal, cerebellar, cortical, internal capsule, intrapontine, pontine, subcortical, ventricular; Rupture of blood vessel in brain.'
World Health Organization. Manual of the International Statistical Classification of Diseases, Injuries and Causes of Death. Based on the Recommendations of the Ninth Revision Conference, 1975, and Adopted by the Twenty-ninth World Health Assembly. *Geneva* 1977.

º 'Clinical signs of a stroke with haemorrhage inside the brain parenchyma (deep, lobar, cerebellar, or brainstem). CT reliably shows intraparenchymal haemorrhage. Epidural or subdural haemorrhage are not ordinarily considered as stroke.'
Special report from the National Institute of Neurological Disorders and Stroke. Classification of cerebrovascular diseases III. *Stroke* 1990; **21**(4): 637-76.

□ Most cases confirmed, no exact numbers were provided.

Supplementary table 2: Quality assessment of the studies

	Design		Case finding methods			Population			Time frame	Definitions			Total
**Author (publication year)**	**Population-based**	**Prospective**	**Hospitalised**	**Non-hospitalised dead**	**Non-hospitalised living**	**Size**	**Age**	**Sex**	**Duration**	**Stroke definition**	**First-ever**	**80% verification**	**Total score**
Aked 2018	1	1	1	1	1	1	1	1	1	1	1	1	12
Ameriso 2023	1	1	1	1	1	0	1	1	1	1	1	1	11
Appelros 2019	1	1	1	1	1	1	1	1	1	1	1	1	12
Arnao 2021	1	1	1	1	1	0	0	1	1	1	1	1	10
Azarpazhooh 2010	1	1	1	1	1	1	1	1	1	1	1	1	12
Bahit 2016	1	1	1	1	1	1	1	1	1	1	1	1	12
Bejot 2017	1	1	1	1	1	1	1	1	1	1	1	1	12
Cabral 2009	1	1	1	1	1	1	1	1	1	1	1	1	12
Cabral 2016	1	1	1	1	1	1	1	1	1	1	1	1	12
Carlsson 2016	1	1	1	1	0	1	0	1	1	1	1	1	10
Chen 2020	1	1	1	1	0	1	0	1	1	1	1	1	10
Correia 2013	1	1	1	1	1	1	1	1	1	1	1	1	12
Correia 2017	1	1	1	1	1	1	1	1	1	1	1	1	12
Corso 2013	1	1	1	1	1	1	1	1	1	1	1	1	12
Dalal 2008	1	1	1	1	1	1	0	1	1	1	1	1	11
DeCampos 2017	1	1	1	1	1	1	1	1	1	1	1	1	12
DelBrutto 2017	1	1	1	1	0	0	0	1	1	1	1	1	9
Delpont 2017	1	1	1	1	1	1	1	1	1	1	1	1	12
Diaz-Guzman 2012	1	1	1	1	1	1	1	1	1	1	1	1	12
Farzadfard 2018	1	1	1	1	1	1	1	1	1	1	1	1	12
Gardener 2020	1	1	0	0	0	1	0	1	1	1	1	1	8
Gauthier 2021	1	1	1	1	1	1	0	1	1	1	1	1	11
Gotoh 2014	1	1	1	0	1	0	0	1	1	1	1	1	9
Graber 2020	1	1	1	1	1	1	1	1	1	1	1	1	12
Groppo 2012	1	1	1	1	1	1	0	1	1	1	1	1	11
Hata 2013	1	1	1	0	1	0	0	1	1	1	1	1	9

Howard 2013	1	1	0	0	0	1	0	1	1	1	1	1	8
Islam 2008	1	1	1	1	1	1	1	1	1	1	1	1	12
Janes 2013	1	1	1	1	1	1	1	1	1	1	1	1	12
Kelly 2012	1	1	1	1	1	1	1	1	1	1	1	1	12
Kita 2009	1	1	1	1	0	1	1	1	1	1	1	1	11
Kleindorfer 2010	1	1	1	1	1	1	0	1	1	0	1	1	10
Kolominsky-Rabas 2015	1	1	1	1	1	1	1	1	1	1	1	1	12
Korv 2021	1	1	1	1	1	1	0	1	1	1	1	1	11
Krishnamurthi 2018	1	1	1	1	1	1	1	1	1	1	1	1	12
Lavados 2010	1	1	1	1	1	1	1	1	1	1	1	1	12
Lavados 2021	1	1	1	1	1	1	1	1	1	1	1	1	12
Leyden 2013	1	1	1	1	1	1	1	1	1	1	1	1	12
Li 2008	1	1	1	0	1	1	0	1	1	1	1	1	10
Lioutas 2020	1	1	0	0	0	1	1	1	1	0	1	1	8
Luengo-Fernandez 2013	1	1	1	1	1	1	1	1	1	1	1	1	12
Madsen 2020	1	1	1	1	1	1	0	1	1	1	1	1	11
Manobianca 2008	1	1	1	1	1	0	1	1	1	1	1	1	11
Manobianca 2010	1	1	1	1	1	0	1	1	1	1	1	1	11
Matsumoto 2010	1	1	1	0	0	1	0	1	1	1	1	1	9
Meirhaeghe 2018	1	1	1	1	1	1	0	1	1	1	1	1	11
Minelli 2020	1	1	1	1	1	0	1	1	1	1	1	1	11
Murakami 2017	1	1	1	1	0	1	0	1	1	0	1	1	9
Newbury 2017	1	1	1	1	1	1	1	1	1	1	1	1	12
Nzwalo 2017	1	1	1	1	1	1	1	1	1	1	1	1	12
Olindo 2014	1	1	1	1	1	1	1	1	1	1	1	1	12
Olofindayo 2015	1	1	0	0	0	0	0	1	1	1	1	1	7
Omama 2013	1	1	1	0	1	1	1	1	1	1	1	1	11
Palm 2013	1	1	1	0	1	1	1	1	1	1	1	1	11
Pandian 2016	1	1	1	1	1	1	1	1	1	1	1	1	12
Pikija 2012	1	1	1	1	1	1	1	1	1	1	1	1	12
Rissanen 2019	1	1	0	1	0	1	0	1	1	1	1	1	9

Sacco 2009	1	1	1	1	1	1	1	1	1	1	1	1	12
Sacco 2016	1	1	1	1	1	1	1	1	1	1	1	1	12
Samarasekera 2015	1	1	1	1	1	1	1	1	1	1	1	1	12
Santos 2022	1	1	1	1	1	1	1	1	1	1	1	1	12
Stranjalis 2014	1	1	1	1	1	0	1	1	1	1	1	1	11
Tabara 2021	1	1	1	1	0	0	0	1	1	1	1	1	9
Takashima 2017	1	1	1	1	0	1	1	1	1	1	1	1	11
Thrift 2009	1	1	1	1	1	1	1	1	1	1	1	1	12
Tsivgoulis 2018	1	1	1	1	1	1	1	1	1	1	1	1	12
Wang 2014	1	1	1	1	1	1	1	1	1	1	1	1	12
Wang 2021	1	1	1	1	0	1	0	1	1	1	1	1	10
Zahuranec 2014	1	1	1	1	1	1	0	1	1	1	1	1	11
Zia 2009	1	1	1	0	1	1	1	1	1	1	1	1	11

Supplementary table 3: Time trends in incidence and one-month case fatality according to age, sex, or country income level

	Incidence		One-month case fatality	
	**Annual change (%)**	**95% CI**	**Annual change (%)**	**95% CI**
**Age**				
<44	0·30	-2·92–3·62	···	···
45-54	-1·43	-5·30–2·60	···	···
55-64	-1·93	-5·72–2·02	···	···
65-74	-2·35	-5·24–0·61	···	···
75-84	-0·78	-2·55–1·03	···	···
≥85	-2·05	-3·57 to -0·51	···	···
**Sex**				
Men	-1·37	-4·51–1·88	···	···
Women	-2·15	-5·20–1·01	···	···
**Country income level**				
High	-1·52	-4·58–1·65	0·61	-1·97–3·27
Upper-middle	···	···	···	···
Lower-middle	···	···	···	···
Low	···	···	···	···

Supplementary table 4: Time trends in incidence within regions

**Region, country**	**Study period 1**	**Incidence per 100 000 person-years (period 1)**	**Study period 2**	**Incidence per 100 000 person-years (period 2)**	**Incidence rate ratio (95% CI)**	**Annual percent change (95% CI)**
Lund, Sweden	2001-2002	19·62	2015-2016	21·71	1·11 (0·75–1·62)	0·73 (-2·04–3·49)
Örebro, Sweden	1999	35·63	2017	23·95	0·67 (0·43–1·04)	-2·18 (-4·57–0·22)
Joinville, Brazil	2005-2006	9·52	2012-2013	7·83	0·82 (0·61–1·10)	-2·76 (-6·85–1·33)
Porto, Portugal	1998-2000	45·34	2009-2011	21·03	0·46 (0·32–0·67)	-6·74 (-9·90 to -3·59)
Hisayama, Japan	1988-2001	161·32	2002-2009	107·00	0·66 (0·40–1·09)	-3·66 (-8·02–0·69)
Dijon, France	1987-2012	13·60	2013-2015	26·03	1·91 (1·57–2·33)	4·42 (3·05–5·80)
Perth, Australia	1989-1990	23·35	2000-2001	13·25	0·54 (0·31–0·96)	-5·38 (-10·27 to -0·50)
Takashima, Japan	1990-1992	51·58	1999-2001	53·54	1·04 (0·77–1·40)	0·42 (-2·94–3·77)
Ohio/Kentucky, United States	1993-1994	28·00	2005	32·00	1·14 (0·97–1·35)	1·22 (-0·29–2·73)
Auckland, New Zealand	2002-2003	18·00 †	2011-2012	16·00 †	0·89 (0·73–1·09)	-1·30 (-3·49–0·89)
Matao, Brazil	2003-2004	14·66	2015-2016	12·68	0·86 (0·37–2·03)	-1·20 (-8·26–5·85)
Martinique	1998-1999	22·91	2011-2012	22·65	0·99 (0·73–1·34)	-0·09 (-2·42–2·24)
L'Aquila, Italy	1994-1998	36·89	2011-2012	19·28	0·52 (0·43–0·64)	-3·97 (-5·18 to -2·77)
Tianjin, China	1999-2005	52·84	2006-2012	105·83	2·00 (1·44–2·79)	10·43 (5·23–15·63)
Texas, United States	2000-2002	52·10 †	2008-2010	43·00 †	0·83 (0·68–1·00)	-2·37 (-4·77–0·02)
**Overall**	···	···	···	···	**0·89 (0·72–1·12)**	**-0·86 (-2·80–1·07)**
Incidence rates are crude incidence per 100 000 person-years.<br>† Adjusted incidence per 100 000 person-years.						

Supplementary table 5: One-month case fatality of intracerebral haemorrhage according to sex

**Region, country (midyear)**	**Patients with intracerebral haemorrhage (n)**	**One-month case fatality in percentage (95% CI)**		**Risk ratio women versus men (95% CI)**
		**Men**	**Women**	
Malmö, Sweden (1997)	474	28.7 (23.5–34.6)	23.6 (18.5–29.7)	0.82 (0.58–1.17)
Melbourne, Australia (1998)	151	29.2 (19.9–40.7)	50.6 (39.7–61.4)	1.73 (1.02–2.94)
Varaždin, Croatia (2008)	123	45.2 (34.2–56.7)	44.0 (31.0–57.9)	0.97 (0.57–1.67)
Evros, Greece (2011)	83	38.8 (26.1–53.3)	42.6 (27.7–59.0)	1.10 (0.56–2.17)
Lille, France (2012)	479	46.0 (39.6–52.5)	51.0 (44.8–57.1)	1.11 (0.86–1.44)
Martinique (2012)	84	27.4 (17.5–40.2)	39.0 (22.5–58.4)	1.42 (0.65–3.13)
Ohio/Kentucky, United States (2015)	332	29.1 (22.6–36.6)	37.1 (31.2–43.4)	1.27 (0.89–1.82)
Ñuble, Chile (2016)	111	66.1 (52.8–77.2)	43.6 (31.2–56.9)	0.66 (0.40–1.10)

Supplementary figure 1: Funnel plot of studies reporting on incidence of intracerebral haemorrhage

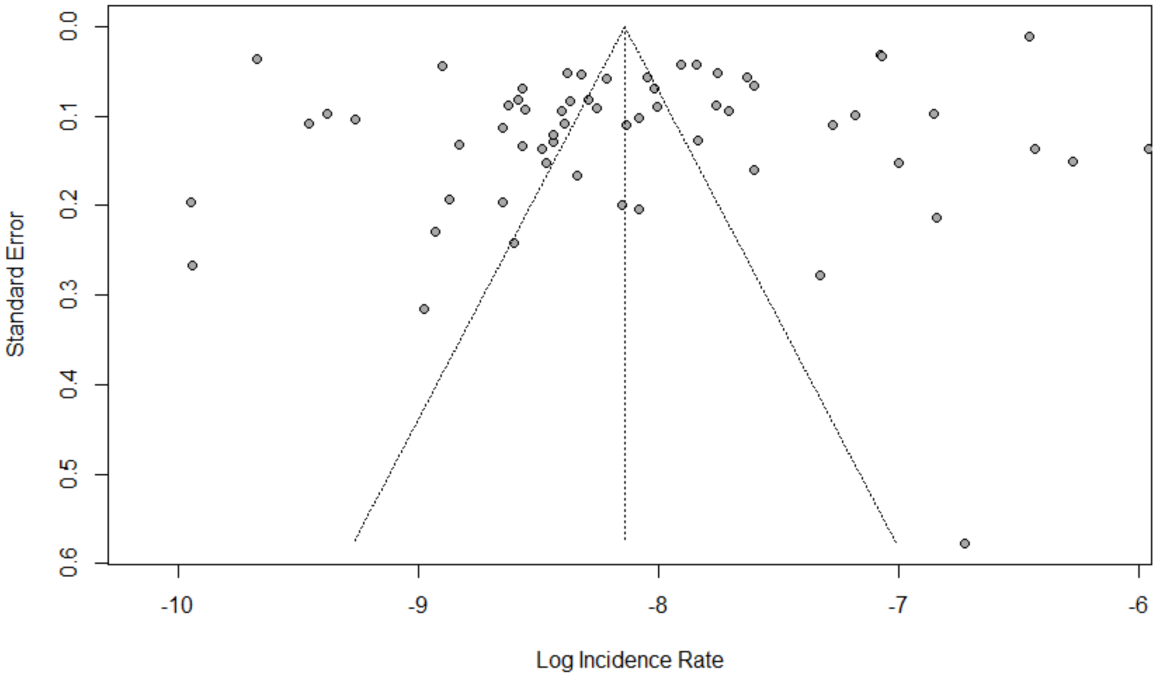

Supplementary figure 2: Funnel plot of studies reporting on case fatality of intracerebral haemorrhage

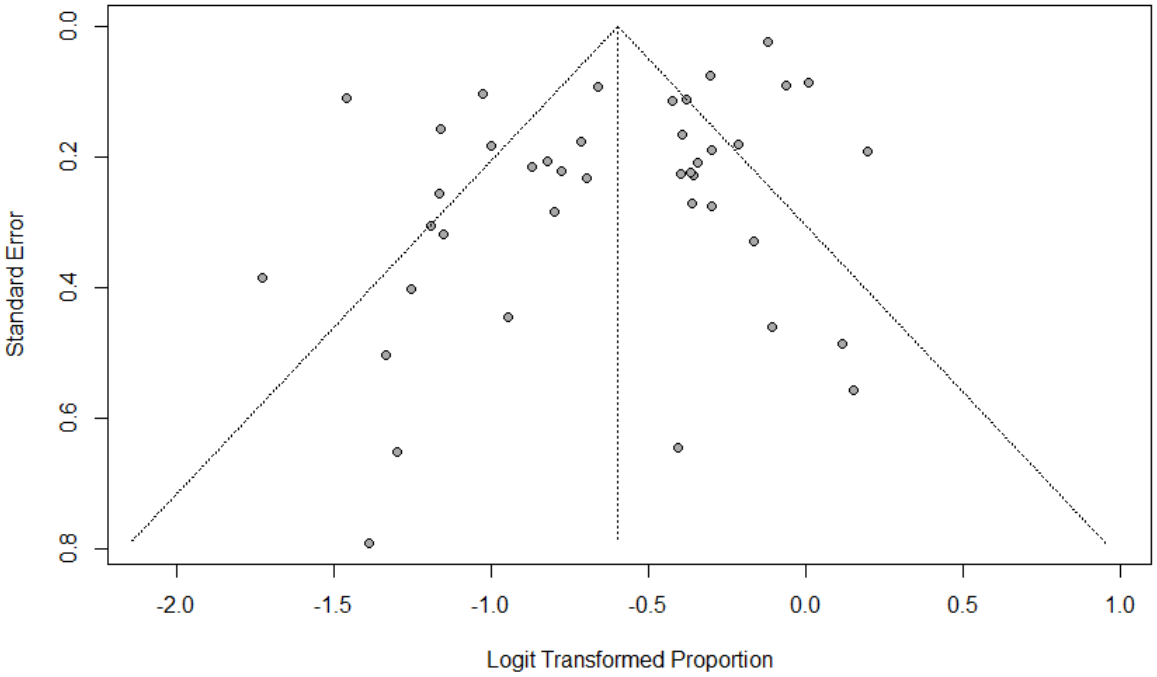

Supplementary figure 3: Funnel plot of studies reporting on functional outcome of intracerebral haemorrhage

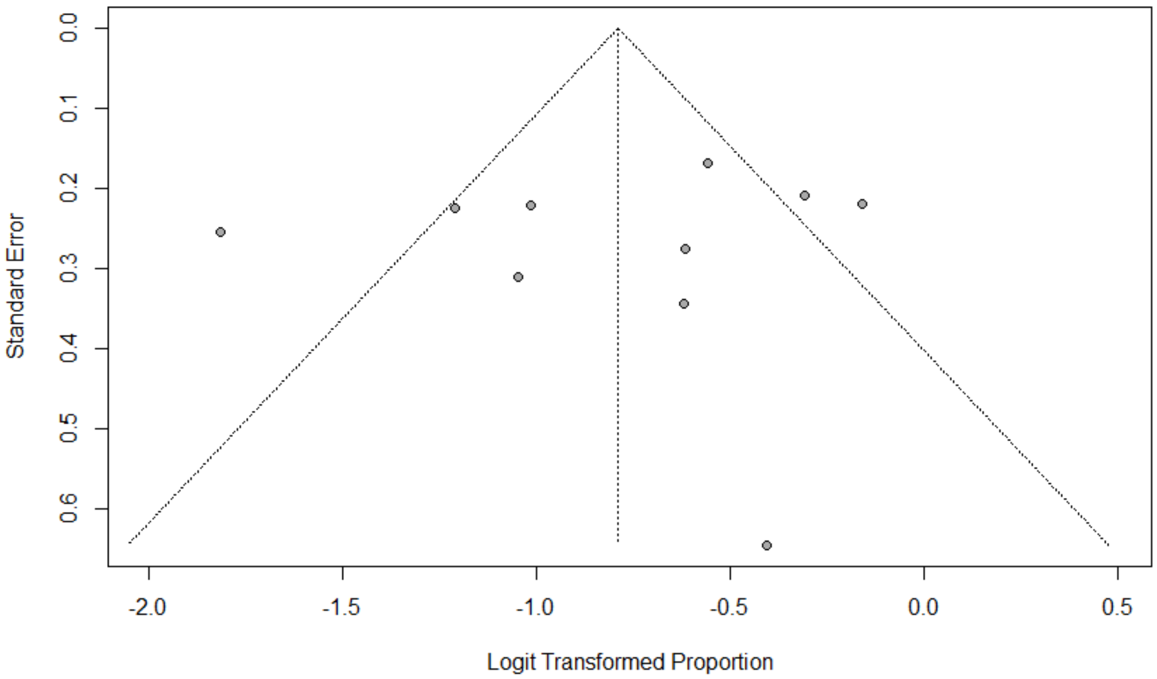

Supplementary figure 4: Forest plot of crude incidence of intracerebral haemorrhage per 100 000 person-years, stratified by continent

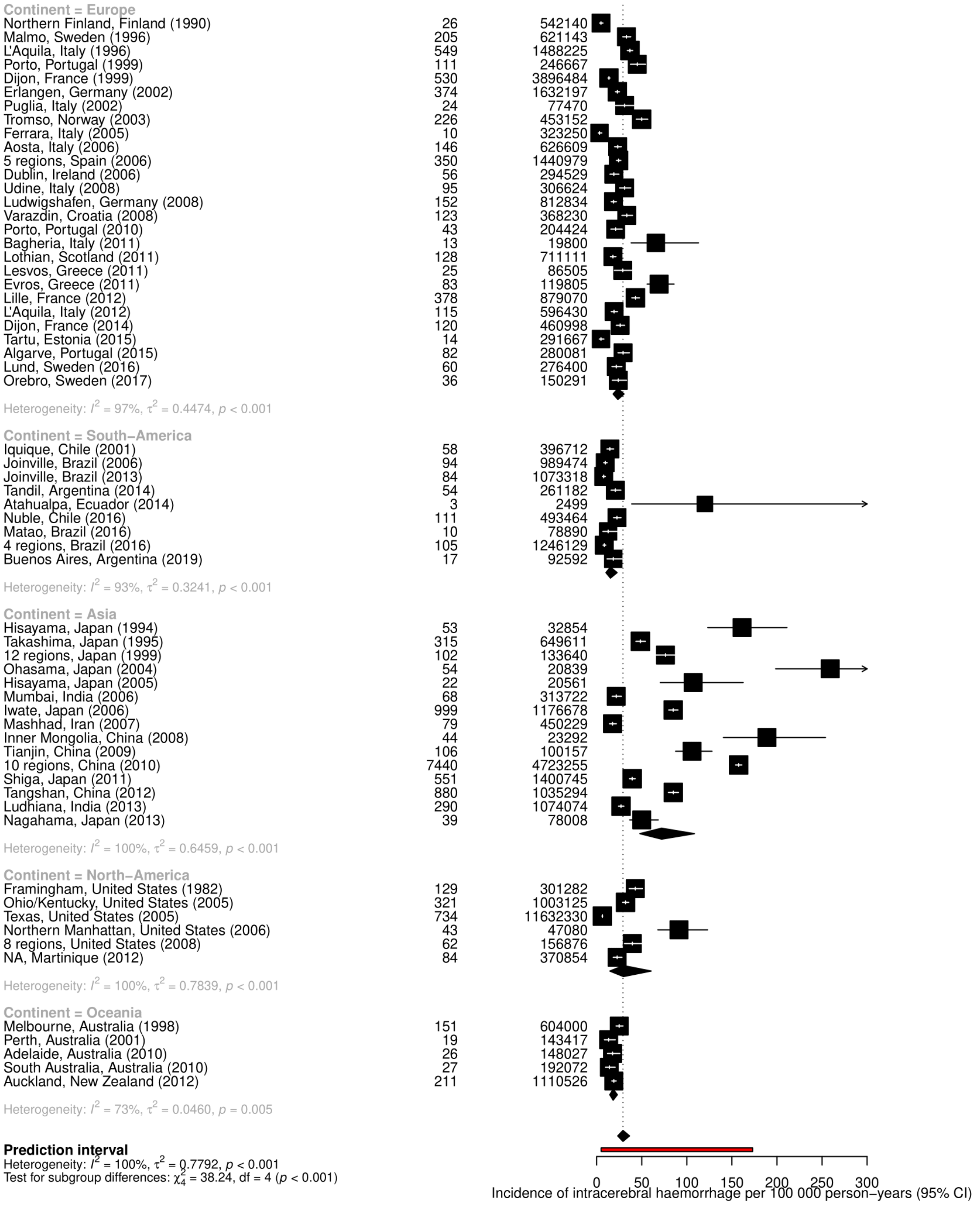

Region, country (midyear)	Patients with intra-cerebral haemorrhage	Number of person-years	Incidence per 100 000 person-years	95% CI	Weight
**Continent = Europe**					
Northern Finland, Finland (1990)	26	542140	4.80	[3.27; 7.04]	1.6%
Malmo, Sweden (1996)	205	621143	33.00	[28.78; 37.85]	1.6%
L'Aquila, Italy (1996)	549	1488225	36.89	[33.93; 40.11]	1.7%
Porto, Portugal (1999)	111	246667	45.00	[37.36; 54.20]	1.6%
Dijon, France (1999)	530	3896484	13.60	[12.49; 14.81]	1.7%
Erlangen, Germany (2002)	374	1632197	22.91	[20.71; 25.36]	1.7%
Puglia, Italy (2002)	24	77470	30.98	[20.76; 46.22]	1.6%
Tromso, Norway (2003)	226	453152	49.87	[43.78; 56.82]	1.7%
Ferrara, Italy (2005)	10	323250	3.09	[1.66; 5.75]	1.5%
Aosta, Italy (2006)	146	626609	23.30	[19.81; 27.40]	1.6%
5 regions, Spain (2006)	350	1440979	24.29	[21.87; 26.97]	1.7%
Dublin, Ireland (2006)	56	294529	19.01	[14.63; 24.71]	1.6%
Udine, Italy (2008)	95	306624	30.98	[25.34; 37.88]	1.6%
Ludwigshafen, Germany (2008)	152	812834	18.70	[15.95; 21.92]	1.6%
Varazdin, Croatia (2008)	123	368230	33.40	[27.99; 39.86]	1.6%
Porto, Portugal (2010)	43	204424	21.03	[15.60; 28.36]	1.6%
Bagheria, Italy (2011)	13	19800	65.66	[38.12; 113.07]	1.5%
Lothian, Scotland (2011)	128	711111	18.00	[15.14; 21.40]	1.6%
Lesvos, Greece (2011)	25	86505	28.90	[19.53; 42.77]	1.6%
Evros, Greece (2011)	83	119805	69.28	[55.87; 85.91]	1.6%
Lille, France (2012)	378	879070	43.00	[38.88; 47.56]	1.7%
L'Aquila, Italy (2012)	115	596430	19.28	[16.06; 23.15]	1.6%
Dijon, France (2014)	120	460998	26.03	[21.77; 31.13]	1.6%
Tartu, Estonia (2015)	14	291667	4.80	[2.84; 8.10]	1.5%
Algarve, Portugal (2015)	82	280081	29.28	[23.58; 36.35]	1.6%
Lund, Sweden (2016)	60	276400	21.71	[16.85; 27.96]	1.6%
Orebro, Sweden (2017)	36	150291	23.95	[17.28; 33.21]	1.6%
			**23.49**	**[18.15; 30.39]**	**43.7%**
Heterogeneity: $I^2 = 97\%$, $\tau^2 = 0.4474$, $p < 0.001$					
**Continent = South−America**					
Iquique, Chile (2001)	58	396712	14.62	[11.30; 18.91]	1.6%
Joinville, Brazil (2006)	94	989474	9.50	[7.76; 11.63]	1.6%
Joinville, Brazil (2013)	84	1073318	7.83	[6.32; 9.69]	1.6%
Tandil, Argentina (2014)	54	261182	20.68	[15.83; 27.00]	1.6%
Atahualpa, Ecuador (2014)	3	2499	120.05	[38.72; 372.22]	1.2%
Nuble, Chile (2016)	111	493464	22.49	[18.68; 27.09]	1.6%
Matao, Brazil (2016)	10	78890	12.68	[6.82; 23.56]	1.5%
4 regions, Brazil (2016)	105	1246129	8.43	[6.96; 10.20]	1.6%
Buenos Aires, Argentina (2019)	17	92592	18.36	[11.41; 29.53]	1.5%
			**15.21**	**[10.22; 22.64]**	**14.0%**
Heterogeneity: $I^2 = 93\%$, $\tau^2 = 0.3241$, $p < 0.001$					
**Continent = Asia**					
Hisayama, Japan (1994)	53	32854	161.32	[123.24; 211.16]	1.6%
Takashima, Japan (1995)	315	649611	48.49	[43.42; 54.15]	1.7%
12 regions, Japan (1999)	102	133640	76.32	[62.86; 92.67]	1.6%
Ohasama, Japan (2004)	54	20839	259.13	[198.46; 338.34]	1.6%
Hisayama, Japan (2005)	22	20561	107.00	[70.45; 162.50]	1.6%
Mumbai, India (2006)	68	313722	21.68	[17.09; 27.49]	1.6%
Iwate, Japan (2006)	999	1176678	84.90	[79.80; 90.33]	1.7%
Mashhad, Iran (2007)	79	450229	17.55	[14.07; 21.88]	1.6%
Inner Mongolia, China (2008)	44	23292	188.91	[140.58; 253.85]	1.6%
Tianjin, China (2009)	106	100157	105.83	[87.49; 128.03]	1.6%
10 regions, China (2010)	7440	4723255	157.52	[153.98; 161.14]	1.7%
Shiga, Japan (2011)	551	1400745	39.34	[36.19; 42.76]	1.7%
Tangshan, China (2012)	880	1035294	85.00	[79.57; 90.81]	1.7%
Ludhiana, India (2013)	290	1074074	27.00	[24.06; 30.29]	1.7%
Nagahama, Japan (2013)	39	78008	49.99	[36.53; 68.43]	1.6%
			**72.00**	**[47.75; 108.55]**	**24.5%**
Heterogeneity: $I^2 = 100\%$, $\tau^2 = 0.6459$, $p < 0.001$					
**Continent = North−America**					
Framingham, United States (1982)	129	301282	42.82	[36.03; 50.88]	1.6%
Ohio/Kentucky, United States (2005)	321	1003125	32.00	[28.68; 35.70]	1.7%
Texas, United States (2005)	734	11632330	6.31	[5.87; 6.78]	1.7%
Northern Manhattan, United States (2006)	43	47080	91.33	[67.74; 123.15]	1.6%
8 regions, United States (2008)	62	156876	39.52	[30.81; 50.69]	1.6%
NA, Martinique (2012)	84	370854	22.65	[18.29; 28.05]	1.6%
			**29.67**	**[14.54; 60.54]**	**9.8%**
Heterogeneity: $I^2 = 100\%$, $\tau^2 = 0.7839$, $p < 0.001$					
**Continent = Oceania**					
Melbourne, Australia (1998)	151	604000	25.00	[21.31; 29.32]	1.6%
Perth, Australia (2001)	19	143417	13.25	[8.45; 20.77]	1.6%
Adelaide, Australia (2010)	26	148027	17.56	[11.96; 25.80]	1.6%
South Australia, Australia (2010)	27	192072	14.06	[9.64; 20.50]	1.6%
Auckland, New Zealand (2012)	211	1110526	19.00	[16.60; 21.74]	1.6%
			**18.23**	**[14.49; 22.94]**	**8.0%**
Heterogeneity: $I^2 = 73\%$, $\tau^2 = 0.0460$, $p = 0.005$					
			**29.16**	**[23.34; 36.45]**	**100.0%**
**Prediction interval**				**[4.92; 173.00]**	
Heterogeneity: $I^2 = 100\%$, $\tau^2 = 0.7792$, $p < 0.001$					
Test for subgroup differences: $\chi^2_4 = 38.24$, df = 4 ($p < 0.001$)					

Size of the point estimates is proportional to the weight of the studies. The weight of the studies is determined by the inverse of the variance of the study plus the between-study variance.

Section 3. References of excluded studies after full-text screening

i. Not population-based (n = 146)

1. Adami F, Dos Santos Figueiredo FW, Da Silva Paiva L, et al. Mortality and incidence of hospital admissions for stroke among Brazilians aged 15 to 49 years between 2008 and 2012. *PLoS ONE* 2016; **11(6) (no pagination)**.
2. Aguilar-Salas E, Rodriguez-Aquino G, Garcia-Dominguez K, et al. Acute Stroke Care in Mexico City: The Hospital Phase of a Stroke Surveillance Study. *Brain Sciences* 2022; **12(7) (no pagination)**.
3. Al-Khaled M, Eggers J. Prognosis of intracerebral hemorrhage after conservative treatment. *Journal of Stroke and Cerebrovascular Diseases* 2014; **23(2)**: 230-4.
4. Alhazzani AA, Mahfouz AA, Abolyazid AY, et al. Study of stroke incidence in the aseer region, southwestern Saudi Arabia. *International Journal of Environmental Research and Public Health* 2018; **15(2) (no pagination)**.
5. Alhazzani AA, Mahfouz AA, Abolyazid AY, et al. In hospital stroke mortality: Rates and determinants in southwestern Saudi Arabia. *International Journal of Environmental Research and Public Health* 2018; **15(5) (no pagination)**.
6. Alkali NH, Bwala SA, Akano AO, Osi-Ogbu O, Alabi P, Ayeni OA. Stroke risk factors, subtypes, and 30-day case fatality in Abuja, Nigeria. *Niger Med J* 2013; **54**(2): 129-35.
7. Anderlini D, Wallis G, Marinovic W. Stroke hospital admission rates in Brisbane and Queensland in 2015: Data from 11,072 cases. *International Journal of Stroke* 2019; **14(4)**: 417-21.
8. Apostolaki-Hansson T, Ullberg T, Pihlsgard M, Norrving B, Petersson J. Prognosis of Intracerebral Hemorrhage Related to Antithrombotic Use: An Observational Study from the Swedish Stroke Register (Riksstroke). *Stroke* 2021: 966-74.
9. Appelros P, Jonsson F, Asberg S, et al. Trends in stroke treatment and outcome between 1995 and 2010: Observations from riks-stroke, the swedish stroke register. *Cerebrovascular Diseases* 2014; **37(1)**: 22-9.
10. Appelros P, Jonsson F, Asplund K, et al. Trends in baseline patient characteristics during the years 1995-2008: Observations from riks-stroke, the swedish stroke register. *Cerebrovascular Diseases* 2010; **30(2)**: 114-9.
11. Arauz A, Serrano F, Ameriso SF, et al. Sex Differences Among Participants in the Latin American Stroke Registry. *Journal of the American Heart Association* 2020; **9(4) (no pagination)**.
12. Arboix A, Cendros V, Besa M, et al. Trends in risk factors, stroke subtypes and outcome: Nineteen-year data from the Sagrat Cor Hospital of Barcelona Stroke Registry. *Cerebrovascular Diseases* 2008; **26(5)**: 509-16.
13. Asayama K, Ohkubo T, Yoshida S, et al. Stroke risk and antihypertensive drug treatment in the general population: the Japan arteriosclerosis longitudinal study. *J Hypertens* 2009; **27**(2): 357-64.
14. Aziz ZA, Lee YYL, Ngah BA, et al. Acute Stroke Registry Malaysia, 2010-2014: Results from the National Neurology Registry. *Journal of Stroke and Cerebrovascular Diseases* 2015; **24(12)**: 2701-9.
15. Bako AT, Pan A, Potter T, et al. Contemporary Trends in the Nationwide Incidence of Primary Intracerebral Hemorrhage. *Stroke* 2022; **29(2)**: E70-E4.
16. Balabanski AH, Goldsmith K, Giarola B, et al. Stroke incidence and subtypes in Aboriginal people in remote Australia: A healthcare network population-based study. *BMJ Open* 2020; **10(10) (no pagination)**.
17. Ban L, Abdul Sultan A, Stephansson O, et al. The incidence of first stroke in and around pregnancy: A population-based cohort study from Sweden. *European Stroke Journal* 2017; **2(3)**: 250-6.
18. Barker-Collo S, Bennett DA, Krishnamurthi RV, et al. Sex Differences in Stroke Incidence, Prevalence, Mortality and Disability-Adjusted Life Years: Results from the Global Burden of Disease Study 2013. *Neuroepidemiology* 2015; **45**(3): 203-14.
19. Batista A, Osorio R, Varela A, et al. Prediction of short-term prognosis in elderly patients with spontaneous intracerebral hemorrhage. *European Geriatric Medicine* 2021; **12(6)**: 1267-73.
20. Bejot Y, Guilloteau A, Joux J, et al. Social deprivation and stroke severity on admission: a French cohort study in Burgundy and the West Indies - Guyana region. *European Journal of Neurology* 2017; **24(5)**: 694-702.
21. Bezzaoucha A, Bouamra A, Zeddam F, et al. Stroke in the Blida region (Algeria) in 2018: Incidence and mortality according to a population-based register. *Tunis Med* 2020; **98**(1): 8-16.
22. Boe NJ, Hald SM, Jensen MM, et al. Association Between Statin Use and Intracerebral Hemorrhage Location: A Nested Case-Control Registry Study. *Neurology* 2023; **100**(10): e1048-e61.
23. Broberg E, Hjalmarsson C, Setalani M, Milenkoski R, Andersson B. Sex Differences in Treatment and Prognosis of Acute Intracerebral Hemorrhage. *Journal of Women's Health* 2023; **32(1)**: 102-8.
24. Cantu-Brito C, Majersik JJ, Sanchez BN, et al. Hospitalized stroke surveillance in the community of Durango, Mexico: The brain attack surveillance in Durango study. *Stroke* 2010; **41(5)**: 878-84.
25. Cantu-Brito C, Ruiz-Sandoval JL, Chiquete E, et al. Risk factors, causes, and outcome in stroke types in Mexico. The RENAMEVASC Study. [Spanish]. *Revista Mexicana de Neurociencia* 2011; **12(5)**: 224-34.
26. Cao Z, Liu X, Li Z, et al. Body mass index and clinical outcomes in patients with intracerebral haemorrhage: Results from the China Stroke Center Alliance. *Stroke and Vascular Neurology* 2021; **6(3)**: 424-32.

27. Chen XW, Shafei MN, Aziz ZA, Sidek NN, Musa KI. Trends in stroke outcomes at hospital discharge in first-ever stroke patients: Observations from the Malaysia National Stroke Registry (2009-2017). *Journal of the Neurological Sciences* 2019; **401**: 130-5.
28. Christensen DH, Horvath-Puho E, Schmidt M, et al. The impact of preadmission oral bisphosphonate use on 30-day mortality following stroke: A population-based cohort study of 100,043 patients. *Clinical Epidemiology* 2015; **7**: 381-9.
29. Cisse FA, Damien C, Haba M, et al. Stroke burden in Guinea: Results from the Conakry Ignace Deen Hospital stroke registry. *International Journal of Stroke* 2020; **15(6)**: 666-7.
30. Cisse FA, Ligot N, Conde K, et al. Predictors of stroke favorable functional outcome in Guinea, results from the Conakry stroke registry. *Scientific reports* 2022; **12(1)**: 1125.
31. Corraini P, Szepligeti SK, Henderson VW, Ording AG, Horvath-Puho E, Sorensen HT. Comorbidity and the increased mortality after hospitalization for stroke: a population-based cohort study. *Journal of Thrombosis and Haemostasis* 2018; **16(2)**: 242-52.
32. Craen A, Mangal R, Stead TG, Ganti L. Gender Differences in Outcomes after Non-traumatic Intracerebral Hemorrhage. *Cureus* 2019; **11**(10): e5818.
33. Dandapat S, Siddiqui FM, Fonarow GC, et al. A paradoxical relationship between hemoglobin A1C and in-hospital mortality in intracerebral hemorrhage patients. *Heliyon* 2019; **5**(5): e01659.
34. Darehed D, Blom M, Glader EL, Niklasson J, Norrving B, Eriksson M. Time Trends and Monthly Variation in Swedish Acute Stroke Care. *Front Neurol* 2019; **10**: 1177.
35. Dasch B, Berger K, Lenz P, Brunssen A. Frequency of discharge of hospitalized patients with stroke to free-standing hospice facilities-a register study from Germany. *Annals of palliative medicine* 2022; **11(10)**: 3102-22.
36. Del Brutto OH, Del Brutto VJ. Is the rate of cerebral hemorrhages declining among stroke patients in South America? *International Journal of Stroke* 2014; **9(2)**: 207-9.
37. Delbari A, Salman Roghani R, Tabatabaei SS, Rahgozar M, Lokk J. Stroke epidemiology and one-month fatality among an urban population in Iran. *Int J Stroke* 2011; **6**(3): 195-200.
38. Deljavan R, Farhoudi M, Sadeghi-Bazargani H. Stroke in-hospital survival and its predictors: The first results from Tabriz stroke registry of Iran. *International Journal of General Medicine* 2018; **11**: 233-40.
39. Dokova KG, Feigin VL. Trends in Stroke Burden in Central and Eastern Europe from 1990-2019. *Neuroepidemiology* 2022; **56(5)**: 333-44.
40. Drescher C, Buchwald F, Ullberg T, Pihlsgard M, Norrving B, Petersson J. Epidemiology of First and Recurrent Ischemic Stroke in Sweden 2010-2019: A Riksstroke Study. *Neuroepidemiology* 2023; **56(6)**: 433-42.
41. Edwards JD, Kapral MK, Fang J, Swartz RH. Long-term morbidity and mortality in patients without early complications afer stroke or transient ischemic attack. *Cmaj* 2017; **189(29)**: E954-E61.
42. Eriksson M, Asberg S, Sunnerhagen KS, Von Euler M. Sex Differences in Stroke Care and Outcome 2005-2018: Observations from the Swedish Stroke Register. *Stroke* 2021: 3233-42.
43. Eriksson M, Glader EL, Norrving B, Terent A, Stegmayr B. Sex differences in stroke care and outcome in the swedish national quality register for stroke care. *Stroke* 2009; **40(3)**: 909-14.
44. Eriksson M, Grundberg A, Inge E, von Euler M. Stroke Recurrence Following 28 Days After First Stroke in Men and Women 2012 to 2020: Observations From the Swedish Stroke Register. *J Am Heart Assoc* 2023; **12**(3): e028222.
45. Eriksson M, Norrving B, Terent A, Stegmayr B. Functional outcome 3 months after stroke predicts long-term survival. *Cerebrovascular Diseases* 2008; **25(5)**: 423-9.
46. Eshak ES, Iso H, Kokubo Y, et al. Soft drink intake in relation to incident ischemic heart disease, stroke, and stroke subtypes in Japanese men and women: The Japan Public Health Centre-based study cohort I. *American Journal of Clinical Nutrition* 2012; **96(6)**: 1390-7.
47. Fernandes TG, Goulart AC, Campos TF, et al. Early stroke case-fatality rates in three hospital registries in the northeast and southeast of Brazil. *Arquivos de Neuro-Psiquiatria* 2012; **70(11)**: 869-73.
48. Gadhia R, McCane D, Lee J, Ling KC, Jiang K, Chiu D. The HOPES Registry - Houston Methodist Hospital Outcomes-based Prospective Endpoints in Stroke. *Journal of Stroke and Cerebrovascular Diseases* 2018; **27(11)**: 2973-6.
49. Gierlotka M, Labuz-Roszak B, Wojtyniak B, et al. Early and One-Year Outcomes of Acute Stroke in the Industrial Region of Poland during the Decade 2006-2015: The Silesian Stroke Registry. *Neuroepidemiology* 2018; **50(3-4)**: 183-94.
50. Givi M, Badihian N, Taheri M, Habibabadi RR, Saadatnia M, Sarrafzadegan N. One-year survival and prognostic factors for survival among stroke patients: The PROVE-stroke study. *Journal of Research in Medical Sciences* 2022; **27(1)**: 82.
51. Goeldlin MB, Mueller A, Siepen BM, et al. Etiology, 3-Month Functional Outcome and Recurrent Events in Non-Traumatic Intracerebral Hemorrhage. *Journal of Stroke* 2022; **24(2)**: 266-77.

52. Goulart AC, Fernandes TG, Santos IS, Alencar AP, Bensenor IM, Lotufo PA. Predictors of long-term survival among first-ever ischemic and hemorrhagic stroke in a Brazilian stroke cohort. *BMC Neurology* 2013; **13 (no pagination)**.
53. Grau AJ, Eicke M, Burmeister C, Hardt R, Schmitt E, Dienlin S. Follow-up 90 Days after Stroke and TIA in the Stroke Quality Monitoring Project Rheinland-Pfalz. [German]. *Aktuelle Neurologie* 2018; **45(9)**: 636-45.
54. Grundtvig J, Ovesen C, Havsteen I, et al. Trends in incidence of oral anticoagulant-related intracerebral hemorrhage and sales of oral anticoagulants in Capital Region of Denmark 2010-2017. *European Stroke Journal* 2021; **6(2)**: 143-50.
55. Guasch-Jimenez M, Prats-Sanchez L, Martinez-Domeno A, et al. Patterns of Admission and Outcomes for Patients with Intracranial Hemorrhage in Catalonia, Spain. *World Neurosurgery* 2021; **149**: e1123-e7.
56. Hald SM, Moller S, Garcia Rodriguez LA, et al. Trends in Incidence of Intracerebral Hemorrhage and Association with Antithrombotic Drug Use in Denmark, 2005-2018. *JAMA Network Open* 2021; **4(5) (no pagination)**.
57. Hamamci M. Stroke incidence and demographic properties of patients in Ardahan province. *Turk Noroloji Dergisi* 2019; **25(3)**: 129-34.
58. Han L, Tang T, Lin Y, et al. The Disease Burden of Primary Intracerebral Hemorrhage in Hunan Province, China in 2018. *Journal of Epidemiology and Global Health* 2022; **12(3)**: 267-73.
59. Harmsen P, Wilhelmsen L, Jacobsson A. Stroke incidence and mortality rates 1987 to 2006 related to secular trends of cardiovascular risk factors in Gothenburg, Sweden. *Stroke* 2009; **40(8)**: 2691-7.
60. Henriksson KM, Farahmand B, Asberg S, Edvardsson N, Terent A. Comparison of cardiovascular risk factors and survival in patients with ischemic or hemorrhagic stroke. *International Journal of Stroke* 2012; **7(4)**: 276-81.
61. Hosomi N, Naya T, Ohkita H, et al. Predictors of intracerebral hemorrhage severity and its outcome in Japanese stroke patients. *Cerebrovascular Diseases* 2009; **27(1)**: 67-74.
62. Iacoviello L, Costanzo S, Persichillo M, et al. Hospital-based register of stroke in the Molise Region: focus on main subtypes of stroke. Years 2009-2013. *Neurological Sciences* 2016; **37(2)**: 191-8.
63. Iguchi Y, Kimura K, Sone K, et al. Stroke incidence and usage rate of thrombolysis in A Japanese Urban City: The kurashiki stroke registry. *Journal of Stroke and Cerebrovascular Diseases* 2013; **22(4)**: 349-57.
64. Ji R, Shen H, Pan Y, et al. A novel risk score to predict 1-year functional outcome after intracerebral hemorrhage and comparison with existing scores. *Critical Care* 2013; **17(6) (no pagination)**.
65. Jimenez-Conde J, Ois A, Gomis M, et al. Weather as a trigger of stroke. Daily meteorological factors and incidence of stroke subtypes. *Cerebrovasc Dis* 2008; **26**(4): 348-54.
66. Johnsen SP, Ingeman A, Hundborg HH, Schaarup SZ, Gyllenborg J. The Danish stroke registry. *Clinical Epidemiology* 2016; **8**: 697-702.
67. Johnson LS, Mattsson N, Sajadieh A, Wollmer P, Soderholm M. Serum potassium is positively associated with stroke and mortality in the large, population-based Malmo preventive project cohort. *Stroke* 2017; **48(11)**: 2973-8.
68. Joundi RA, Smith EE, Yu AYX, Rashid M, Fang J, Kapral MK. Temporal and Age-Specific Trends in Acute Stroke Incidence: A 15-Year Population-Based Study of Administrative Data in Ontario, Canada. *Canadian Journal of Neurological Sciences* 2021; **48(5)**: 685-9.
69. Kadojic D, Demarin V, Dikanovic M, et al. Incidence of Stroke and Transient Ischemic Attack in Croatia: A Population Based Study. *Collegium antropologicum* 2015; **39(3)**: 723-7.
70. Kaduka L, Muniu E, Oduor C, et al. Stroke Mortality in Kenya's Public Tertiary Hospitals: A Prospective Facility-Based Study. *Cerebrovascular Diseases Extra* 2018; **8(2)**: 70-9.
71. Kamal N, Lindsay MP, Cote R, Fang J, Kapral MK, Hill MD. Ten-year trends in stroke admissions and outcomes in Canada. *The Canadian journal of neurological sciences* 2015; **Le journal canadien des sciences neurologiques. 42(3)**: 168-75.
72. Karelis G, Micule M, Klavina E, et al. The Riga East University Hospital Stroke Registry-An Analysis of 4915 Consecutive Patients with Acute Stroke. *Medicina* 2021; **57**(6).
73. Khan K, Tanaka-Mizuno S, Turin TC, et al. Relationship of Ambient Temperature Parameters to Stroke Incidence in a Japanese Population - Takashima Stroke Registry, Japan, 1988-2010. *Circ J* 2021; **85**(12): 2215-21.
74. Koifman J, Hall R, Li S, et al. The association between rural residence and stroke care and outcomes. *Journal of the Neurological Sciences* 2016; **363**: 16-20.
75. Korneeva NN, Kirpicheva SV, Spirin NN, Pizova NV. [Comparative epidemiology of stroke in Kostroma and Yaroslavl]. *Zh Nevrol Psikhiatr Im S S Korsakova* 2010; **110**(9 Pt 2): 42-5.
76. Koton S, Telman G, Kimiagar I, Tanne D. Gender differences in characteristics, management and outcome at discharge and three months after stroke in a national acute stroke registry. *International Journal of Cardiology* 2013; **168(4)**: 4081-4.

77. Kozera G, Chwojnicki K, Sobolewski P, et al. Epidemiology and treatment of stroke in Pomeranian Province and Swietokrzyskie Province - Data from Pomeranian Stroke Registry. *Udar Mozgu - Problemy Interdyscyplinarne* 2010; **12(1-2)**: 1-9.
78. Kurtz P, Bastos LSL, Aguilar S, Hamacher S, Bozza FA. Effect of seasonal and temperature variation on hospitalizations for stroke over a 10-year period in Brazil. *International Journal of Stroke* 2021; **16(4)**: 406-10.
79. Lahti AM, Natynki M, Huhtakangas J, et al. Long-term survival after primary intracerebral hemorrhage: a population-based case-control study spanning a quarter of a century. *European journal of neurology* 2021; **22**.
80. Lallukka T, Ervasti J, Lundström E, et al. Trends in Diagnosis-Specific Work Disability Before and After Stroke: A Longitudinal Population-Based Study in Sweden. *J Am Heart Assoc* 2018; **7**(1).
81. Langagergaard V, Palnum KH, Mehnert F, et al. Socioeconomic differences in quality of care and clinical outcome after stroke: A nationwide population-based study. *Stroke* 2011; **42(10)**: 2896-902.
82. Lauretani F, Saccavini M, Zaccaria B, et al. Rehabilitation in patients affected by different types of stroke. A one-year follow-up study. *European journal of physical and rehabilitation medicine* 2010; **46(4)**: 511-6.
83. Li Z, Zhao X, Liu L, et al. Association between seizures and outcomes among intracerebral hemorrhage patients: The china national stroke registry. *Journal of Stroke and Cerebrovascular Diseases* 2015; **24(2)**: 455-64.
84. Lindmark A, Glader EL, Asplund K, Norrving B, Eriksson M. Socioeconomic disparities in stroke case fatality--Observations from Riks-Stroke, the Swedish stroke register. *Int J Stroke* 2014; **9**(4): 429-36.
85. Liu Q, Zhao W, Zou X, Xing Y, Zhou G, Li X. Sex differences in outcomes after spontaneous intracerebral hemorrhage among patients with low total cholesterol levels. *The American journal of the medical sciences* 2021; **13**.
86. Lopponen P, Qian C, Tetri S, et al. Predictive value of C-reactive protein for the outcome after primary intracerebral hemorrhage. *Journal of Neurosurgery* 2014; **121(6)**: 1374-9.
87. Lopponen P, Tetri S, Juvela S, et al. A population based study of outcomes after evacuation of primary supratentorial intracerebral hemorrhage. *Clinical Neurology and Neurosurgery* 2013; **115(8)**: 1350-5.
88. Lorking N, Wood AD, Tiamkao S, et al. Seasonality of stroke: Winter admissions and mortality excess: A Thailand National Stroke population database study. *Clinical Neurology and Neurosurgery* 2020; **199 (no pagination)**.
89. Marsden DL, Spratt NJ, Walker R, et al. Trends in stroke attack rates and case fatality in the Hunter Region, Australia 1996-2008. *Cerebrovascular Diseases* 2010; **30(5)**: 500-7.
90. Meretoja A, Kaste M, Roine RO, et al. Trends in treatment and outcome of stroke patients in Finland from 1999 to 2007. PERFECT Stroke, a nationwide register study. *Annals of Medicine* 2011; **43(SUPPL. 1)**: S22-S30.
91. Moradi S, Moradi G, Piroozi B. The Burden of Stroke in Kurdistan Province, Iran from 2011 to 2017. *Journal of Preventive Medicine and Public Health* 2021; **54(2)**: 103-9.
92. Morgenstern LB, Zahuranec DB, Lim J, et al. Tissue-Based Stroke Definition Impacts Stroke Incidence but not Ethnic Differences. *Journal of Stroke and Cerebrovascular Diseases* 2021; **30(6) (no pagination)**.
93. Mortensen JK, Larsson H, Johnsen SP, Andersen G. Impact of prestroke selective serotonin reuptake inhibitor treatment on stroke severity and mortality. *Stroke* 2014; **45(7)**: 2121-3.
94. Mosalski S, Shiner CT, Lannin NA, et al. Increased Relative Functional Gain and Improved Stroke Outcomes: A Linked Registry Study of the Impact of Rehabilitation. *Journal of Stroke and Cerebrovascular Diseases* 2021; **30(10) (no pagination)**.
95. Myint PK, Bachmann MO, Loke YK, et al. Important factors in predicting mortality outcome from stroke: Findings from the Anglia Stroke clinical network evaluation study. *Age and Ageing* 2017; **46(1)**: 83-90.
96. Olsen TS, Andersen KK. Absolute risk of ischemic and hemorrhagic stroke in Danish women using oral contraceptives. *Acta Neurologica Scandinavica* 2022; **145(5)**: 565-70.
97. Oveisgharan S, Ghaemmaghami AB, Bahonar A, Sarrafzadegan N. Case fatality rate and disability of stroke in Isfahan, Iran: Isfahan stroke registry. *Iranian Journal of Neurology* 2016; **15(1)**: 9-15.
98. Palnum KD, Andersen G, Ingeman A, Krog BR, Bartels P, Johnsen SP. Sex-related differences in quality of care and short-term mortality among patients with acute stroke in denmark a nationwide follow-up study. *Stroke* 2009; **40(4)**: 1134-9.
99. Palnum KD, Petersen P, Sorensen HT, et al. Older patients with acute stroke in Denmark: Quality of care and short-term mortality. A nationwide follow-up study. *Age and Ageing* 2008; **37(1)**: 90-5.
100. Pastor S, de Celis E, Losantos García I, Alonso de Leciñana M, Fuentes B, Díez-Tejedor E. Development of the Madrid Stroke Programme: Milestones and Changes in Stroke Trends and Mortality from 1997 to 2017. *Neuroepidemiology* 2021; **55**(2): 135-40.
101. Pedersen TGB, Vinter N, Schmidt M, et al. Trends in the incidence and mortality of intracerebral hemorrhage, and the associated risk factors, in Denmark from 2004 to 2017. *European Journal of Neurology* 2022; **29(1)**: 168-77.

102. Peeters MTJ, Vroman F, Schreuder TAHCML, van Oostenbrugge RJ, Staals J. Decrease in incidence of oral anticoagulant-related intracerebral hemorrhage over the past decade in the Netherlands. *European Stroke Journal* 2022; **7(1)**: 20-7.
103. Peng Y, Ngo L, Hay K, Alghamry A, Colebourne K, Ranasinghe I. Long-Term Survival, Stroke Recurrence, and Life Expectancy After an Acute Stroke in Australia and New Zealand From 2008-2017: A Population-Wide Cohort Study. *Stroke* 2022; **53**(8): 2538-48.
104. Phan HT, Gall SL, Blizzard CL, et al. Sex Differences in Care and Long-Term Mortality after Stroke: Australian Stroke Clinical Registry. *Journal of Women's Health* 2019; **28(5)**: 712-20.
105. Qin H, Chen Y, Liu G, et al. Management characteristics and prognosis after stroke in China: Findings from a large nationwide stroke registry. *Stroke and Vascular Neurology* 2021; **6(1)**: 1-6.
106. Qin H, Wang A, Zuo Y, et al. Malnutrition could Predict 3-month Functional Prognosis in Mild Stroke Patients: Findings from a Nationwide Stroke Registry. *Current Neurovascular Research* 2021; **18(5)**: 489-96.
107. Qureshi AI, Afzal MR, Malik AA, Qureshi MH, Jahangir N, Suri MF. A Population-Based Study of the Incidence and Case Fatality of Intracerebral Hemorrhage of Undetermined Etiology. *J Vasc Interv Neurol* 2015; **8**(4): 17-21.
108. Reid JM, Dai D, Cheripelli B, et al. Differences in wake-up and unknown onset stroke examined in a stroke registry. *International Journal of Stroke* 2015; **10(3)**: 331-5.
109. Roberts SE, Thorne K, Akbari A, Samuel DG, Williams JG. Mortality following stroke, the weekend effect and related factors: Record linkage study. *PLoS ONE* 2015; **10(6) (no pagination)**.
110. Rumana N, Kita Y, Turin TC, et al. Acute case-fatality rates of stroke and acute myocardial infarction in a Japanese population: Takashima stroke and AMI registry, 1989-2005. *International Journal of Stroke* 2014; **9(A100)**: 69-75.
111. Rusek L, Persson CU, Svardsudd K, et al. Lifetime risk of stroke in the general male population. *Acta neurologica Scandinavica* 2020; **23**.
112. Rutten-Jacobs LCA, Maaijwee NAM, Arntz RM, et al. Clinical characteristics and outcome of intracerebral hemorrhage in young adults. *Journal of Neurology* 2014; **261(11)**: 2143-9.
113. Sarfo FS, Akpa OM, Ovbiagele B, et al. Patient-level and system-level determinants of stroke fatality across 16 large hospitals in Ghana and Nigeria: a prospective cohort study. *The Lancet Global Health* 2023; **11(4)**: e575-e85.
114. Schmidt M, Hovath-Puho E, Christiansen CF, Petersen KL, Botker HE, Sorensen HT. Preadmission use of nonaspirin nonsteroidal anti-inflammatory drugs and 30-day stroke mortality. *Neurology* 2014; **83(22)**: 2013-22.
115. Schmidt M, Jacobsen JB, Johnsen SP, Bootker HE, Soorensen HT. Eighteen-year trends in stroke mortality and the prognostic influence of comorbidity. *Neurology* 2014; **82(4)**: 340-50.
116. Sedova P, Brown RD, Zvolsky M, et al. Incidence of Stroke and Ischemic Stroke Subtypes: A Community-Based Study in Brno, Czech Republic. *Cerebrovascular diseases (Basel, Switzerland)* 2020: 1-8.
117. Sedova P, Brown RD, Zvolsky M, et al. Incidence of Hospitalized Stroke in the Czech Republic: The National Registry of Hospitalized Patients. *Journal of Stroke and Cerebrovascular Diseases* 2017; **26(5)**: 979-86.
118. Sennfält S, Norrving B, Petersson J, Ullberg T. Long-Term Survival and Function After Stroke. *Stroke* 2018: Strokeaha118022913.
119. Sennfalt S, Norrving B, Petersson J, Ullberg T. Long-Term Survival and Function after Stroke: A Longitudinal Observational Study from the Swedish Stroke Register. *Stroke* 2019; **50(1)**: 53-61.
120. Shigematsu K, Watanabe Y, Nakano H. Lower hazard ratio for death in women with cerebral hemorrhage. *Acta Neurologica Scandinavica* 2015; **132(1)**: 59-64.
121. Sipila JOT, Posti JP, Ruuskanen JO, Rautava P, Kyto V. Stroke hospitalization trends of the working-aged in Finland. *PLoS ONE* 2018; **13(8) (no pagination)**.
122. Sipila JOT, Ruuskanen JO, Rautava P, Kyto V. Case fatality of hospital-treated intracerebral hemorrhage in Finland - A nationwide population-based registry study. *Journal of the Neurological Sciences* 2021; **425 (no pagination)**.
123. Skajaa N, Adelborg K, Horvath-Puho E, et al. Nationwide Trends in Incidence and Mortality of Stroke Among Younger and Older Adults in Denmark. *Neurology* 2021; **96(13)**: E1711-E23.
124. Starostka-Tatar A, Labuz-Roszak B, Skrzypek M, Lasek-Bal A, Gasior M, Gierlotka M. Characteristics of hospitalizations due to acute stroke in the Silesian Province, Poland, between 2009 and 2015. *Neurologia i Neurochirurgia Polska* 2018; **52(2)**: 252-62.
125. Stein M, Hamann GF, Misselwitz B, Uhl E, Kolodziej M, Reinges MHT. In-Hospital Mortality and Complication Rates in Surgically and Conservatively Treated Patients with Spontaneous Intracerebral Hemorrhage in Central Europe: A Population-Based Study. *World Neurosurgery* 2016; **88**: 306-10.
126. Sung SF, Su CC, Hsieh CY, et al. Home-time as a surrogate measure for functional outcome after stroke: A validation study. *Clinical Epidemiology* 2020; **12**: 617-24.

127. Suzuki K, Izumi M. The incidence of hemorrhagic stroke in Japan is twice compared with western countries: the Akita stroke registry. *Neurological Sciences* 2015; **36(1)**: 155-60.
128. Suzuki K, Izumi M, Sakamoto T, Hayashi M. Blood pressure and total cholesterol level are critical risks especially for hemorrhagic stroke in Akita, Japan. *Cerebrovascular Diseases* 2011; **31(1)**: 100-6.
129. Takashima N, Arima H, Turin TC, et al. The 21-Year Trend of Stroke Incidence in a General Japanese Population: Results from the Takashima Stroke Registry, 1990-2010. *Cerebrovascular Diseases* 2022.
130. Teles J, Martinez J, Mouzinho M, Guilherme P, Marreiros A, Nzwalo H. Gender differences in long-term mortality after spontaneous intracerebral hemorrhage in southern Portugal. *Porto Biomed J* 2021; **6**(4): e137.
131. Tessua KK, Munseri P, Matuja SS. Outcomes within a year following first ever stroke in Tanzania. *PLoS ONE* 2021; **16(2 February) (no pagination)**.
132. Turin TC, Kita Y, Murakami Y, et al. Higher stroke incidence in the spring season regardless of conventional risk factors: Takashima Stroke Registry, Japan, 1988-2001. *Stroke* 2008; **39(3)**: 745-52.
133. Ullberg T, Zia E, Petersson J, Norrving B. Changes in functional outcome over the first year after stroke: An observational study from the Swedish stroke register. *Stroke* 2015; **46(2)**: 389-94.
134. Wang IK, Yen TH, Tsai CH, et al. Renal function is associated with one-month and one-year mortality in patients with intracerebral hemorrhage. *PLoS ONE* 2023; **18(1 January) (no pagination)**.
135. Wang WJ, Lu JJ, Wang YJ, et al. Clinical Characteristics, Management, and Functional Outcomes in Chinese Patients Within the First Year After Intracerebral Hemorrhage: Analysis from China National Stroke Registry. *CNS Neuroscience and Therapeutics* 2012; **18(9)**: 773-80.
136. Wang W, Lu J, Wang C, Wang Y, Li H, Zhao X. Prognostic Value of ICH Score and ICH-GS Score in Chinese Intracerebral Hemorrhage Patients: Analysis From the China National Stroke Registry (CNSR). *PLoS ONE* 2013; **8(10) (no pagination)**.
137. Wang Y, Cui L, Ji X, et al. The China National Stroke Registry for patients with acute cerebrovascular events: design, rationale, and baseline patient characteristics. *Int J Stroke* 2011; **6**(4): 355-61.
138. Wang Z, Li J, Wang C, et al. Gender Differences in 1-Year Clinical Characteristics and Outcomes after Stroke: Results from the China National Stroke Registry. *PLoS ONE* 2013; **8(2) (no pagination)**.
139. Wen CP, Lee YC, Sun YT, et al. Low-Density Lipoprotein Cholesterol and Mortality in Patients With Intracerebral Hemorrhage in Taiwan. *Frontiers in Neurology* 2021; **12 (no pagination)**.
140. Wen CP, Liu CH, Jeng JS, et al. Pre-stroke physical activity is associated with fewer post-stroke complications, lower mortality and a better long-term outcome. *European Journal of Neurology* 2017; **24(12)**: 1525-31.
141. Yeo SH, Yau WP. Temporal Trends and Predictors of Drug Utilization and Outcomes in First-Ever Stroke Patients: A Population-Based Study Using the Singapore Stroke Registry. *CNS Drugs* 2019; **33(8)**: 791-815.
142. Youkee D, Deen G, Barrett E, et al. A Prospective Stroke Register in Sierra Leone: Demographics, Stroke Type, Stroke Care and Hospital Outcomes. *Frontiers in Neurology* 2021; **12 (no pagination)**.
143. Youkee D, Deen GF, Baldeh M, et al. Stroke in Sierra Leone: Case fatality rate and functional outcome after stroke in Freetown. *International Journal of Stroke* 2023.
144. Zeng YJ, Liu GF, Liu LP, Wang CX, Zhao XQ, Wang YJ. Anemia on admission increases the risk of mortality at 6 months and 1 year in hemorrhagic stroke patients in China. *Journal of Stroke and Cerebrovascular Diseases* 2014; **23(6)**: 1500-5.
145. Zhang Y, Ji R, Wang A, et al. In-hospital complications affect short-term and long-term mortality in ICH: A prospective cohort study. *Stroke and Vascular Neurology* 2021; **6(2)**: 201-6.
146. Zhukov Y, Dyussembekov YKK, Aringazina A, et al. Time Trends of Epidemiology of Hemorrhagic Stroke among Urban Population in Kazakhstan. *Open Access Macedonian Journal of Medical Sciences* 2022; **Part A. 10**: 402-8.

ii. Not prospective (n = 38)

1. Ban L, Sprigg N, Sultan AA, et al. Incidence of First Stroke in Pregnant and Nonpregnant Women of Childbearing Age: A Population-Based Cohort Study From England. *Journal of the American Heart Association* 2017; **6(4) (no pagination)**.
2. Ferket BS, Van Kempen BJH, Wieberdink RG, et al. Separate prediction of intracerebral hemorrhage and ischemic stroke. *Neurology* 2014; **82(20)**: 1804-12.
3. Gattellari M, Goumas C, Jalaludin B, Worthington JM. Population-based stroke surveillance using big data: state-wide epidemiological trends in admissions and mortality in New South Wales, Australia. *Neurological research* 2020: 1-10.
4. Gulliford MC, Charlton J, Rudd A, Wolfe CD, Toschke AM. Declining 1-year case-fatality of stroke and increasing coverage of vascular risk management: population-based cohort study. *J Neurol Neurosurg Psychiatry* 2010; **81**(4): 416-22.
5. He W, Liu Y, Feng J, et al. The epidemiological characteristics of stroke in Hunan Province, China. *Frontiers in Neurology* 2018; **9(JUL) (no pagination)**.

6. Heeley EL, Wei JW, Carter K, et al. Socioeconomic disparities in stroke rates and outcome: Pooled analysis of stroke incidence studies in Australia and New Zealand. *Medical Journal of Australia* 2011; **195(1)**: 10-4.

7. Kang JH, Wu CS, Keller JJ, Lin HC. Chronic rhinosinusitis increased the risk of stroke: A 5-year follow-up study. *Laryngoscope* 2013; **123(4)**: 835-40.

8. Kapral MK, Fang J, Silver FL, et al. Effect of a provincial system of stroke care delivery on stroke care and outcomes. *Cmaj* 2013; **Canadian Medical Association Journal. 185(10)**: E483-E91.

9. Khedr EM, Fawi G, Abdela M, et al. Prevalence of ischemic and hemorrhagic strokes in Qena Governorate, Egypt: community-based study. *J Stroke Cerebrovasc Dis* 2014; **23**(7): 1843-8.

10. Kim JY, Kang K, Kang J, et al. Executive summary of stroke statistics in Korea 2018: A report from the epidemiology research council of the korean stroke society. *Journal of Stroke* 2019; **21(1)**: 42-59.

11. Lekander I, Willers C, Ekstrand E, et al. Hospital comparison of stroke care in Sweden: A register-based study. *BMJ Open* 2017; **7(9) (no pagination)**.

12. Li FE, Luo Y, Zhang FL, et al. Association Between Cardiometabolic Index and Stroke: A Population- based Cross-sectional Study. *Curr Neurovasc Res* 2021; **18**(3): 324-32.

13. Ma T, Yazdi MD, Schwartz J, et al. Long-term air pollution exposure and incident stroke in American older adults: A national cohort study. *Glob Epidemiol* 2022; **4**.

14. McAlister FA, Yan L, Roos LL, Lix LM. Parental Atrial Fibrillation and Stroke or Atrial Fibrillation in Young Adults: A Population-Based Cohort Study. *Stroke* 2019; **50(9)**: 2322-8.

15. Moyano LM, Montano SM, Barreto PV, et al. Prevalence of stroke survival in rural communities living in northern Peru. *PLoS ONE* 2021; **16(7 July) (no pagination)**.

16. Polcaro-Pichet S, Kosatsky T, Potter BJ, Bilodeau-Bertrand M, Auger N. Effects of cold temperature and snowfall on stroke mortality: A case-crossover analysis. *Environment International* 2019; **126**: 89-95.

17. Saliba W, Barnett-Griness O, Gronich N, et al. Association of diabetes and glycated hemoglobin with the risk of intracerebral hemorrhage: A population-based cohort study. *Diabetes Care* 2019; **42(4)**: 682-8.

18. Schieb LJ, Ayala C, Valderrama AL, Veazie MA. Trends and disparities in stroke mortality by region for American Indians and Alaska Natives. *American journal of public health* 2014; **104 Suppl 3**: S368-76.

19. Schols AMR, Schreuder FHBM, Van Raak EPM, et al. Incidence of oral anticoagulant-associated intracerebral hemorrhage in the Netherlands. *Stroke* 2014; **45(1)**: 268-70.

20. Shah R, Wilkins E, Nichols M, et al. Epidemiology report: Trends in sex-specific cerebrovascular disease mortality in Europe based on WHO mortality data. *European Heart Journal* 2019; **40(9)**: 755-64.

21. Shaheen HA, Abdel Wahed WY, Hasaneen ST. Prevalence of Stroke in Fayoum Governorate, Egypt: A Community-Based Study. *Journal of Stroke and Cerebrovascular Diseases* 2019; **28(9)**: 2414-22. Shin S, Burnett RT, Kwong JC, et al. Ambient Air Pollution and the Risk of Atrial Fibrillation and Stroke: A Population-Based Cohort Study. *Environ Health Perspect* 2019; **127**(8): 87009.

23. Sun HX, Wang WZ. A nationwide epidemiological sample survey on cerebrovascular disease in China. [Chinese]. *Chinese Journal of Contemporary Neurology and Neurosurgery* 2018; **18(2)**: 83-8.

24. Tian DS, Liu CC, Wang CL, et al. Prevalence and risk factors of stroke in China: a national serial cross-sectional study from 2003 to 2018. *Stroke Vasc Neurol* 2022.

25. Vaartjes I, Reitsma JB, De Bruin A, et al. Nationwide incidence of first stroke and TIA in the Netherlands. *European Journal of Neurology* 2008; **15(12)**: 1315-23.

26. Wang H, Lee PMY, Zhang J, Svendsen K, Li F, Li J. Association of intellectual disability with overall and type-specific cardiovascular diseases: a population-based cohort study in Denmark. *BMC Medicine* 2023; **21(1) (no pagination)**.

27. Wang W, Jiang B, Sun H, et al. Prevalence, Incidence, and Mortality of Stroke in China: Results from a Nationwide Population-Based Survey of 480 687 Adults. *Circulation* 2017; **135(8)**: 759-71.

28. Wang W, Jiang B, Sun H, et al. Prevalence, Incidence, and Mortality of Stroke in China: Results from a Nationwide Population-Based Survey of 480 687 Adults. *Circulation* 2017; **135**(8): 759-71.

29. Wang W, Rudd AG, Wang Y, et al. Risk prediction of 30-day mortality after stroke using machine learning: a nationwide registry-based cohort study. *BMC Neurology* 2022; **22(1) (no pagination)**.

30. Wang X, Jiang G, Choi BC, et al. Surveillance of trend and distribution of stroke mortality by subtype, age, gender, and geographic areas in Tianjin, China, 1999-2006. *Int J Stroke* 2009; **4**(3): 169-74.

31. Wang YJ, Li ZX, Gu HQ, et al. China Stroke Statistics 2019: A Report From the National Center for Healthcare Quality Management in Neurological Diseases, China National Clinical Research Center for Neurological Diseases, the Chinese Stroke Association, National Center for Chronic and. *Stroke and vascular neurology* 2020; **21**.

32. Wang Z, Liu W, Ren Y, et al. Loss of life expectancy due to stroke and its subtypes in urban and rural areas in China, 2005-2020. *Stroke and vascular neurology* 2023; **06**.

33. Woodward M, Tsukinoki-Murakami R, Murakami Y, et al. The epidemiology of stroke amongst women in the Asia - Pacific region. *Women's Health* 2011; **7(3)**: 305-17.

34. Wu YY, Chen PY, Wu CC, et al. Long-term mortality rates of young stroke in Taiwan: A decade-long epidemiology population-based study. *European Stroke Journal* 2022; **7(4)**: 447-55.
35. Yang Q, Tong X, Schieb L, et al. Vital Signs: Recent Trends in Stroke Death Rates - United States, 2000-2015. *Mmwr* 2017; **Morbidity and mortality weekly report. 66(35)**: 933-9.
36. Zhai Y, Wang WZ, Zhao WH, Yang XG, Kong LZ. [The prevalence and onset of age of stroke in Chinese adults]. *Zhonghua Yu Fang Yi Xue Za Zhi* 2009; **43**(12): 1069-72.
37. Zhou L, Chen K, Chen X, et al. Heat and mortality for ischemic and hemorrhagic stroke in 12 cities of Jiangsu Province, China. *Science of the Total Environment* 2017; **601-602**: 271-7.
38. Zhou S, Sun X, Zhou X, Xu G, Wang S. Epidemiology of stroke and its risk factors in Shandong province, China. [Chinese]. *Chinese Journal of Neurology* 2019; **52(9)**: 716-23.

iii. Not first-ever ICH (n = 8)

1. Callaly E, Ni Chroinin D, Hannon N, et al. Rates, predictors, and outcomes of early and late recurrence after stroke: The North Dublin population stroke study. *Stroke* 2016; **47(1)**: 244-6.
2. Cottel D, Montaye M, Marecaux N, Amouyel P, Dallongeville J, Meirhaeghe A. Comparison of the rates of stroke and acute coronary events in northern France. *European Journal of Preventive Cardiology* 2018; **25(14)**: 1534-42.
3. Gueniat J, Breniere C, Graber M, et al. Increasing Burden of Stroke: The Dijon Stroke Registry (1987-2012). *Neuroepidemiology* 2018; **50(1-2)**: 47-56.
4. Ishikawa S, Kayaba K, Gotoh T, et al. Incidence of total stroke, stroke subtypes, and myocardial infarction in the Japanese population: the JMS Cohort Study. *Journal of epidemiology / Japan Epidemiological Association* 2008; **18(4)**: 144-50.
5. Lainay C, Benzenine E, Durier J, et al. Hospitalization within the first year after stroke: The dijon stroke registry. *Stroke* 2015; **46(1)**: 190-6.
6. Li L, Luengo-Fernandez R, Zuurbier SM, et al. Ten-year risks of recurrent stroke, disability, dementia and cost in relation to site of primary intracerebral haemorrhage: population-based study. *J Neurol Neurosurg Psychiatry* 2020; **91**(6): 580-5.
7. Lisabeth LD, Smith MA, Sanchez BN, Brown DL. Ethnic disparities in stroke and hypertension among women: The BASIC Project. *American Journal of Hypertension* 2008; **21(7)**: 778-83.
8. Shoeibi A, Salehi M, Thrift AG, et al. One-year case fatality rate following stroke in the Mashhad Stroke Incidence Study: A population-based study of stroke in Iran. *International Journal of Stroke* 2015; **10(A100)**: 96-102.

iv. Design does not allow calculation of outcome of interest (n = 52)

1. Abreu P, Magalhaes R, Baptista D, Azevedo E, Silva MC, Correia M. Readmissions and Mortality During the First Year After Stroke-Data From a Population-Based Incidence Study. *Frontiers in Neurology* 2020; **11 (no pagination)**.
2. Aghaali M, Yoosefee S, Hejazi SA, et al. A prospective population-based study of stroke in the Central Region of Iran: The Qom Incidence of Stroke Study. *International Journal of Stroke* 2022; **17(9)**: 957-63.
3. Aked J, Delavaran H, Lindgren AG. Survival, causes of death and recurrence up to 3 years after stroke: A population-based study. *European Journal of Neurology* 2021; **28(12)**: 4060-8.
4. Andalibi MSS, Rezaei Ardani A, Amiri A, et al. The Association between Substance Use Disorders and Long-Term Outcome of Stroke: Results from a Population-Based Study of Stroke among 450,229 Urban Citizens. *Neuroepidemiology* 2021; **55(3)**: 171-9.
5. Appelros P, Arvidsson-Lindvall M, Materne M. Stroke prevalence in a medium-sized Swedish municipality. *Acta Neurologica Scandinavica* 2021; **143(2)**: 210-6.
6. Bejot Y, Aboa-Eboule C, Hervieu M, et al. The deleterious effect of admission hyperglycemia on survival and functional outcome in patients with intracerebral hemorrhage. []. *Stroke A Journal of Cerebral Circulation* 2011; **22**.
7. Bejot Y, Blanc C, Delpont B, et al. Increasing early ambulation disability in spontaneous intracerebral hemorrhage survivors. *Neurology* 2018; **90(23)**: e2017-e24.
8. Bhalla A, Wang Y, Rudd A, Wolfe CDA. Differences in outcome and predictors between ischemic and intracerebral hemorrhage: The South London Stroke Register. *Stroke* 2013; **44(8)**: 2174-81.
9. Busch MA, Coshall C, Heuschmann PU, McKevitt C, Wolfe CDA. Sociodemographic differences in return to work after stroke: The South London Stroke Register (SLSR). *Journal of Neurology, Neurosurgery and Psychiatry* 2009; **80(8)**: 888-93.
10. Carlos CB, Majersik JJ, Sanchez BN, et al. Brain attack surveillance in a Mexican community: Design of a population-base study of cerebrovasculares diseases in Mexico. [Spanish]. *Revista Mexicana de Neurociencia* 2010; **11(2)**: 128-35.

11. Chen R, McKevitt C, Rudd AG, Wolfe CDA. Socioeconomic deprivation and survival after stroke findings from the prospective south london stroke register of 1995 to 2011. *Stroke* 2014; **45(1)**: 217-23.
12. Chen Z, Jiang B, Ru X, et al. Mortality of Stroke and Its Subtypes in China: Results from a Nationwide Population-Based Survey. *Neuroepidemiology* 2017; **48(3-4)**: 95-102.
13. Crichton S, Barratt B, Spiridou A, et al. Associations between exhaust and non-exhaust particulate matter and stroke incidence by stroke subtype in South London. *Science of the Total Environment* 2016; **568**: 278-84.
14. Del Brutto O, Mera R, Del Brutto V. Nonfatal stroke and all-cause mortality among community-dwelling older adults living in rural Ecuador: A population-based, prospective study. *Journal of Neurosciences in Rural Practice* 2018; **9(4)**: 551-5.
15. Diaz-Guzman J, Egido-Herrero JA, Fuentes B, et al. Incidence of strokes in Spain: The iberictus study. Data from the pilot study. [Spanish]. *Revista de Neurologia* 2009; **48(2)**: 61-5.
16. Elfassy T, Grasset L, Glymour MM, et al. Sociodemographic Disparities in Long-Term Mortality Among Stroke Survivors in the United States: The REGARDS Study. *Stroke* 2019; **50(4)**: 805-12.
17. Feigin VL, Barker-Collo S, Parag V, et al. Auckland Stroke Outcomes Study: Part 1: Gender, stroke types, ethnicity, and functional outcomes 5 years poststroke. *Neurology* 2010; **75(18)**: 1597-607.
18. Gardiner FW, Rallah-Baker K, Dos Santos A, et al. Indigenous Australians have a greater prevalence of heart, stroke, and vascular disease, are younger at death, with higher hospitalisation and more aeromedical retrievals from remote regions. *eClinicalMedicine* 2021; **42 (no pagination)**.
19. Griauzde J, Lisabeth LD, Li C, et al. A Population-Based Study of Intracerebral Hemorrhage Survivors' Outcomes. *Journal of Stroke and Cerebrovascular Diseases* 2019; **28(1)**: 49-55.
20. Guo W, Du M, Sun D, et al. The effect characteristics of temperature on stroke mortality in Inner Mongolia and globally. *International journal of biometeorology* 2019; **63(2)**: 159-66.
21. Hannon N, Sheehan O, Kelly L, et al. Stroke associated with atrial fibrillation - Incidence and early outcomes in the north dublin population stroke study. *Cerebrovascular Diseases* 2010; **29(1)**: 43-9.
22. Hansen BM, Nilsson OG, Anderson H, Norrving B, Saveland H, Lindgren A. Long term (13 years) prognosis after primary intracerebral haemorrhage: A prospective population based study of long term mortality, prognostic factors and causes of death. *Journal of Neurology, Neurosurgery and Psychiatry* 2013; **84(10)**: 1150-5.
23. Howard VJ, McClure LA, Glymour MM, et al. Effect of duration and age at exposure to the Stroke Belt on incident stroke in adulthood. *Neurology* 2013; **80**(18): 1655-61.
24. Huhtakangas J, Tetri S, Juvela S, Saloheimo P, Bode MK, Hillbom M. Effect of increased warfarin use on warfarin-related cerebral hemorrhage: A longitudinal population-based study. *Stroke* 2011; **42(9)**: 2431-5.
25. Inoue N, Okamura T, Kokubo Y, et al. LOX index, a novel predictive biochemical marker for coronary heart disease and stroke. *Clinical Chemistry* 2010; **56(4)**: 550-8.
26. Jonsson AC, Delavaran H, Iwarsson S, Stahl A, Norrving B, Lindgren A. Functional status and patient-reported outcome 10 years after stroke: The lund stroke register. *Stroke* 2014; **45(6)**: 1784-90.
27. Khellaf M, Quantin C, D'Athis P, et al. Age-period-cohort analysis of stroke incidence in dijon from 1985 to 2005. *Stroke* 2010; **41(12)**: 2762-7.
28. Kissela BM, Khoury JC, Alwell K, et al. Age at stroke: Temporal trends in stroke incidence in a large, biracial population. *Neurology* 2012; **79(17)**: 1781-7.
29. Li J, Imano H, Kitamura A, et al. Trends in the incidence of stroke and its subtypes from 1963 to 2018 in Japanese urban and rural communities: The Circulatory Risk in Communities Study (CIRCS). *International Journal of Stroke* 2022.
30. Li J, Imano H, Yamagishi K, et al. Leukocyte Count and Risks of Stroke and Coronary Heart Disease: The Circulatory Risk in Communities Study (CIRCS). *J Atheroscler Thromb* 2022; **29**(4): 527-35.
31. Madsen TE, Cummings OW, De Los Rios La Rosa F, et al. Substance Use and Performance of Toxicology Screens in the Greater Cincinnati Northern Kentucky Stroke Study. *Stroke* 2022; **53**(10): 3082-90.
32. Maheswaran R, Pearson T, Smeeton NC, Beevers SD, Campbell MJ, Wolfe CD. Impact of outdoor air pollution on survival after stroke: Population-based cohort study. *Stroke* 2010; **41(5)**: 869-77.
33. McCormick J, Chen R. Impact of socioeconomic deprivation on mortality in people with haemorrhagic stroke: A population-based cohort study. *Postgraduate Medical Journal* 2016; **92(1091)**: 501-5.
34. McNaughton H, Feigin V, Kerse N, et al. Ethnicity and functional outcome after stroke. *Stroke* 2011; **42(4)**: 960-4.
35. Movahed MS, Barghazan SH, Adel A, Rezapour A. Economic Burden of Stroke in Iran: A Population-Based Study. *Value in Health Regional Issues* 2021; **24**: 77-81.
36. Obaid M, Flach C, Marshall I, C DAW, Douiri A. Long-Term Outcomes in Stroke Patients with Cognitive Impairment: A Population-Based Study. *Geriatrics (Basel)* 2020; **5**(2).
37. Onwuchekwa AC, Tobin-West C, Babatunde S. Prevalence and risk factors for stroke in an adult population in a rural community in the Niger Delta, south-south Nigeria. *J Stroke Cerebrovasc Dis* 2014; **23**(3): 505-10.

38. Pendlebury ST, Rothwell PM. Incidence and prevalence of dementia associated with transient ischaemic attack and stroke: analysis of the population-based Oxford Vascular Study. *The Lancet Neurology* 2019; **18(3)**: 248-58.
39. Radisauskas R, Tamosiunas A, Kranciukaite-Butylkiniene D, et al. Long-term survival after stroke in Lithuania: Data from Kaunas population-based stroke registry. *PLoS ONE* 2019; **14(7) (no pagination)**.
40. Rannikmae K, Ngoh K, Bush K, et al. Accuracy of identifying incident stroke cases from linked health care data in UK Biobank. *Neurology* 2020; **95(6)**: E697-E707.
41. Romain G, Mariet AS, Jooste V, et al. Long-Term Relative Survival after Stroke: The Dijon Stroke Registry. *Neuroepidemiology* 2020; **54(6)**: 498-505.
42. Ru X, Wang W, Sun H, et al. GeographicalDifference, Rural-urban Transition and Trend in Stroke Prevalence in China: Findings from a National Epidemiological Survey of Stroke in China. *Sci Rep* 2019; **9**(1): 17330.
43. Sarker SJ, Heuschmann PU, Burger I, et al. Predictors of survival after haemorrhagic stroke in a multi-ethnic population: the South London Stroke Register (SLSR). *J Neurol Neurosurg Psychiatry* 2008; **79**(3): 260-5.
44. Schnitzler A, Woimant F, Tuppin P, De Peretti C. Prevalence of self-reported stroke and disability in the French adult population: A transversal study. *PLoS ONE* 2014; **9(12) (no pagination)**.
45. Sen A, Bisquera A, Wang Y, et al. Factors, trends, and long-term outcomes for stroke patients returning to work: The South London Stroke Register. *International Journal of Stroke* 2019.
46. Sergeev AV. Stroke mortality disparities in the population of the appalachian mountain region. *Ethnicity and Disease* 2013; **23(3)**: 286-91.
47. Shigematsu K, Watanabe Y, Nakano H. Higher ratio of ischemic stroke to hemorrhagic stroke in summer. *Acta Neurologica Scandinavica* 2015; **132(6)**: 423-9.
48. Vibo R, Schneider S, Korv J. Long-term survival of young stroke patients: A population-based study of two stroke registries from Tartu, Estonia. *Stroke Research and Treatment* 2012; **(no pagination)**.
49. Wang Y, Rudd AG, Wolfe CDA. Trends and Survival between Ethnic Groups after Stroke: The South London Stroke Register. *Stroke* 2013; **44(2)**: 380-7.
50. Waziry R, Heshmatollah A, Bos D, et al. Time trends in survival following first hemorrhagic or ischemic stroke between 1991 and 2015 in the Rotterdam Study. *Stroke* 2020: 824-9.
51. Xu L, Schooling CM, Chan WM, Lee SY, Leung GM, Lam TH. Smoking and hemorrhagic stroke mortality in a prospective cohort study of older Chinese. *Stroke* 2013; **44(8)**: 2144-9.
52. Zhang Y, Galloway JM, Welty TK, et al. Incidence and risk factors for stroke in american indians the strong heart study. *Circulation* 2008; **118(15)**: 1577-84.

v. ICH not described separately (n = 132)

1. Abdullah Said M, Verweij N, Van Der Harst P. Associations of combined genetic and lifestyle risks with incident cardiovascular disease and diabetes in the UK biobank study. *JAMA Cardiology* 2018; **3(8)**: 693-702.
2. Ayis S, Wellwood I, Rudd AG, McKevitt C, Parkin D, Wolfe CDA. Variations in Health-Related Quality of Life (HRQoL) and survival 1 year after stroke: Five European population-based registers. *BMJ Open* 2015; **5(6) (no pagination)**.
3. Azarpazhooh MR, Shahripour RB, Kapral MK, et al. Incidence of first ever stroke during Hajj ceremony. *BMC Neurology* 2013; **13 (no pagination)**.
4. Baena-Díez JM, Vidal-Solsona M, Byram AO, González-Casafont I, Ledesma-Ulloa G, Martí-Sans N. The epidemiology of cardiovascular disease in primary care. the Zona Franca Cohort study in Barcelona, Spain. *Rev Esp Cardiol* 2010; **63**(11): 1261-9.
5. Balabanski AH, Newbury J, Leyden JM, et al. Excess stroke incidence in young Aboriginal people in South Australia: Pooled results from two population-based studies. *International Journal of Stroke* 2018; **13(8)**: 811-4.
6. Béjot Y, Bailly H, Graber M, et al. Impact of the Ageing Population on the Burden of Stroke: The Dijon Stroke Registry. *Neuroepidemiology* 2019; **52**(1-2): 78-85.
7. Bejot Y, Duloquin G, Graber M, Garnier L, Mohr S, Giroud M. Current characteristics and early functional outcome of older stroke patients: a population-based study (Dijon Stroke Registry). *Age and Ageing* 2021; **50(3)**: 898-905.
8. Bhalla A, Smeeton N, Rudd AG, Heuschmann P, Wolfe CDA. A comparison of characteristics and resource use between in-hospital and admitted patients with stroke. *Journal of Stroke and Cerebrovascular Diseases* 2010; **19(5)**: 357-63.
9. Budoff MJ, Young R, Burke G, et al. Ten-year association of coronary artery calcium with atherosclerotic cardiovascular disease (ASCVD) events: the multi-ethnic study of atherosclerosis (MESA). *European Heart Journal* 2018; **39(25)**: 2401b-8b.
10. Busch MA, Schienkiewitz A, Nowossadeck E, Gößwald A. [Prevalence of stroke in adults aged 40 to 79 years in Germany: results of the German Health Interview and Examination Survey for Adults (DEGS1)]. *Bundesgesundheitsblatt Gesundheitsforschung Gesundheitsschutz* 2013; **56**(5-6): 656-60.

11. Cerasuolo JO, Cipriano LE, Sposato LA, et al. Population-based stroke and dementia incidence trends: Age and sex variations. *Alzheimer's and Dementia* 2017; **13(10)**: 1081-8.
12. Chan YM, Ganapathy SS, Tan L, Alias N, Nasaruddin NH, Khaw WF. The burden of premature mortality among older adults: a population-based study in Malaysia. *BMC public health* 2022; **22(1)**: 1181.
13. Chausson N, Olindo S, Cabre P, Saint-Vil M, Smadja D. Five-year outcome of a stroke cohort in martinique, French West Indies: Etude realisee en martinique et centree sur l'incidence des accidents vasculaires cerebraux, part 2. *Stroke* 2010; **41(4)**: 594-9.
14. Cheung CY, Tay WT, Ikram MK, et al. Retinal microvascular changes and risk of stroke: the Singapore Malay Eye Study. *Stroke* 2013; **44**(9): 2402-8.
15. Clua-Espuny JL, Piñol-Moreso JL, Panisello-Tafalla A, et al. [Ebrictus study. Functional results, survival, and potential years of life lost after the first stroke]. *Aten Primaria* 2012; **44**(4): 223-31.
16. Crichton SL, Bray BD, McKevitt C, Rudd AG, Wolfe CD. Patient outcomes up to 15 years after stroke: survival, disability, quality of life, cognition and mental health. *J Neurol Neurosurg Psychiatry* 2016; **87**(10): 1091-8.
17. Cushman M, Cantrell RA, McClure LA, et al. Estimated 10-year stroke risk by region and race in the United States: Geographic and racial differences in stroke risk. *Annals of Neurology* 2008; **64(5)**: 507-13.
18. Dalmeijer GW, Struijk EA, Van Der Schouw YT, et al. Dairy intake and coronary heart disease or stroke - A population-based cohort study. *International Journal of Cardiology* 2013; **167(3)**: 925-9.
19. Danesi MA, Okubadejo NU, Ojini FI, Ojo OO. Incidence and 30-day case fatality rate of first-ever stroke in urban Nigeria: The prospective community based Epidemiology of Stroke in Lagos (EPISIL) phase II results. *Journal of the Neurological Sciences* 2013; **331(1-2)**: 43-7.
20. De Souza CDF, De Oliveira DJ, Da Silva LF, et al. Cerebrovascular disease mortality trend in Brazil (1996 to 2015) and association with human development index and social vulnerability. *Arquivos Brasileiros de Cardiologia* 2021; **116(1)**: 89-99.
21. Dehghan M, Mente A, Rangarajan S, et al. Association of dairy intake with cardiovascular disease and mortality in 21 countries from five continents (PURE): a prospective cohort study. *Lancet* 2018; **392**(10161): 2288-97.
22. Emmett ES, Douiri A, Marshall IJ, Wolfe CDA, Rudd AG, Bhalla A. A comparison of trends in stroke care and outcomes between in-hospital and community-onset stroke - The South London Stroke Register. *PLoS ONE* 2019; **14(2) (no pagination)**.
23. Fan M, Sun D, Zhou T, et al. Sleep patterns, genetic susceptibility, and incident cardiovascular disease: A prospective study of 385 292 UK biobank participants. *European Heart Journal* 2020; **41(11)**: 1182-9.
24. Feigin VL, Krishnamurthi RV, Barker-Collo S, et al. 30-Year trends in stroke rates and outcome in Auckland, New Zealand (1981-2012): A multi-ethnic population-based series of studies. *PLoS ONE* 2015; **10(8) (no pagination)**.
25. Gao Y, Jiang B, Sun H, et al. The burden of stroke in China: Results from a nationwide population-based epidemiological survey. *PLoS ONE* 2018; **13(12) (no pagination)**.=
26. Glasser SP, Halberg DL, Sands CD, Mosher A, Muntner PM, Howard G. Is Pulse Pressure an Independent Risk Factor for Incident Stroke, REasons for Geographic And Racial Differences in Stroke. *Am J Hypertens* 2015; **28**(8): 987-94.
27. Grimaud O, Leray E, Lalloue B, et al. Mortality following stroke during and after acute care according to neighbourhood deprivation: A disease registry study. *Journal of Neurology, Neurosurgery and Psychiatry* 2014; **85(12)**: 1313-8.
28. Hallstrom B, Jonsson AC, Nerbrand C, Norrving B, Lindgren A. Stroke incidence and survival in the beginning of the 21st century in Southern Sweden: Comparisons with the late 20th century and projections into the future. *Stroke* 2008; **39(1)**: 10-5.
29. Hanchaiphiboolkul S, Puthkhao P, Tantirittisak T, et al. Within-Visit Blood Pressure Variability and All-Cause and Stroke Mortality: Thai Epidemiologic Stroke Study. *Neuroepidemiology* 2022; **56(5)**: 345-54.
30. Heuschmann PU, Wiedmann S, Wellwood I, et al. Three-month stroke outcome: The European Registers of Stroke (EROS) Investigators. *Neurology* 2011; **76(2)**: 159-65.
31. Hoffmann B, Weinmayr G, Hennig F, et al. Air quality, stroke, and coronary events: results of the Heinz Nixdorf Recall Study from the Ruhr Region. *Dtsch Arztebl Int* 2015; **112**(12): 195-201.
32. Howard G, Kleindorfer DO, Cushman M, et al. Contributors to the Excess Stroke Mortality in Rural Areas in the United States. *Stroke* 2017; **48**(7): 1773-8.
33. Howard VJ, Kleindorfer DO, Judd SE, et al. Disparities in stroke incidence contributing to disparities in stroke mortality. *Annals of Neurology* 2011; **69(4)**: 619-27.
34. Howard VJ, McClure LA, Kleindorfer DO, et al. Neighborhood socioeconomic index and stroke incidence in a national cohort of blacks and whites. *Neurology* 2016; **87(22)**: 2340-7.
35. Huang K, Liang F, Yang X, et al. Long term exposure to ambient fine particulate matter and incidence of stroke: Prospective cohort study from the China-PAR project. *The BMJ* 2019; **367 (no pagination)**.

36. Huh JY, Ross GW, Chen R, et al. Total and differential white blood cell counts in late life predict 8-year incident stroke: the Honolulu Heart Program. *J Am Geriatr Soc* 2015; **63**(3): 439-46.
37. Imano H, Kitamura A, Sato S, et al. Trends for blood pressure and its contribution to stroke incidence in the middle-aged Japanese population: The Circulatory Risk in Communities Study (CIRCS). *Stroke* 2009; **40(5)**: 1571-7.
38. Isiozor NM, Kunutsor SK, Voutilainen A, Kauhanen J, Laukkanen JA. Life's Simple 7 and the risk of stroke in Finnish men: A prospective cohort study. *Prev Med* 2021; **153**: 106858.
39. Jackson CA, Jones M, Mishra GD. Educational and homeownership inequalities in stroke incidence: a population-based longitudinal study of mid-aged women. *European journal of public health* 2014; **24(2)**: 231-6.
40. Jacobsen BK, Oda K, Knutsen SF, Fraser GE. Age at menarche, total mortality and mortality from ischaemic heart disease and stroke: The Adventist Health Study, 1976 - 88. *International Journal of Epidemiology* 2009; **38(1)**: 245-52.
41. Jiang G, Li W, Wang D, Shen C, Ji Y, Zheng W. Epidemiological transition and distribution of stroke incidence in Tianjin, China, 1988-2010. *Public Health* 2016; **131**: 11-9.
42. Jiang S, Li J, Zhang Y, et al. Methylenetetrahydrofolate reductase C677T polymorphism, hypertension and risk of stroke: a prospective, nested case-control study. *Int J Neurosci* 2017; **127**(3): 253-60.
43. Joseph JJ, Echouffo-Tcheugui JB, Kalyani RR, et al. Aldosterone, Renin, Cardiovascular Events, and All-Cause Mortality Among African Americans: The Jackson Heart Study. *JACC: Heart Failure* 2017; **5(9)**: 642-51.
44. Kaddumukasa M, Mugenyi L, Kaddumukasa MN, et al. Prevalence and incidence of neurological disorders among adult Ugandans in rural and urban Mukono district; a cross-sectional study. *BMC Neurology* 2016; **16(1) (no pagination)**.
45. Kamin Mukaz D, Dawson E, Howard VJ, et al. Rural/urban differences in the prevalence of stroke risk factors: A cross-sectional analysis from the REGARDS study. *The Journal of rural health : official journal of the American Rural Health Association and the National Rural Health Care Association* 2022; **38(3)**: 668-73.
46. Karantali E, Vemmos K, Tsampalas E, et al. Temporal trends in stroke incidence and case-fatality rates in Arcadia, Greece: A sequential, prospective, population-based study. *International Journal of Stroke* 2022; **17(1)**: 37-47.
47. Kaup AO, Dos Santos BFC, Victor ES, et al. Georeferencing deaths from stroke in Sao Paulo: An intra-city stroke belt? *International Journal of Stroke* 2015; **10(A100)**: 69-74.
48. Ke J, Liu X, Ruan X, et al. Short sleep duration associated with the incidence of cardio-cerebral vascular disease: a prospective cohort study in Shanghai, China. *BMC cardiovascular disorders* 2023; **23(1)**: 177.
49. Khan SS, Ning H, Wilkins JT, et al. Association of body mass index with lifetime risk of cardiovascular disease and compression of morbidity. *JAMA Cardiology* 2018; **3(4)**: 280-7.
50. Khawaja O, Maziarz M, Biggs ML, et al. Plasma free fatty acids and risk of stroke in the Cardiovascular Health Study. *International Journal of Stroke* 2014; **9(7)**: 917-20.
51. Kim M, Yun SM, Jeong J, Jo C, Koh YH. Association between blood lead level and risk of stroke in Korean adults: a cross-sectional study in the Korea National Health and Nutrition Examination Survey 2008-2013. *BMJ open* 2020; **10(9)**: e035725.
52. Kitamura A, Sato S, Kiyama M, et al. Trends in the incidence of coronary heart disease and stroke and their risk factors in Japan, 1964 to 2003: the Akita-Osaka study. *J Am Coll Cardiol* 2008; **52**(1): 71-9.
53. Koh AS, Pan A, Wang R, et al. The association between dietary omega-3 fatty acids and cardiovascular death: The Singapore Chinese Health Study. *European Journal of Preventive Cardiology* 2015; **22(3)**: 364-72.
54. Korda RJ, Soga K, Joshy G, et al. Socioeconomic variation in incidence of primary and secondary major cardiovascular disease events: an Australian population-based prospective cohort study. *International Journal for Equity in Health* 2016; **15(1)**: 1-10.
55. Koton S, Sang Y, Schneider ALC, Rosamond WD, Gottesman RF, Coresh J. Trends in Stroke Incidence Rates in Older US Adults: An Update from the Atherosclerosis Risk in Communities (ARIC) Cohort Study. *JAMA Neurology* 2020; **77(1)**: 109-13.
56. LaCroix AZ, Bellettiere J, Rillamas-Sun E, et al. Association of Light Physical Activity Measured by Accelerometry and Incidence of Coronary Heart Disease and Cardiovascular Disease in Older Women. *JAMA network open* 2019; **2(3)**: e190419.
57. Lakshman R, Forouhi NG, Sharp SJ, et al. Early age at menarche associated with cardiovascular disease and mortality. *Journal of Clinical Endocrinology and Metabolism* 2009; **94(12)**: 4953-60.
58. Lear SA, Hu W, Rangarajan S, et al. The effect of physical activity on mortality and cardiovascular disease in 130 000 people from 17 high-income, middle-income, and low-income countries: the PURE study. *Lancet* 2017; **390**(10113): 2643-54.
59. Leong T, Zylberstein D, Graham I, et al. Asymmetric dimethylarginine independently predicts fatal and nonfatal myocardial infarction and stroke in women: 24-Year follow-up of the population study of women in Gothenburg. *Arteriosclerosis, Thrombosis, and Vascular Biology* 2008; **28(5)**: 961-7.

60. Li L, Scott CA, Rothwell PM. Trends in Stroke Incidence in High-Income Countries in the 21st Century: Population-Based Study and Systematic Review. *Stroke* 2020: 1372-80.
61. Li S, Lear SA, Rangarajan S, et al. Association of Sitting Time with Mortality and Cardiovascular Events in High-Income, Middle-Income, and Low-Income Countries. *JAMA Cardiology* 2022; **7(8)**: 796-807.
62. Li T, Li G, Guo X, Li Z, Yang J, Sun Y. Predictive value of echocardiographic left atrial size for incident stroke and stroke cause mortality: A population-based study. *BMJ Open* 2021; **11(3) (no pagination)**.
63. Li Z, Yang Q, Zhang X, et al. Analysis of screening results of high-risk population of stroke in Northern Henan Province. *medRxiv* 2022; **18**.
64. Liu F, Yang X, Li J, et al. Association of fasting glucose levels with incident atherosclerotic cardiovascular disease: An 8-year follow-up study in a Chinese population. *Journal of Diabetes* 2017; **9(1)**: 14-23.
65. Liu J, Lin Q, Gao Y, et al. Health care reform and stroke prognosis in low-income Chinese populations from 1992 to 2018. *Journal of global health* 2021; **11**: 08002.
66. Liu J, Yang Q, Zhang X, et al. A Sharp Decline in Burden of Stroke in Rural China During COVID-19 Pandemic. *Frontiers in Neurology* 2020; **11 (no pagination)**.
67. López-Suárez A, Bascuñana-Quirell A, Elvira-González J, Beltrán-Robles M, Aboza-Lobatón A, Solís-Díaz R. Community-based incidence rate of cardiovascular disease and mortality in 50-75 year old adults. *Rev Clin Esp (Barc)* 2013; **213**(6): 278-84.
68. Lou G, Li SX, Gong QH, et al. [Association between physical activity and risk of stroke among adults aged 40 years and above: a prospective cohort study]. *Zhonghua Liu Xing Bing Xue Za Zhi* 2021; **42**(6): 1030-6.
69. Ma Z, Zhang Y, Zhou C, Liu D, Gu S, Zuo H. Independent and combined effect of income and education attainment on the incidence of stroke events: a large-scale cohort study from rural communities in China. *Neurological Research* 2023.
70. Maredza M, Bertram MY, Tollman SM. Disease burden of stroke in rural South Africa: an estimate of incidence, mortality and disability adjusted life years. *BMC Neurol* 2015; **15**: 54.
71. Mateen FJ, Carone M, Alam N, Streatfield PK, Black RE. A population-based case-control study of 1250 stroke deaths in rural Bangladesh. *European Journal of Neurology* 2012; **19(7)**: 999-1006.
72. McDonnell MN, Hillier SL, Judd SE, Yuan Y, Hooker SP, Howard VJ. Association between television viewing time and risk of incident stroke in a general population: Results from the REGARDS study. *Preventive Medicine* 2016; **87**: 1-5.
73. Mihara A, Ohara T, Hata J, et al. Association between serum glycated albumin and risk of cardiovascular disease in a Japanese community: The Hisayama Study. *Atherosclerosis* 2020; **311**: 52-9.
74. Moon KA, Guallar E, Umans JG, et al. Association between exposure to low to moderate arsenic levels and incident cardiovascular disease. A prospective cohort study. *Ann Intern Med* 2013; **159**(10): 649-59.
75. Morovatdar N, Thrift AG, Stranges S, et al. Socioeconomic Status and Long-Term Stroke Mortality, Recurrence and Disability in Iran: The Mashhad Stroke Incidence Study. *Neuroepidemiology* 2019; **53(1-2)**: 27-31.
76. Nagayoshi M, Everson-Rose SA, Iso H, Mosley TH, Rose KM, Lutsey PL. Social network, social support, and risk of incident stroke: Atherosclerosis Risk in Communities study. *Stroke; a journal of cerebral circulation* 2014; **45(10)**: 2868-73.
77. Nagayoshi M, Tanigawa T, Yamagishi K, et al. Self-reported snoring frequency and incidence of cardiovascular disease: The circulatory risk in communities study (CIRCS). *Journal of Epidemiology* 2012; **22(4)**: 295-301.
78. Ninomiya T, Nagata M, Hata J, et al. Association between ratio of serum eicosapentaenoic acid to arachidonic acid and risk of cardiovascular disease: The Hisayama Study. *Atherosclerosis* 2013; **231(2)**: 261-7.
79. Osypuk TL, Ehntholt A, Moon JR, Gilsanz P, Glymour MM. Neighborhood differences in post-stroke mortality. *Circulation: Cardiovascular Quality and Outcomes* 2017; **10(2) (no pagination)**.
80. Panagiotakos DB, Pitsavos C, Chrysohoou C, et al. The effect of clinical characteristics and dietary habits on the relationship between education status and 5-year incidence of cardiovascular disease: The ATTICA study. *European Journal of Nutrition* 2008; **47(5)**: 258-65.
81. Pandian JD, Singh G, Bansal R, et al. Establishment of population-based stroke registry in Ludhiana City, Northwest India: Feasibility and methodology. *Neuroepidemiology* 2015; **44(2)**: 69-77.
82. Paterson KE, Myint PK, Jennings A, et al. Mediterranean Diet Reduces Risk of Incident Stroke in a Population With Varying Cardiovascular Disease Risk Profiles. *Stroke* 2018; **49**(10): 2415-20.
83. Perna L, Schottker B, Holleczek B, Brenner H. Serum 25-Hydroxyvitamin D and Incidence of Fatal and Nonfatal Cardiovascular Events: A Prospective Study With Repeated Measurements. *Journal of Clinical Endocrinology and Metabolism* 2013; **98(12)**: 4908-15.
84. Qi W, Ma J, Guan T, et al. Risk factors for incident stroke and its subtypes in China: A prospective study. *Journal of the American Heart Association* 2020; **9(21) (no pagination)**.
85. Radisauskas R, Malinauskiene V, Milinaviciene E, et al. Trends in the attack rates, incidence, and mortality of stroke during 1986-2012: Data of Kaunas (Lithuania) stroke registry. *PLoS ONE* 2016; **11(4) (no pagination)**.

86. Rajan KB, Aggarwal NT, Wilson RS, Everson-Rose SA, Evans DA. Association of cognitive functioning, incident stroke, and mortality in older adults. *Stroke* 2014; **45(9)**: 2563-7.
87. Rajan KB, Schneider JA, Aggarwal NT, Wilson RS, Everson-Rose SA, Evans DA. Racial Differences in Cognitive Function and Risk of Incident Stroke. *J Stroke Cerebrovasc Dis* 2015; **24**(12): 2854-9.
88. Renoux C, Coulombe J, Li L, Ganesh A, Silver L, Rothwell PM. Confounding by pre-morbid functional status in studies of apparent sex differences in severity and outcome of stroke. *Stroke* 2017; **48(10)**: 2731-8.
89. Rodriguez-Colon SM, Mo J, Duan Y, et al. Metabolic syndrome clusters and the risk of incident stroke: The atherosclerosis risk in communities (ARIC) study. *Stroke* 2009; **40(1)**: 200-5.
90. Rosen DM, Kundel V, Rueschman M, et al. Self-reported snoring and incident cardiovascular disease events: results from the Jackson Heart Study. *Sleep Breath* 2019; **23**(3): 777-84.
91. Saber H, Himali JJ, Shoamanesh A, et al. Serum leptin levels and the risk of stroke: The framingham study. *Stroke* 2015; **46(10)**: 2881-5.
92. Santosa A, Rosengren A, Ramasundarahettige C, et al. Psychosocial Risk Factors and Cardiovascular Disease and Death in a Population-Based Cohort from 21 Low-, Middle-, and High-Income Countries. *JAMA Network Open* 2021; **4(12) (no pagination)**.
93. Sarrafzadegan N, Talaei M, Sadeghi M, et al. The Isfahan cohort study: Rationale, methods and main findings. *Journal of Human Hypertension* 2011; **25(9)**: 545-53.
94. Sealy-Jefferson S, Wing JJ, Sanchez BN, et al. Age- and ethnic-specific sex differences in stroke risk. *Gender Medicine* 2012; **9(2)**: 121-8.
95. Seven E, Husemoen LLN, Sehested TSG, et al. Adipocytokines, C-Reactive protein, and cardiovascular disease: A population-based prospective study. *PLoS ONE* 2015; **10(6) (no pagination)**.
96. Shirai K, Iso H, Ohira T, et al. Perceived level of life enjoyment and risks of cardiovascular disease incidence and mortality. The Japan public health center-based study. *Circulation* 2009; **120(11)**: 956-63.
97. Sienkiewicz-Jarosz H, Gluszkiewicz M, Pniewski J, et al. Incidence and case fatality rates of first-ever stroke - comparison of data from two prospective population-based studies conducted in warsaw. *Neurologia i Neurochirurgia Polska* 2011; **45(3)**: 207-12.
98. Skaaby T, Husemoen LLN, Thuesen BH, Jeppesen J, Linneberg A. The association of atopy with incidence of ischemic heart disease, stroke, and diabetes. *Endocrine* 2015; **48(2)**: 541-50.
99. Sluyter JD, Camargo CA, Lowe A, Scragg RKR. Pulse rate variability predicts atrial fibrillation and cerebrovascular events in a large, population-based cohort. *International Journal of Cardiology* 2019; **275**: 83-8.
100. Smeeton NC, Corbin DOC, Hennis AJM, et al. A comparison of outcome for stroke patients in Barbados and South London. *International Journal of Stroke* 2011; **6(2)**: 112-7.
101. Soleimani A, Pourmoghaddas A, Sadeghi M, et al. Risk and Age of Cardiovascular Event in Women with Metabolic Syndrome: Menopause Age in Focus. *Metabolic Syndrome and Related Disorders* 2018; **16(3)**: 127-34.
102. Song QR, Liu SL, Ling QH, et al. Severity of Nonalcoholic Fatty Liver Disease is Associated With Cardiovascular Outcomes in Patients With Prehypertension or Hypertension: A Community-Based Cohort Study. *Front Endocrinol (Lausanne)* 2022; **13**: 942647.
103. Sozener CB, Lisabeth LD, Shafie-Khorassani F, et al. Trends in Stroke Recurrence in Mexican Americans and Non-Hispanic Whites. *Stroke* 2020: STROKEAHA120029376.
104. Stahovskaya LV, Klochikhina OA, Bogatyreva MD, Koshel VI, Mozheiko RA, Strakhov OA. Comparison of the incidence rate of stroke in the different regions of Russian federation (population-based register of stroke, 2009-2014). [Russian]. *Medical News of North Caucasus* 2019; **14(1)**: 64-8.
105. Stulberg EL, Twardzik E, Kim S, et al. Association of Neighborhood Socioeconomic Status With Outcomes in Patients Surviving Stroke. *Neurology* 2021; **96**(21): e2599-e610.
106. Sun C, Klein R, Wong TY. Age-related Macular Degeneration and Risk of Coronary Heart Disease and Stroke: The Cardiovascular Health Study. *Ophthalmology* 2009; **116(10)**: 1913-9.
107. Surtees PG, Wainwright NW, Luben RN, Wareham NJ, Bingham SA, Khaw KT. Psychological distress, major depressive disorder, and risk of stroke. *Neurology* 2008; **70(10)**: 788-94.
108. Tamasauskiene L, Rastenyte D, Radisauskas R, et al. Relationship of meteorological factors and acute stroke events in Kaunas (Lithuania) in 2000-2010. *Environmental science and pollution research international* 2017; **24(10)**: 9286-93.
109. Tang M, Zhao Q, Yi K, et al. Association between Metabolic Phenotypes of Body Fatness and Incident Stroke: A Prospective Cohort Study of Chinese Community Residents. *Nutrients* 2022; **14(24) (no pagination)**.
110. Tse LA, Fang XH, Wang WZ, Qiu H, Yu ITS. Incidence of ischaemic and haemorrhagic stroke and the association with smoking and smoking cessation: A 10-year multicentre prospective study in China. *Public Health* 2012; **126(11)**: 960-6.
111. Tu JV, Chu A, Maclagan L, et al. Regional variations in ambulatory care and incidence of cardiovascular events. *Cmaj* 2017; **189(13)**: E494-E501.

112. Usui T, Nagata M, Hata J, et al. Serum non-high-density lipoprotein cholesterol and risk of cardiovascular disease in community dwellers with chronic kidney disease: The hisayama study. *Journal of Atherosclerosis and Thrombosis* 2017; **24(7)**: 706-15.
113. Van Sloten TT, Tafflet M, Perier MC, et al. Association of Change in Cardiovascular Risk Factors with Incident Cardiovascular Events. *JAMA - Journal of the American Medical Association* 2018; **320(17)**: 1793-804.
114. Vissers LET, Waller MA, van der Schouw YT, et al. The relationship between the dietary inflammatory index and risk of total cardiovascular disease, ischemic heart disease and cerebrovascular disease: Findings from an Australian population-based prospective cohort study of women. *Atherosclerosis* 2016; **253**: 164-70.
115. Walli-Attaei M, Joseph P, Rosengren A, et al. Variations between women and men in risk factors, treatments, cardiovascular disease incidence, and death in 27 high-income, middle-income, and low-income countries (PURE): a prospective cohort study. *The Lancet* 2020; **396(10244)**: 97-109.
116. Wang J, Li J, Liu F, et al. Genetic Predisposition, Fruit Intake and Incident Stroke: A Prospective Chinese Cohort Study. *Nutrients* 2022; **14(23) (no pagination)**.
117. Wang Q, Zhang L, Li Y, Tang X, Yao Y, Fang Q. Development of stroke predictive model in community-dwelling population: A longitudinal cohort study in Southeast China. *Front Aging Neurosci* 2022; **14**: 1036215.
118. Wannamethee SG, Welsh P, Lennon L, Papacosta O, Whincup PH, Sattar N. Copeptin and the risk of incident stroke, CHD and cardiovascular mortality in older men with and without diabetes: The British Regional Heart Study. *Diabetologia* 2016; **59(9)**: 1904-12.
119. Wieberdink RG, Ikram MA, Hofman A, Koudstaal PJ, Breteler MM. Trends in stroke incidence rates and stroke risk factors in Rotterdam, the Netherlands from 1990 to 2008. *Eur J Epidemiol* 2012; **27**(4): 287-95.
120. Willey JZ, Moon YP, Kahn E, et al. Population attributable risks of hypertension and diabetes for cardiovascular disease and stroke in the Northern Manhattan study. *Journal of the American Heart Association* 2014; **3(5) (no pagination)**.
121. Wolfe CDA, Crichton SL, Heuschmann PU, et al. Estimates of Outcomes Up to Ten Years after Stroke: Analysis from the Prospective South London Stroke Register. *PLoS Medicine* 2011; **8(5) (no pagination)**.
122. Wouts L, Voshaar RCO, Bremmer MA, Buitelaar JK, Penninx BWJH, Beekman ATF. Cardiac disease, depressive symptoms, and incident stroke in an elderly population. *Archives of General Psychiatry* 2008; **65(5)**: 596-602.
123. Wu S, Song Y, Chen S, et al. Blood Pressure Classification of 2017 Associated with Cardiovascular Disease and Mortality in Young Chinese Adults. *Hypertension* 2020; **76(1)**: 251-8.
124. Xiao J, Chen L, Melander O, et al. Circulating Vimentin Is Associated with Future Incidence of Stroke in a Population-Based Cohort Study. *Stroke* 2021: 937-44.
125. Xu M, Amarilla Vallejo A, Cantalapiedra Calvete C, et al. Stroke Outcomes in Women: A Population-Based Cohort Study. *Stroke* 2022; **53(10)**: 3072-81.
126. Xue XD, Jiang GH, Li W, et al. [Epidemiological characteristics of hemorrhagic stroke among residents in Tianjin from 1999 to 2018]. *Zhonghua Liu Xing Bing Xue Za Zhi* 2021; **42**(8): 1460-5.
127. Yan B, Jin X, Li R, et al. Association of daytime napping with incident stroke in middle-aged and older adults: a large community-based study. *European Journal of Neurology* 2020; **27(6)**: 1028-34.
128. Yatsuya H, Iso H, Yamagishi K, et al. Development of a point-based prediction model for the incidence of total stroke: Japan public health center study. *Stroke* 2013; **44(5)**: 1295-302.
129. Yilmaz P, Ikram MK, Niessen WJ, Ikram MA, Vernooij MW. Practical small vessel disease score relates to stroke, dementia, and death: The Rotterdam study. *Stroke* 2018; **49(12)**: 2857-65.
130. Zhang J, Du R, Peng K, et al. Serum lipoprotein (a) is associated with increased risk of stroke in Chinese adults: A prospective study. *Atherosclerosis* 2019; **289**: 8-13.
131. Zheng L, Xie Y, Zheng J, et al. Associations between ideal blood pressure based on different BMI categories and stroke incidence. *Journal of Hypertension* 2020; **38(7)**: 1271-7.
132. Zhou H, Ding X, Yang Q, et al. Associations of Hypertriglyceridemia Onset Age With Cardiovascular Disease and All-Cause Mortality in Adults: A Cohort Study. *J Am Heart Assoc* 2022; **11**(20): e026632.

vi. Case finding based solely on ICD-codes (n = 93)

1. Ariss RW, Minhas AMK, Lang J, et al. Urban-Rural Trends in Young Stroke-Related Mortality in the United States, 1999-2019. *J Am Coll Cardiol* 2022; **80**(4): 466-8.
2. Barlas G, Luben RL, Neal SR, Wareham NJ, Khaw KT, Myint PK. Self-Reported Fatigue Predicts Incident Stroke in a General Population: EPIC-Norfolk Prospective Population-Based Study. *Stroke* 2020: 1077-84.
3. Behr S, Andersohn F, Garbe E. Risk of intracerebral hemorrhage associated with phenprocoumon exposure: A nested case-control study in a large population-based German database. *Pharmacoepidemiology and Drug Safety* 2010; **19(7)**: 722-30.
4. Bonzini M, Ferrario M, Bert L, et al. Temporal trends in ischemic and hemorrhagic strokes in Northern Italy: Results from the cardiovascular monitoring unit in Northern Italy population-based register, 1998-2004. *Neuroepidemiology* 2012; **39(1)**: 35-42.

5. Carrington SJ, Romero-Alvarez D, Coral-Almeida M, Vela A, Henriquez-Trujillo AR, Mascialino G. Ethnodemographic characterization of stroke incidence and burden of disease in hospital discharge records in Ecuador. *Frontiers in Neurology* 2023; **14 (no pagination)**.

6. Cayuela A, Cayuela L, Ortega Belmonte MJ, Rodríguez-Domínguez S, Escudero-Martínez I, González A. Has stroke mortality stopped declining in Spain? *Neurologia (Engl Ed)* 2019.

7. Che B, Zhong C, Zhang R, Wang M, Zhang Y, Han L. Multivitamin/mineral supplementation and the risk of cardiovascular disease: a large prospective study using UK Biobank data. *European Journal of Nutrition* 2022; **61(6)**: 2909-17.

8. Chien TY, Ting HW, Chan CL, et al. Does the short-term effect of air pollution influence the incidence of spontaneous intracerebral hemorrhage in different patient groups? Big data analysis in Taiwan. *International Journal of Environmental Research and Public Health* 2017; **14(12) (no pagination)**.

9. Chung JW, Lee SR, Choi EK, et al. Cumulative Alcohol Consumption Burden and the Risk of Stroke in Young Adults: A Nationwide Population-Based Study. *Neurology* 2023; **100(5)**: E505-E15.

10. Cruz-Góngora V, Chiquete E, Gómez-Dantés H, Cahuana-Hurtado L, Cantú-Brito C. Trends in the burden of stroke in Mexico: A national and subnational analysis of the global burden of disease 1990-2019. *Lancet Reg Health Am* 2022; **10**: 100204.

11. da Silva Paiva L, Schoueri JHM, de Alcantara Sousa LV, et al. Regional differences in the temporal evolution of stroke: a population-based study of Brazil according to sex in individuals aged 15-49 years between 1997 and 2012. *BMC research notes* 2018; **11(1)**: 326.

12. de Goede J, Verschuren WMM, Boer JMA, Kromhout D, Geleijnse JM. Alpha-linolenic acid intake and 10-year incidence of coronary heart disease and stroke in 20,000 middle-aged men and women in the Netherlands. *PLoS ONE* 2011; **6(3) (no pagination)**.

13. DeLago AJ, Singh H, Jani C, et al. An observational epidemiological study to analyze intracerebral hemorrhage across the United States: Incidence and mortality trends from 1990 to 2017. *Journal of Stroke and Cerebrovascular Diseases* 2022; **31(4) (no pagination)**.

14. Fadel M, Sembajwe G, Tripodi D, et al. Association between reported work in cold environments and stroke occurrence in the CONSTANCES cohort: a prospective study. *BMJ Open* 2022; **12(7) (no pagination)**.

15. Falkstedt D, Wolff V, Allebeck P, Hemmingsson T, Danielsson AK. Cannabis, Tobacco, Alcohol Use, and the Risk of Early Stroke: A Population-Based Cohort Study of 45 000 Swedish Men. *Stroke* 2017; **48(2)**: 265-70.

16. Fan M, Lv J, Yu C, et al. Association Between Active Commuting and Incident Cardiovascular Diseases in Chinese: A Prospective Cohort Study. *Journal of the American Heart Association* 2019; **8(20) (no pagination)**.

17. Flynn RWV, MacDonald TM, Murray GD, Ferguson C, Shah K, Doney ASF. The Tayside stroke cohort: Exploiting advanced regional medical informatics to create a region-wide database for studying the pharmacoepidemiology of stroke. *Pharmacoepidemiology and Drug Safety* 2010; **19(7)**: 737-44.

18. Gattellari M, Goumas C, Jalaludin B, Worthington J. Measuring stroke outcomes for 74,501 patients using linked administrative data: System-wide estimates and validation of 'home-time' as a surrogate measure of functional status. *International journal of clinical practice* 2020: e13484.

19. Gattellari M, Goumas C, Worthington J. Declining rates of fatal and nonfatal intracerebral hemorrhage: Epidemiological trends in Australia. *Journal of the American Heart Association* 2014; **3(6) (no pagination)**.

20. Goldacre MJ, Duncan M, Griffith M, Rothwell PM. Mortality rates for stroke in England from 1979 to 2004: Trends, diagnostic precision, and artifacts. *Stroke* 2008; **39(8)**: 2197-203.

21. Gong W, Wei X, Liang Y, et al. Urban and rural differences of acute cardiovascular disease events: A study from the population-based real-time surveillance system in Zhejiang, China in 2012. *PLoS ONE* 2016; **11(11) (no pagination)**.

22. Gulsvik AK, Gulsvik A, Skovlund E, et al. The association between lung function and fatal stroke in a community followed for 4 decades. *Journal of Epidemiology and Community Health* 2012; **66(11)**: 1030-6.

23. Gunarathne A, Patel JV, Potluri R, Gill PS, Hughes EA, Lip GY. Secular trends in the cardiovascular risk profile and mortality of stroke admissions in an inner city, multiethnic population in the United Kingdom (1997-2005). *J Hum Hypertens* 2008; **22**(1): 18-23.

24. Hall EW, Vaughan AS, Ritchey MD, Schieb L, Casper M. Stagnating national declines in stroke mortality mask widespread county-level increases, 2010-2016. *Stroke* 2019; **50(12)**: 3355-9.

25. Hayes S, Forbes JF, Celis-Morales C, et al. Association between Walking Pace and Stroke Incidence: Findings from the UK Biobank Prospective Cohort Study. *Stroke* 2020: 1388-95.

26. Ho AFW, Lim MJR, Zheng H, et al. Association of ambient air pollution with risk of hemorrhagic stroke: A time-stratified case crossover analysis of the Singapore stroke registry. *International journal of hygiene and environmental health* 2022; **240**: 113908.

27. Hunt BR, Deot D, Whitman S. Stroke mortality rates vary in local communities in a metropolitan area: Racial and spatial disparities and correlates. *Stroke* 2014; **45(7)**: 2059-65.

28. Ilic I, Ilic M, Sipetic Grujicic S. Trends in cerebrovascular diseases mortality in Serbia, 1997-2016: A nationwide descriptive study. *BMJ Open* 2019; **9(2) (no pagination)**.

29. Jackson CA, Sudlow CLM, Mishra GD. Psychological Distress and Risk of Myocardial Infarction and Stroke in the 45 and Up Study. *Circulation* 2018; **Cardiovascular quality and outcomes. 11(9)**: e004500.

30. Jackson CA, Sudlow CLM, Mishra GD. Education, sex and risk of stroke: A prospective cohort study in New South Wales, Australia. *BMJ Open* 2018; **8(9) (no pagination)**.

31. Janzi S, Ramne S, González-Padilla E, Johnson L, Sonestedt E. Associations Between Added Sugar Intake and Risk of Four Different Cardiovascular Diseases in a Swedish Population-Based Prospective Cohort Study. *Front Nutr* 2020; **7**: 603653.

32. Jeppesen J, Hansen TW, Olsen MH, et al. C-reactive protein, insulin resistance and risk of cardiovascular disease: A population-based study. *European Journal of Cardiovascular Prevention and Rehabilitation* 2008; **15(5)**: 594-8.

33. Jhuo SJ, Tsai WC, Lin TH, Voon WC, Lai WT, Sheu SH. Statin dose and the risk of intracerebral hemorrhage: A population-based longitudinal study in Taiwan. *Acta Cardiologica Sinica* 2016; **32(1)**: 23-30.

34. Joundi RA, Patten SB, Williams JVA, Smith EE. Association Between Excess Leisure Sedentary Time and Risk of Stroke in Young Individuals. *Stroke* 2021: STROKEAHA121034985.

35. Joundi RA, Patten SB, Williams JVA, Smith EE. Vascular risk factors and stroke risk across the life span: A population-representative study of half a million people. *International Journal of Stroke* 2022; **17(9)**: 1021-9.

36. Joundi RA, Patten SB, Williams JVA, Smith EE. Association between Excess Sleep Duration and Risk of Stroke: A Population-Based Study. *Canadian Journal of Neurological Sciences* 2023; **50(1)**: 17-22.

37. Joundi RA, Smith EE, Yu AYX, Rashid M, Fang J, Kapral MK. Temporal Trends in Case Fatality, Discharge Destination, and Admission to Long-term Care After Acute Stroke. *Neurology* 2021; **96(16)**: e2037-e47.

38. Julin B, Bergkvist C, Wolk A, Akesson A. Cadmium in diet and risk of cardiovascular disease in women. *Epidemiology* 2013; **24(6)**: 880-5.

39. Kalediene R, Rastenyte D. Trends and regional inequalities in mortality from stroke in the context of health care reform in Lithuania. *Medicina (Kaunas, Lithuania)* 2016; **52(4)**: 244-9.

40. Kaluza J, Wolk A, Larsson SC. Heme iron intake and risk of stroke: A prospective study of men. *Stroke* 2013; **44(2)**: 334-9.

41. Kawachi T, Wada K, Nakamura K, et al. Sleep Duration and the Risk of Mortality From Stroke in Japan: The Takayama Cohort Study. *Journal of epidemiology* 2016; **26(3)**: 123-30.

42. Khan SU, Kalra A, Yedlapati SH, et al. Stroke-related mortality in the united states-mexico border area of the united states, 1999 to 2018. *Journal of the American Heart Association* 2021; **10(13) (no pagination)**.

43. Kim HC, Oh SM, Pan WH, et al. Association between alanine aminotransferase and intracerebral hemorrhage in east Asian populations. *Neuroepidemiology* 2013; **41(2)**: 131-8.

44. Kim MK, Han K, Kim HS, et al. Cholesterol variability and the risk of mortality, myocardial infarction, and stroke: A nationwide population-based study. *European Heart Journal* 2017; **38(48)**: 3560-6.

45. Kivela M, Rissanen I, Kajantie E, et al. Pregnancy Risk Factors as Predictors of Offspring Cerebrovascular Disease: The Northern Finland Birth Cohort Study 1966. *Stroke* 2021: 1347-54.

46. Kuhn A, van der Giet M, Kuhlmann MK, et al. Kidney Function as Risk Factor and Predictor of Cardiovascular Outcomes and Mortality Among Older Adults. *American journal of kidney diseases : the official journal of the National Kidney Foundation* 2020; **13**.

47. Larsson SC, Virtamo J, Wolk A. Fish consumption and risk of stroke in Swedish women. *American Journal of Clinical Nutrition* 2011; **93(3)**: 487-93.

48. Larsson SC, Virtamo J, Wolk A. Dietary protein intake and risk of stroke in women. *Atherosclerosis* 2012; **224(1)**: 247-51.

49. Lee S, Lee H, Kim HS, Koh SB. Incidence, risk factors, and prediction of myocardial infarction and stroke in farmers: A Korean nationwide population-based study. *Journal of Preventive Medicine and Public Health* 2020; **53(5)**: 313-22.

50. Levine DA, Davydow DS, Hough CL, Langa KM, Rogers MA, Iwashyna TJ. Functional disability and cognitive impairment after hospitalization for myocardial infarction and stroke. *Circ Cardiovasc Qual Outcomes* 2014; **7**(6): 863-71.

51. Levy M, Chen Y, Clarke R, et al. Socioeconomic differences in health-care use and outcomes for stroke and ischaemic heart disease in China during 2009-16: a prospective cohort study of 0·5 million adults. *Lancet Glob Health* 2020; **8**(4): e591-e602.

52. Li J, Li B, Zhang F, Sun Y. Urban and rural stroke mortality rates in China between 1988 and 2013: An age-period-cohort analysis. *Journal of International Medical Research* 2017; **45(2)**: 680-90.

53. Lindvall E, Franzon K, Lundstrom E, Kilander L. The impact of stroke on the ability to live an independent life at old age: a community-based cohort study of Swedish men. *BMC geriatrics* 2023; **23(1)**: 126.

54. Mansur AP, Favarato D, Avakian SD, Ramires JAF. Trends in ischemic heart disease and stroke death ratios in Brazilian women and men. *Clinics* 2010; **65(11)**: 1143-7.

55. Marshall NS, Wong KKH, Cullen SRJ, Knuiman MW, Grunstein RR. Snoring is not associated with all-cause mortality, incident cardiovascular disease, or stroke in the busselton health study. *Sleep* 2012; **35(9)**: 1235-40.

56. Marshall NS, Wong KKH, Cullen SRJ, Knuiman MW, Grunstein RR. Sleep apnea and 20-year follow-up for all-cause mortality, stroke, and cancer incidence and mortality in the Busselton health study cohort. *Journal of Clinical Sleep Medicine* 2014; **10(4)**: 355-62.

57. Mkoma GF, Johnsen SP, Iversen HK, Andersen G, Norredam M. Incidence of stroke, transient ischaemic attack and determinants of poststroke mortality among immigrants in Denmark, 2004–2018: a population-based cohort study. *BMJ Open* 2021; **11**(10): e049347.

58. Myint PK, Luben RN, Wareham NJ, Bingham SA, Khaw KT. Combined effect of health behaviours and risk of first ever stroke in 20,040 men and women over 11 years' follow-up in Norfolk cohort of European Prospective Investigation of Cancer (EPIC Norfolk): prospective population study. *Bmj* 2009; **338**: b349.

59. Myint PK, Luben RN, Welch AA, Bingham SA, Wareham NJ, Khaw KT. Plasma vitamin C concentrations predict risk of incident stroke over 10 y in 20 649 participants of the European Prospective Investigation into Cancer Norfolk prospective population study. *Am J Clin Nutr* 2008; **87**(1): 64-9.

60. Myint PK, Sinha S, Luben RN, Bingham SA, Wareham NJ, Khaw KT. Risk factors for first-ever stroke in the EPIC-Norfolk prospective population-based study. *European Journal of Cardiovascular Prevention and Rehabilitation* 2008; **15(6)**: 663-9.

61. Nagata C, Wada K, Tamura T, et al. Dietary intakes of glutamic acid and glycine are associated with stroke mortality in Japanese adults. *Journal of Nutrition* 2015; **145(4)**: 720-8.

62. Novak M, Toren K, Lappas G, et al. Occupational status and incidences of ischemic and hemorrhagic stroke in Swedish men: A population-based 35-year prospective follow-up study. *European Journal of Epidemiology* 2013; **28(8)**: 697-704.

63. Orioli R, Antonucci C, Scortichini M, et al. Exposure to residential greenness as a predictor of cause-specific mortality and stroke incidence in the rome longitudinal study. *Environmental Health Perspectives* 2019; **127(2) (no pagination)**.

64. Ortiz-Prado E, Espinosa PS, Borrero A, et al. Stroke-Related Mortality at Different Altitudes: A 17-Year Nationwide Population-Based Analysis From Ecuador. *Frontiers in Physiology* 2021; **12 (no pagination)**.

65. Oude Griep LM, Verschuren WM, Kromhout D, Ocke MC, Geleijnse JM. Variety in fruit and vegetable consumption and 10-year incidence of CHD and stroke. *Public health nutrition* 2012; **15(12)**: 2280-6.

66. Pan A, De Silva DA, Yuan JM, Koh WP. Sleep duration and risk of stroke mortality among chinese adults: Singapore chinese health study. *Stroke* 2014; **45(6)**: 1620-5.

67. Park CS, Choi EK, Han KD, et al. Association between adult height, myocardial infarction, heart failure, stroke and death: A Korean nationwide population-based study. *International Journal of Epidemiology* 2018; **47(1)**: 289-98.

68. Pedigo A, Aldrich T, Odoi A. Neighborhood disparities in stroke and myocardial infarction mortality: a GIS and spatial scan statistics approach. *BMC public health* 2011; **11**: 644.

69. Ramalle-Gomara E, Ruiz E, Serrano M, Bartulos M, Gonzalez MA, Matute B. Hospital discharges and mortality registries: 2 complementary databases for the epidemiological surveillance of stroke. *Journal of Stroke and Cerebrovascular Diseases* 2013; **22(8)**: e441-e5.

70. Rautiainen S, Larsson S, Virtamo J, Wolk A. Total antioxidant capacity of diet and risk of stroke: a population-based prospective cohort of women. *Stroke* 2012; **43**(2): 335-40.

71. Rossi M, Turati F, Lagiou P, Trichopoulos D, La Vecchia C, Trichopoulou A. Relation of dietary glycemic load with ischemic and hemorrhagic stroke: a cohort study in Greece and a meta-analysis. *Eur J Nutr* 2015; **54**(2): 215-22.

72. Rucker V, Wiedmann S, O'Flaherty M, Busch MA, Heuschmann PU. Decline in regional trends in mortality of stroke subtypes in Germany from 1998 to 2015. *Stroke* 2018; **49(11)**: 2577-83.

73. Seminog OO, Scarborough P, Wright FL, Rayner M, Goldacre MJ. Determinants of the decline in mortality from acute stroke in England: linked national database study of 795 869 adults. *Bmj* 2019; **365**: l1778.

74. Sergeev AV. Racial and rural-urban disparities in stroke mortality outside the Stroke Belt. *Ethn Dis* 2011; **21**(3): 307-13.

75. Silventoinen K, Magnusson PKE, Tynelius P, Batty GD, Rasmussen F. Association of body size and muscle strength with incidence of coronary heart disease and cerebrovascular diseases: A population-based cohort study of one million Swedish men. *International Journal of Epidemiology* 2009; **38(1)**: 110-8.

76. Son JS, Choi S, Kim K, et al. Association of Blood Pressure Classification in Korean Young Adults According to the 2017 American College of Cardiology/American Heart Association Guidelines with Subsequent Cardiovascular Disease Events. *JAMA - Journal of the American Medical Association* 2018; **320(17)**: 1783-92.

77. Soto A, Guillen-Grima F, Morales G, Munoz S, Aguinaga-Ontoso I. Trends in mortality from stroke in the European Union, 1996-2015. *European Journal of Neurology* 2021; **28(1)**: 182-91.

78. Sousa LVA, Paiva LS, Figueiredo FWS, Almeida TCC, Oliveira FR, Adami F. Trends in stroke-related mortality in the ABC region, sao Paulo, Brazil: An ecological study between 1997 and 2012. *Open Cardiovascular Medicine Journal* 2017; **11**: 111-9.
79. Staessen JA, Yang WY, Melgarejo JD, et al. Association of Office and Ambulatory Blood Pressure with Mortality and Cardiovascular Outcomes. *JAMA - Journal of the American Medical Association* 2019; **322(5)**: 409-20.
80. Stein M, Misselwitz B, Hamann GF, Scharbrodt W, Schummer DI, Oertel MF. Intracerebral hemorrhage in the very old: Future demographic trends of an aging population. []. *Stroke* 2012; **26**.
81. Tan CS, Muller-Riemenschneider F, Xian Ng SH, et al. Trends in stroke incidence and 28-day case fatality in a nationwide stroke registry of a multiethnic Asian population. *Stroke* 2015; **46(10)**: 2728-34.
82. Tektonidis TG, Akesson A, Gigante B, Wolk A, Larsson SC. A Mediterranean diet and risk of myocardial infarction, heart failure and stroke: A population-based cohort study. *Atherosclerosis* 2015; **243(1)**: 93-8.
83. Ting HW, Chien TY, Robert Lai K, et al. Differences in spontaneous intracerebral hemorrhage cases between urban and rural regions of Taiwan: Big data analytics of government open data. *International Journal of Environmental Research and Public Health* 2017; **14(12) (no pagination)**.
84. Tran J, Norton R, Conrad N, et al. Patterns and temporal trends of comorbidity among adult patients with incident cardiovascular disease in the UK between 2000 and 2014: A population-based cohort study. *PLoS Medicine* 2018; **15(3) (no pagination)**.
85. Vena AB, Cabré X, Piñol R, Molina J, Purroy F. Assessment of incidence and trends in cerebrovascular disease in the healthcare district of Lleida (Spain) in the period 2010-2014. *Neurologia (Engl Ed)* 2020.
86. Vena AB, Cabre X, Pinol R, Molina J, Purroy F. Assessment of incidence and trends in cerebrovascular disease in the healthcare district of Lleida (Spain) in the period 2010-2014. *Neurologia* 2022; **37(8)**: 631-8.
87. Vencloviene J, Radisauskas R, Kranciukaite-Butylkiniene D, Tamosiunas A, Vaiciulis V, Rastenyte D. Association between stroke occurrence and changes in atmospheric circulation. *BMC Public Health* 2021; **21**(1): 42.
88. Vyas MV, Silver FL, Austin PC, et al. Stroke Incidence by Sex across the Lifespan. *Stroke* 2021: 447-51.
89. Wang DZ, Xue XD, Zhang H, et al. [The trend of intracerebral hemorrhage mortality of the residents with different characteristics in Tianjin, China, 1999-2015]. *Zhonghua Yu Fang Yi Xue Za Zhi* 2018; **52**(4): 389-95.
90. Wennerstad KM, Sillventoinen K, Tynelius P, Bergm L, Rasmussen F. Association between intelligence and type-specific stroke: A population-based cohort study of early fatal and non-fatal stroke in one million Swedish men. *Journal of Epidemiology and Community Health* 2010; **64(10)**: 908-12.
91. Zabala A, Darsalia V, Holzmann MJ, et al. Risk of first stroke in people with type 2 diabetes and its relation to glycaemic control: A nationwide observational study. *Diabetes, Obesity and Metabolism* 2020; **22(2)**: 182-90.
92. Zhang Y, Gu S, Wang C, et al. Association between fasting blood glucose levels and stroke events: A large-scale community-based cohort study from China. *BMJ Open* 2021; **11(8) (no pagination)**.
93. Zhong Q, Qin Z, Wang X, et al. Healthy sleep pattern reduce the risk of cardiovascular disease: A 10-year prospective cohort study. *Sleep Medicine* 2023; **105**: 53-60.

vii. <80% confirmed (n = 17)

1. Clua-Espuny JL, Pinol-Moreso JL, Gil-Guillen VF, et al. Primary and secondary cardiovascular prevention results in patients with stroke: Relapse risk and associated survival (Ebrictus study). *Revista de Neurologia* 2012; **54(2)**: 81-92.
2. Hanchaiphiboolkul S, Poungvarin N, Nidhinandana S, et al. Prevalence of stroke and stroke risk factors in thailand: Thai epidemiologic stroke (TES) study. *Journal of the Medical Association of Thailand* 2011; **94(4)**: 427-36.
3. Kulesh SD, Filina NA, Frantava NM, et al. Incidence and case-fatality of stroke on the east border of the European Union: The grodno stroke study. *Stroke* 2010; **41(12)**: 2726-30.
4. Li B, Lou Y, Gu H, et al. Trends in incidence of stroke and transition of stroke subtypes in rural Tianjin China: A population-based study from 1992 to 2012. *PLoS ONE* 2015; **10(10) (no pagination)**.
5. Okon M, Adebobola NI, Julius S, et al. Stroke incidence and case fatality rate in an urban population. *Journal of Stroke and Cerebrovascular Diseases* 2015; **24(4)**: 771-7.
6. Singh S, Kate M, Samuel C, et al. Rural Stroke Surveillance and Establishment of Acute Stroke Care Pathway Using Frontline Health Workers in Rural Northwest India: The Ludhiana Experience. *Neuroepidemiology* 2021: 1-9.
7. Sridharan SE, Unnikrishnan JP, Sukumaran S, et al. Incidence, types, risk factors, and outcome of stroke in a developing country the trivandrum stroke registry. *Stroke* 2009; **40(4)**: 1212-8.
8. Thiele I, Linseisen J, Heier M, et al. Time trends in stroke incidence and in prevalence of risk factors in Southern Germany, 1989 to 2008/09. *Scientific reports* 2018; **8(1)**: 11981.
9. Walker R, Whiting D, Unwin N, et al. Stroke incidence in rural and urban Tanzania: A prospective, community-based study. *The Lancet Neurology* 2010; **9(8)**: 786-92.

10. Walker RW, Jusabani A, Aris E, Gray WK, Mitra D, Swai M. A prospective study of stroke sub-type from within an incident population in Tanzania. *South African Medical Journal* 2011; **101(5)**: 338-44.
11. Walker RW, Jusabani A, Aris E, et al. Correlates of short- and long-term case fatality within an incident stroke population in Tanzania. *S Afr Med J* 2012; **103**(2): 107-12.
12. Walker RW, Jusabani A, Aris E, et al. Stroke risk factors in an incident population in urban and rural Tanzania: A prospective, community-based, case-control study. *The Lancet Global Health* 2013; **1(5)**: e282-e8.
13. Walker RW, Jusabani A, Aris E, et al. Post-stroke case fatality within an incident population in rural Tanzania. *Journal of Neurology, Neurosurgery and Psychiatry* 2011; **82(9)**: 1001-5.
14. Wang J, Bai L, Shi M, et al. Trends in age of first-ever stroke following increased incidence and life expectancy in a low-income Chinese population. *Stroke* 2016; **47(4)**: 929-35.
15. Xia F, Yu X, Li Y, et al. Geographic variations of stroke incidence in Chinese communities: An 18-year prospective cohort study from 1997 to 2015. *Journal of Stroke* 2020; **22(3)**: 345-56.
16. Zhang X, Dai J, Li W, Yang Y. High-risk population and factors of stroke has changed among middle-aged and elderly Chinese-Evidence from 1989 to 2015. *Frontiers in public health* 2023; **11**: 1090298.
17. Zhou W, Chen R, Hopkins A, et al. Association between socioeconomic status and incident stroke in China. *Journal of epidemiology and community health* 2020; **74(6)**: 519-26.

viii. >5% missing data (n = 24)

1. Cheung EYL, Bos MJ, Leebeek FWG, et al. Variation in fibrinogen FGG and FGA genes and risk of stroke: The Rotterdam study. *Thrombosis and Haemostasis* 2008; **100(2)**: 308-13.
2. Chuang SY, Cheng HM, Bai CH, Yeh WT, Chen JR, Pan WH. Blood Pressure, Carotid Flow Pulsatility, and the Risk of Stroke: A Community-Based Study. *Stroke* 2016; **47(9)**: 2262-8.
3. Eshak ES, Iso H, Yamagishi K, et al. Rice consumption is not associated with risk of cardiovascular disease morbidity or mortality in Japanese men and women: A large population-based, prospective cohort study. *American Journal of Clinical Nutrition* 2014; **100(1)**: 199-207.
4. Glasser SP, Mosher A, Howard G, Banach M. What is the association of lipid levels and incident stroke? *International Journal of Cardiology* 2016; **220**: 890-4.
5. Judd SE, Gutiérrez OM, Newby PK, et al. Dietary patterns are associated with incident stroke and contribute to excess risk of stroke in black Americans. *Stroke* 2013; **44**(12): 3305-11.
6. Kojima G, Bell C, Abbott RD, et al. Low dietary vitamin d predicts 34-year incident stroke: The honolulu heart program. *Stroke* 2012; **43(8)**: 2163-7.
7. Leening MJG, Ferket BS, Steyerberg EW, et al. Sex differences in lifetime risk and first manifestation of cardiovascular disease: Prospective population based cohort study. *BMJ (Online)* 2014; **349 (no pagination)**.
8. Li Z, Wang A, Cai J, et al. Impact of proteinuria and glomerular filtration rate on risk of ischaemic and intracerebral hemorrhagic stroke: A result from the Kailuan study. *European Journal of Neurology* 2015; **22(2)**: 355-60.
9. Liu Y, Wang J, Zhang L, et al. Relationship between C - Reactive protein and stroke: A large prospective community based study. *PLoS ONE* 2014; **9(9) (no pagination)**.
10. Martinez-Salio A, Benito-Leon J, Diaz-Guzman J, Bermejo-Pareja F. Cerebrovascular disease incidence in central Spain (NEDICES): A population-based prospective study. *Journal of the Neurological Sciences* 2010; **298(1-2)**: 85-90.
11. Murai U, Yamagishi K, Sata M, et al. Seaweed intake and risk of cardiovascular disease: The Japan Public Health Center-based Prospective (JPHC) Study. *American Journal of Clinical Nutrition* 2019; **110(6)**: 1449-55.
12. Murakami K, Asayama K, Satoh M, et al. Home blood pressure predicts stroke incidence among older adults with impaired physical function: The Ohasama study. *Journal of Hypertension* 2017; **35(12)**: 2395-401.
13. Nakagawa K, Chen R, Greenberg SM, et al. Forkhead box O3 longevity genotype may attenuate the impact of hypertension on risk of intracerebral haemorrhage. *Journal of Hypertension* 2022; **40(11)**: 2230-5.
14. Neelamegam M, Looi I, Cheah WK, Narayanan P, Abdul Hamid AM, Ong LM. Stroke incidence in the south west district of the Penang Island, Malaysia. PEARLs: Penang acute stroke research longitudinal study. *Preventive Medicine* 2013; **57(SUPPL)**: S77-S9.
15. Okamura T, Kokubo Y, Watanabe M, et al. Low-density lipoprotein cholesterol and non-high-density lipoprotein cholesterol and the incidence of cardiovascular disease in an urban Japanese cohort study: The Suita study. *Atherosclerosis* 2009; **203(2)**: 587-92.
16. Okamura T, Kokubo Y, Watanabe M, et al. Triglycerides and non-high-density lipoprotein cholesterol and the incidence of cardiovascular disease in an urban Japanese cohort: The Suita study. *Atherosclerosis* 2010; **209(1)**: 290-4.
17. Rutten-Jacobs LC, Larsson SC, Malik R, et al. Genetic risk, incident stroke, and the benefits of adhering to a healthy lifestyle: cohort study of 306 473 UK Biobank participants. *Bmj* 2018; **363**: k4168.

18. Soderholm M, Borne Y, Hedblad B, Persson M, Engstrom G. Red cell distribution width in relation to incidence of stroke and carotid atherosclerosis: A population-based cohort study. *PLoS ONE* 2015; **10(5) (no pagination)**.
19. Sun Y, Chien KL, Hsu HC, Su TC, Chen MF, Lee YT. Use of serum homocysteine to predict stroke, coronary heart disease and death in ethnic Chinese. 12-year prospective cohort study. *Circ J* 2009; **73**(8): 1423-30.
20. Sun Z, Zheng L, Detrano R, et al. An epidemiological survey of stroke among rural Chinese adults results from the Liaoning province. *International Journal of Stroke* 2013; **8(8)**: 701-6.
21. Svensson EH, Abul-Kasim K, Engstrom G, Soderholm M. Risk factors for intracerebral haemorrhage - Results from a prospective population-based study. *European Stroke Journal* 2020; **5(3)**: 278-85.
22. Tsivgoulis G, Psaltopoulou T, Wadley VG, et al. Adherence to a Mediterranean diet and prediction of incident stroke. *Stroke* 2015; **46**(3): 780-5.
23. Watanabe M, Kokubo Y, Higashiyama A, Ono Y, Miyamoto Y, Okamura T. Serum 1,5-anhydro-d-glucitol levels predict first-ever cardiovascular disease: An 11-year population-based Cohort study in Japan, the Suita study. *Atherosclerosis* 2011; **216(2)**: 477-83.
24. Yang Y, Wang A, Yuan X, et al. Association between healthy vascular aging and the risk of the first stroke in a community-based Chinese cohort. *Aging* 2019; **11(15)**: 5807-16.

ix. Paediatric population (n = 1)
1. Christerson S, Stromberg B. Stroke in Swedish children II: Long-term outcome. *Acta Paediatrica, International Journal of Paediatrics* 2010; **99(11)**: 1650-6.

x. Only AVM ICH (n = 1)
1. Van Beijnum J, Lovelock CE, Cordonnier C, et al. Outcome after spontaneous and arteriovenous malformation-related intracerebral haemorrhage: Population-based studies. *Brain* 2009; **132(2)**: 537-43.

xi. No full text available (n = 6)
1. Jiang B, Fang XH, Liu YH, et al. [Prospective study on associations between levels of total cholesterol, triglyceride and risk of ischemic and hemorrhagic strokes]. *Zhonghua Xin Xue Guan Bing Za Zhi* 2010; **38**(3): 268-71.
2. Jucha R. [Stroke incidence and casefatality rates in population of Krosno County]. [Polish]. *Przeglad lekarski* 2013; **70(4)**: 191-4.
3. Klochihina OA, Shprakh VV, Stakhovskaya LV, Polunina EA. [An analysis of the long-term stroke morbidity and mortality in the regions of the Russian Federation included in the Federal patient assistance reorganization program]. *Zh Nevrol Psikhiatr Im S S Korsakova* 2020; **120**(12. Vyp. 2): 37-41.
4. Klochikhina OA, Stakhovskaya LV. An analysis of epidemiological indices of stroke based on the data of a regional population register from 2009 to 2012. *Zhurnal Nevrologii i Psihiatrii imeni SS* 2014; **Korsakova. 2014(6)**: 63-9.
5. Kulesh SD. [Comparative analysis of epidemiology of the first stroke: results of the population register, Grodno, Belarus]. *Zh Nevrol Psikhiatr Im S S Korsakova* 2011; **111**(4 Pt 2): 42-7.
6. Ram A, Libruder C, Hershkovitz Y, Tanne D, Shohat T, Zucker I. [THE ISRAELI NATIONAL STROKE REGISTRY - A TOOL FOR MONITORING MORBIDITY AND ACUTE STROKE CARE IN ISRAEL]. *Harefuah* 2019; **158**(6): 352-6.

xii. Reviews; used for reference list screening (n = 17)
1. Abd-Allah F, Khedr E, Oraby MI, Bedair AS, Georgy SS, Moustafa RR. Stroke burden in Egypt: data from five epidemiological studies. *International Journal of Neuroscience* 2018; **128(8)**: 765-71.
2. Adeloye D. An estimate of the incidence and prevalence of stroke in Africa: A systematic review and meta-analysis. *PLoS ONE* 2014; **9(6) (no pagination)**.
3. Alqahtani BA, Alenazi AM, Hoover JC, et al. Incidence of stroke among Saudi population: a systematic review and meta-analysis. *Neurological Sciences* 2020; **41(11)**: 3099-104.
4. Arnao V, Acciarresi M, Cittadini E, Caso V. Stroke incidence, prevalence and mortality in women worldwide. *International Journal of Stroke* 2016; **11(3)**: 287-301.
5. Feigin VL, Lawes CM, Bennett DA, Barker-Collo SL, Parag V. Worldwide stroke incidence and early case fatality reported in 56 population-based studies: a systematic review. *The Lancet Neurology* 2009; **8(4)**: 355-69.
6. Goulart AC. EMMA study: A Brazilian community-based cohort study of stroke mortality and morbidity. *Sao Paulo Medical Journal* 2016; **134(6)**: 543-54.
7. He F, Blackberry I, Yao L, Xie H, Rasekaba T, Mnatzaganian G. Pooled incidence and case-fatality of acute stroke in Mainland China, Hong Kong, and Macao: A systematic review and meta-analysis. *PLoS ONE* 2022; **17(6 June) (no pagination)**.

8. Ibrahim F, Deleu D, Akhtar N, et al. Burden of Stroke in Qatar. *Journal of Stroke and Cerebrovascular Diseases* 2015; **24(12)**: 2875-9.
9. Jones SP, Baqai K, Clegg A, et al. Stroke in India: A systematic review of the incidence, prevalence, and case fatality. *International Journal of Stroke* 2022; **17(2)**: 132-40.
10. Kamalakannan S, Gudlavalleti ASV, Murthy Gudlavalleti VS, Goenka S, Kuper H. Incidence & prevalence of stroke in India: A systematic review. *Indian Journal of Medical Research* 2017; **146(August)**: 175-85.
11. Khurana S, Gourie-Devi M, Sharma S, Kushwaha S. Burden of Stroke in India during 1960 to 2018: A Systematic Review and Meta-Analysis of Community Based Surveys. *Neurology India* 2021; **69(3)**: 547-59.
12. Kulshreshtha A, Anderson LM, Goyal A, Keenan NL. Stroke in South Asia: A systematic review of epidemiologic literature from 1980 to 2010. *Neuroepidemiology* 2012; **38(3)**: 123-9.
13. Li X, Zhang L, Wolfe CDA, Wang Y. Incidence and Long-Term Survival of Spontaneous Intracerebral Hemorrhage Over Time: A Systematic Review and Meta-Analysis. *Frontiers in Neurology* 2022; **13 (no pagination)**.
14. Li Y, Zhang C, Wu Y, Zhang W. Global burden of stroke in 2010: a pooling analysis of worldwide population-based data on stroke incidence. *Journal of Public Health (Germany)* 2016; **24(6)**: 513-20.
15. Phan HT, Blizzard CL, Reeves MJ, et al. Sex differences in long-term mortality after stroke in INSTRUCT (INternational STRoke oUtComes sTudy). *Circulation: Cardiovascular Quality and Outcomes* 2017; **10(2) (no pagination)**.
16. Prasad K, Vibha D, Meenakshi. Cerebrovascular disease in South Asia - Part I: A burning problem. *JRSM Cardiovasc Dis* 2012; **1**(7).
17. Zhang Y, Yu C, Bao J. Impact of daily mean temperature, cold spells, and heat waves on stroke mortality a multivariate Meta-analysis from 12 counties of Hubei province, China. [Chinese]. *Chinese Journal of Endemiology* 2017; **38(4)**: 508-13.

xiii. Overlapping cohorts (n = 53)

1. Amiri A, Kapral MK, Thrift AG, et al. The Incidence and Characteristics of Stroke in Urban-Dwelling Iranian Women. *Journal of Stroke and Cerebrovascular Diseases* 2018; **27(3)**: 547-54.
2. Baba Y, Ishikawa S, Kayaba K, Gotoh T, Kajii E. High pulse pressure is associated with increased risk of stroke in Japanese: The JMS Cohort Study. *Blood Pressure* 2011; **20(1)**: 10-4.
3. Baptista D, Abreu P, Azevedo E, Magalhaes R, Correia M. Sex Differences in Stroke Incidence in a Portuguese Community-Based Study. *Journal of Stroke and Cerebrovascular Diseases* 2018; **27(11)**: 3115-23.
4. Bejot Y, Aboa-Eboule C, Jacquin A, et al. Stroke care organization overcomes the deleterious 'weekend effect' on 1-month stroke mortality: A population-based study. *European Journal of Neurology* 2013; **20(8)**: 1177-83.
5. Bejot Y, Cordonnier C, Durier J, Aboa-Eboule C, Rouaud O, Giroud M. Intracerebral haemorrhage profiles are changing: Results from the Dijon population-based study. *Brain* 2013; **136(2)**: 658-64.
6. Bejot Y, Osseby GV, Aboa-Eboule C, et al. Dijon's vanishing lead with regard to low incidence of stroke. *European Journal of Neurology* 2009; **16(3)**: 324-9.
7. Bejot Y, Rouaud O, Benatru I, et al. Contribution of the Dijon Stroke Registry after 20 years of data collection. [French]. *Revue Neurologique* 2008; **164(2)**: 138-47.
8. Béjot Y, Rouaud O, Jacquin A, et al. Stroke in the very old: incidence, risk factors, clinical features, outcomes and access to resources--a 22-year population-based study. *Cerebrovasc Dis* 2010; **29**(2): 111-21.
9. Cabral NL, Goncalves ARR, Longo AL, et al. Trends in stroke incidence, mortality and case fatality rates in Joinville, Brazil: 1995-2006. *Journal of Neurology, Neurosurgery and Psychiatry* 2009; **80(7)**: 749-54.
10. Cabral NL, Longo A, Moro C, et al. Education level explains differences in stroke incidence among city districts in Joinville, Brazil: A three-year population-based study. *Neuroepidemiology* 2011; **36(4)**: 258-64.
11. Cabral NL, Nagel V, Conforto AB, et al. Five-year survival, disability, and recurrence after first-ever stroke in a middle-income country: A population-based study in Joinvile, Brazil. *International Journal of Stroke* 2018; **13(7)**: 725-33.
12. Carlsson M, Wilsgaard T, Johnsen SH, et al. The impact of risk factor trends on intracerebral hemorrhage incidence over the last two decades-The Tromso Study. *International Journal of Stroke* 2019; **14(1)**: 61-8.
13. Carlsson M, Wilsgaard T, Johnsen SH, et al. Long-Term Survival, Causes of Death, and Trends in 5-Year Mortality After Intracerebral Hemorrhage: The Tromso Study. *Stroke* 2021: STROKEAHA120032750.
14. Corso G, Bottacchi E, Giardini G, et al. Community-based study of stroke incidence in the Valley of Aosta, Italy - CARe-cerebrovascular aosta registry: Years 2004-2005. *Neuroepidemiology* 2009; **32(3)**: 186-95.
15. Fujii T, Arima H, Takashima N, et al. Seasonal Variation in Incidence of Stroke in a General Population of 1.4 Million Japanese: The Shiga Stroke Registry. *Cerebrovascular Diseases* 2022; **51(1)**: 75-81.
16. Gabet A, Olie V, Bejot Y. Atrial Fibrillation in Spontaneous Intracerebral Hemorrhage, Dijon Stroke Registry (2006-2017). *Journal of the American Heart Association* 2021: e020040.
17. Gall SL, Donnan G, Dewey HM, et al. Sex differences in presentation, severity, and management of stroke in a population-based study. *Neurology* 2010; **74(12)**: 975-81.

18. Ghandehari K. Epidemiology of stroke in Iran. *Galen Medical Journal* 2016; **5(Specialissue)**: 3-9.
19. Gotoh S, Hata J, Ninomiya T, et al. Hematocrit and the risk of cardiovascular disease in a Japanese community: The Hisayama Study. *Atherosclerosis* 2015; **242**(1): 199-204.
20. Han J, Liu J, Wu Y, et al. Long-term trends in the stroke prognosis among rural residents in china: A population-based surveillance study. *Risk Management and Healthcare Policy* 2021; **14**: 4013-21.
21. Icks A, Claessen H, Kvitkina T, et al. Incidence and relative risk of stroke in the diabetic and the non-diabetic population between 1998 and 2014: A community-based stroke register. *PLoS ONE* 2017; **12(11) (no pagination)**.
22. Ishikawa S, Kotani K, Kario K, et al. Inverse association between serum lipoprotein(a) and cerebral hemorrhage in the Japanese population. *Thrombosis Research* 2013; **131(2)**: e54-e8.
23. Jin C, Li G, Rexrode KM, et al. Prospective Study of Fasting Blood Glucose and Intracerebral Hemorrhagic Risk. *Stroke* 2018; **49**(1): 27-33.
24. Kawate N, Kayaba K, Hara M, Kotani K, Ishikawa S. Body mass index and stroke incidence in Japanese community residents: The Jichi Medical School (JMS) Cohort Study. *Journal of epidemiology* 2017; **27(7)**: 325-30.
25. Kõrv L, Vibo R, Kõrv J. Declining first-ever stroke incidence rates in 15- to 54-year-old residents of Tartu, Estonia, from 1991 to 2017. *Int J Stroke* 2023; **18**(4): 462-8.
26. Li W, Jin C, Vaidya A, et al. Blood Pressure Trajectories and the Risk of Intracerebral Hemorrhage and Cerebral Infarction: A Prospective Study. *Hypertension* 2017; **70**(3): 508-14.
27. Loan JJM, Gane AB, Middleton L, et al. Association of baseline hematoma and edema volumes with one-year outcome and long-term survival after spontaneous intracerebral hemorrhage: A community-based inception cohort study. *International Journal of Stroke* 2021; **16(7)**: 828-39.
28. Lu H, Guo Z, Liu J, et al. Trends in stroke incidence among elderly low-income residents of rural China: a population-based study from 1992 to 2016. *Aging* 2018; **10(11)**: 3438-49.
29. Lu H, Ni J, Yang Q, et al. Alcohol Consumption and Stroke Risk in Men: A Population-Based Cohort Study in Rural Tianjin, China. *Neuroepidemiology* 2021: 1-9.
30. Magalhaes R, Silva MC, Correia M, Bailey T. Are stroke occurrence and outcome related to weather parameters? Results from a population-based study in Northern Portugal. *Cerebrovascular Diseases* 2011; **32(6)**: 542-51.
31. Maheswaran R, Pearson T, Smeeton NC, Beevers SD, Campbell MJ, Wolfe CD. Outdoor air pollution and incidence of ischemic and hemorrhagic stroke: a small-area level ecological study. *Stroke* 2012; **43**(1): 22-7.
32. Moutinho M, Magalhaes R, Correia M, Silva MC. A community-based study of stroke code users in Northern Portugal. *Acta Medica Portuguesa* 2013; **26(2)**: 113-22.
33. Ning X, Sun J, Jiang R, et al. Increased stroke burdens among the low-income young and middle aged in Rural China. *Stroke* 2017; **48(1)**: 77-83.
34. Olindo S, Saint-Vil M, Jeannin S, et al. One-year disability, death and recurrence after first-ever stroke in a Black Afro-Caribbean population. *International Journal of Stroke* 2017; **12(8)**: 844-50.
35. Palm F, Santos MD, Urbanek C, et al. Stroke seasonality associations with subtype, etiology and laboratory results in the Ludwigshafen Stroke Study (LuSSt). *European Journal of Epidemiology* 2013; **28(5)**: 373-81.
36. Palm F, Urbanek C, Rose S, et al. Stroke incidence and survival in Ludwigshafen am Rhein, Germany: The Ludwigshafen Stroke Study (LuSSt). *Stroke* 2010; **41(9)**: 1865-70.
37. Qin H, Turnbull I, Chen Y, et al. Hospital management of major stroke types in Chinese adults: a population-based study of 20 000 hospitalised stroke cases. *BMJ Open* 2021; **11(11) (no pagination)**.
38. Rodrigues MA, N ES, Lerpiniere C, et al. Association between Computed Tomographic Biomarkers of Cerebral Small Vessel Diseases and Long-Term Outcome after Spontaneous Intracerebral Hemorrhage. *Annals of Neurology* 2021; **89(2)**: 266-79.
39. Sato F, Nakamura Y, Kayaba K, Ishikawa S. Stroke Risk Due to Smoking Characterized by Sex Differences in Japan: The Jichi Medical School Cohort Study. *Journal of Stroke and Cerebrovascular Diseases* 2022; **31(2) (no pagination)**.
40. Sato F, Nakamura Y, Kayaba K, Ishikawa S. Hemoglobin Concentration and the Incidence of Stroke in the General Japanese Population: The Jichi Medical School Cohort Study. *Journal of epidemiology* 2022; **32(3)**: 125-30.
41. Takashima N, Arima H, Kita Y, et al. Two-Year Survival After First-Ever Stroke in a General Population of 1.4 Million Japanese - Shiga Stroke Registry. *Circ J* 2018; **82**(10): 2549-56.
42. Takashima N, Arima H, Kita Y, et al. Long-term survival after stroke in 1.4 million japanese population: Shiga stroke and heart attack registry. *Journal of Stroke* 2020; **22(3)**: 336-44.
43. Tang L, Li H, Zhang M, et al. Hypertension, alcohol drinking and stroke incidence: A population-based prospective cohort study among inner Mongolians in China. *Journal of Hypertension* 2014; **32(5)**: 1091-6.

44. Tian X, Liu J, Yu C, et al. Long-Term Trends in Stroke Management and Burden Among Low-Income Women in a Rural Area From China (1992-2019): A Prospective Population-Based Study. *Frontiers in Neurology* 2021; **12 (no pagination)**.
45. Tsutsumi A, Kayaba K, Kario K, Ishikawa S. Prospective study on occupational stress and risk of stroke. *Archives of Internal Medicine* 2009; **169(1)**: 56-61.
46. Turin TC, Kita Y, Rumana N, et al. Short-Term Exposure to Air Pollution and Incidence of Stroke and Acute Myocardial Infarction in a Japanese Population. *Neuroepidemiology* 2012; **38(2)**: 84-92.
47. Turin TC, Kita Y, Rumana N, et al. Diurnal variation in onset of hemorrhagic stroke is independent of risk factor status: Takashima stroke registry. *Neuroepidemiology* 2010; **34(1)**: 25-33.
48. Wang J, An Z, Li B, et al. Increasing stroke incidence and prevalence of risk factors in a low-income Chinese population. Neurology 2015; 84(4): 374-81.
49. Wang Y, Rudd AG, Wolfe CDA. Age and ethnic disparities in incidence of stroke over time: The South London stroke register. *Stroke* 2013; **44(12)**: 3298-304.
50. Wu Y, Fan Z, Chen Y, et al. Determinants of developing stroke among low-income, rural residents: A 27-year population-based, prospective cohort study in Northern China. *Frontiers in Neurology* 2019; **10(FEB) (no pagination)**.
51. Yang L, Li L, Millwood IY, et al. Age at menarche and risk of major cardiovascular diseases: Evidence of birth cohort effects from a prospective study of 300,000 Chinese women. *International Journal of Cardiology* 2017; **227**: 497-502.
52. Yang R, Wang A, Ma L, et al. Hematocrit and the incidence of stroke: A prospective, population-based cohort study. *Therapeutics and Clinical Risk Management* 2018; **14**: 2081-8.
53. Yonemoto K, Doi Y, Hata J, et al. Body mass index and stroke incidence in a Japanese community: The Hisayama study. *Hypertension Research* 2011; **34(2)**: 274-9.

Section 4. Overview of studies with request for additional data

We have contacted the authors of 24 different articles, asking for the following: 1) clarification of definitions used; 2) specific numbers depicted in figures or clarification of tables; or 3) additional data on overall crude incidence or number of person-years. We sent two reminders when queries remained unanswered. Eleven authors responded to our emails. We have received additional data from nine cohorts (**in bold**).

i. Clarification of definitions used (n = 4)

1. Goulart AC. EMMA study: A Brazilian community-based cohort study of stroke mortality and morbidity. *Sao Paulo Medical Journal* 2016; **134(6)**: 543-54.
2. Howard VJ, Kleindorfer DO, Judd SE, et al. Disparities in stroke incidence contributing to disparities in stroke mortality. *Annals of Neurology* 2011; **69(4)**: 619-27.
3. Li J, Imano H, Yamagishi K, et al. Leukocyte Count and Risks of Stroke and Coronary Heart Disease: The Circulatory Risk in Communities Study (CIRCS). *J Atheroscler Thromb* 2022; **29**(4): 527-35.
4. **Olofindayo J, Peng H, Liu Y, et al. The interactive effect of diabetes and central obesity on stroke: A prospective cohort study of inner Mongolians. BMC Neurology 2015: 1-7.**

ii. Specific numbers depicted in figures or clarification of tables (n = 6)

1. Aghaali M, Yoosefee S, Hejazi SA, et al. A prospective population-based study of stroke in the Central Region of Iran: The Qom Incidence of Stroke Study. *International Journal of Stroke* 2022; **17(9)**: 957-63.
2. Boden-Albala B, Allen J, Roberts ET, Bulkow L, Trimble B. Ascertainment of Alaska Native Stroke Incidence, 2005-2009: Lessons for Assessing the Global Burden of Stroke. *Journal of Stroke and Cerebrovascular Diseases* 2017; **26(9)**: 2019-26.
3. Chen Y, Wright N, Guo Y, et al. Mortality and recurrent vascular events after first incident stroke: a 9-year community-based study of 0.5 million Chinese adults. The Lancet Global Health 2020; 8(4): e580-e90.
4. Diaz-Guzman J, Egido JA, Gabriel-Sanchez R, Barbera-Comes G, Fuentes-Gimeno B, Fernandez-Perez C. Stroke and transient ischemic attack incidence rate in Spain: The IBERICTUS study. Cerebrovascular Diseases 2012; **34(4):** 272-81.
5. **Gotoh S, Hata J, Ninomiya T, et al. Trends in the incidence and survival of intracerebral hemorrhage by its location in a Japanese community. Circ J 2014; 78(2): 403-9.**
6. Howard G, Cushman M, Howard VJ, et al. Risk factors for intracerebral hemorrhage: the REasons for geographic and racial differences in stroke (REGARDS) study. Stroke 2013; **44(5):** 1282-7.

iii. Additional data on crude incidence or person-years (n = 14)

1. Crichton S, Barratt B, Spiridou A, et al. Associations between exhaust and non-exhaust particulate matter and stroke incidence by stroke subtype in South London. *Science of the Total Environment* 2016; **568**: 278-84.
2. Diaz-Guzman J, Egido-Herrero JA, Fuentes B, et al. Incidence of strokes in Spain: The iberictus study. Data from the pilot study. [Spanish]. *Revista de Neurologia* 2009; **48(2)**: 61-5.
3. **Gardener H, Sacco RL, Rundek T, Battistella V, Cheung YK, Elkind MSV. Race and Ethnic Disparities in Stroke Incidence in the Northern Manhattan Study. Stroke 2020: 1064-9.**
4. **Graber M, Garnier L, Mohr S, et al. Influence of Pre-Existing Mild Cognitive Impairment and Dementia on Post-Stroke Mortality. The Dijon Stroke Registry. Neuroepidemiology 2020; 54(6): 490-7.**
5. **Hata J, Ninomiya T, Hirakawa Y, et al. Secular trends in cardiovascular disease and its risk factors in Japanese: Half-century data from the hisayama study (1961-2009). Circulation 2013; 128(11): 1198-205.**
6. Heuschmann PU, Grieve AP, Toschke AM, Rudd AG, Wolfe CDA. Ethnic group disparities in 10-year trends in stroke incidence and vascular risk factors: The south London stroke register (SLSR). Stroke 2008; **39(8):** 2204-10.
7. **Kleindorfer DO, Khoury J, Moomaw CJ, et al. Stroke incidence is decreasing in whites but not in blacks: A population-based estimate of temporal trends in stroke incidence from the greater cincinnati/northern kentucky stroke study. Stroke 2010; 41(7): 1326-31.**
8. **Kolominsky-Rabas PL, Wiedmann S, Weingartner M, et al. Time trends in incidence of pathological and etiological stroke subtypes during 16 years: The Erlangen stroke project. Neuroepidemiology 2015; 44(1): 24-9.**
9. Li L, Scott CA, Rothwell PM. Association of Younger vs Older Ages With Changes in Incidence of Stroke and Other Vascular Events, 2002-2018. JAMA 2022; **328(6):** 563-74.
10. **Madsen TE, Khoury JC, Leppert M, et al. Temporal Trends in Stroke Incidence Over Time by Sex and Age in the GCNKSS. Stroke 2020; 51(4): 1070-6.**
11. Matsumoto M, Ishikawa S, Kajii E. Cumulative effects of weather on stroke incidence: a multi-community cohort study in Japan. Journal of epidemiology / Japan Epidemiological Association 2010; 20(2): 136-42.
12. **Zahuranec DB, Lisabeth LD, Sanchez BN, et al. Intracerebral hemorrhage mortality is not changing**

despite declining incidence. Neurology 2014; 82(24): 2180-6.
13. Zhang Y, Galloway JM, Welty TK, et al. Incidence and risk factors for stroke in american indians the strong heart study. *Circulation* 2008; **118(15)**: 1577-84.
14. Zhao P, Liu J, Hao Y, et al. Macroeconomic Development and Dramatic Increase in Stroke Burden in Rural China: A 25-Year Population-Based Study. Frontiers in Neurology 2020; **11** (no pagination).
